# Speciation of Potentially Carcinogenic Trace Nickel(II) Ion Levels in Human Saliva: A Sequential Metabolomics-Facilitated High-Field ^1^H NMR Investigation

**DOI:** 10.3390/metabo15010004

**Published:** 2024-12-30

**Authors:** Kayleigh Hunwin, Georgina Page, Mark Edgar, Mohammed Bhogadia, Martin Grootveld

**Affiliations:** Leicester School of Pharmacy, De Montfort University, Leicester LE1 9BH, UK; p2421485@my365.dmu.ac.uk (K.H.); p16198195@my365.dmu.ac.uk (G.P.); mark.edgar@dmu.ac.uk (M.E.);

**Keywords:** speciation, nickel(II), nickel(II) complexants and chelators, NMR analysis, human saliva, dental implant, nickel-containing dental alloys, endogenous ligands, nickel toxicity, carcinogenicity of nickel compounds

## Abstract

**Introduction/Objectives:** Since the biological activities and toxicities of ‘foreign’ and/or excess levels of metal ions are predominantly determined by their precise molecular nature, here we have employed high-resolution ^1^H NMR analysis to explore the ‘speciation’ of paramagnetic Ni(II) ions in human saliva, a potentially rich source of biomolecular Ni(II)-complexants/chelators. These studies are of relevance to the *in vivo* corrosion of nickel-containing metal alloy dental prostheses (NiC-MADPs) in addition to the dietary or adverse toxicological intake of Ni(II) ions by humans. **Methods:** Unstimulated whole-mouth human saliva samples were obtained from n = 12 pre-fasted (≥8 h) healthy participants, and clear whole-mouth salivary supernatants (WMSSs) were obtained from these via centrifugation. Microlitre aliquots of stock aqueous Ni(II) solutions were sequentially titrated into WMSS samples via micropipette. Any possible added concentration-dependent Ni(II)-mediated pH changes therein were experimentally controlled. ^1^H NMR spectra were acquired on a JEOL JNM-ECZ600R/S1 spectrometer. **Results:** Univariate and multivariate (MV) metabolomics and MV clustering analyses were conducted in a sequential stepwise manner in order to follow the differential effects of increasing concentrations of added Ni(II). The results acquired showed that important Ni(II)-responsive biomolecules could be clustered into distinguishable patterns on the basis of added concentration-dependent responses of their resonance intensities and line widths. At low added concentrations (71 µmol/L), low-WMSS-level N-donor amino acids (especially histidine) and amines with relatively high stability constants for this paramagnetic metal ion were the most responsive (severe resonance broadenings were observed). However, at higher Ni(II) concentrations (140–670 µmol/L), weaker carboxylate O-donor ligands such as lactate, formate, succinate, and acetate were featured as major Ni(II) ligands, a consequence of their much higher WMSS concentrations, which were sufficient for them to compete for these higher Ni(II) availabilities. From these experiments, the metabolites most affected were found to be histidine ≈ methylamines > taurine ≈ lactate ≈ succinate > formate > acetate ≈ ethanol ≈ glycine ≈ N-acetylneuraminate, although they predominantly comprised carboxylato oxygen donor ligands/chelators at the higher added Ni(II) levels. Removal of the interfering effects arising from the differential biomolecular compositions of the WMSS samples collected from different participants and those from the effects exerted by a first-order interaction effect substantially enhanced the statistical significance of the differences observed between the added Ni(II) levels. The addition of EDTA to Ni(II)-treated WMSS samples successfully reversed these resonance modifications, an observation confirming the transfer of Ni(II) from the above endogenous complexants to this exogenous chelator to form the highly stable diamagnetic octahedral [Ni(II)-EDTA] complex (K_stab_ = 1.0 × 10^19^ M^−1^). **Conclusions:** The results acquired demonstrated the value of linking advanced experimental design and multivariate metabolomics/statistical analysis techniques to ^1^H NMR analysis for such speciation studies. These provided valuable molecular information regarding the identities of Ni(II) complexes in human saliva, which is relevant to trace metal ion speciation and toxicology, the *in vivo* corrosion of NiC-MADPs, and the molecular fate of ingested Ni(II) ions in this biofluid. The carcinogenic potential of these low-molecular-mass Ni(II) complexes is discussed.

## 1. Introduction

Nickel, which ranks as the 24th most abundant element naturally distributed in the Earth’s crust, now represents an essential element for biosystems, this requirement involving a series of important processes required for the healthy growth of humans, animals, plants, and microorganisms [1,2,3]. In view of the rapidly enhancing growth and preponderance of commercial and industrial arrogations for this metal and its compounds over the last 30 years or so, an enormous expansion in applications such as electronics, electroplating, catalysis, batteries, ceramics, pigments, and coinage has been experienced, as indeed it has for stainless steel and further nickel alloys [4]. This has led to higher industrial demands for it, and hence, it is mined, extracted, and commercially employed at a more rapid rate. However, this has also adversely given rise to its enhanced level of anthropogenic emission due to the consequent combination of industrial waste disposal and fossil fuel combustion. Therefore, technically, it may be described as a pollutant of the environment, and it is deposited at higher concentrations into the air, water, and soil compartments. Notably, refinery workers exposed to Ni-sub-sulphides may develop respiratory tract cancers [5], and within cells, carcinogenesis can be induced by Ni ions through epigenetic modifications [5,6]. Further reports on the toxicity and adverse health effects induced by nickel compounds are available in Refs. [7,8,9].

Similarly, the intra-oral corrosion of Ni-containing dental alloys, a process which releases Ni(II) primarily into their placement-adjacent environments, may cause adverse health effects. To date, it has been well known that the most significant clinical risk from Ni-containing dental alloys is hypersensitivity reactions in patients who are susceptible, and a series of clinical investigations has documented this [10,11]. Such deleterious effects are associated with the corrosion of these alloys. Hence, the dental application of such alloys should certainly not be utilised in subjects who are hypersensitive. Intriguingly, controversy abounds in this field, and one relevant consideration is whether Ni-based alloys give rise to hypersensitivity in non-sensitive subjects or whether hypersensitivity reactions stem from cross-reactions in those who are polysensitive. Additional clinical risks, including cytotoxicity, inflammation, and oxidative stress, have been indicated by results obtained in vitro. However, Ni(II) ions liberated therefrom via *in vivo* corrosion processes do exert potent pro-inflammatory effects and are also known to propagate oxidative stress in cell culture experiments [10]. Notwithstanding, it is still not known if any form of intra-oral inflammation arises from, or is exacerbated by, the Ni(II) released from Ni-containing dental alloys as a corrosion product.

Moreover, currently, there are no significant research data available that these alloys and Ni-containing corrosion products, including Ni(II) ion complexes, are actually carcinogenic. However, with regard to the corrosion process, there currently remains little or no information available on the precise molecular nature of the Ni(II) species derived therefrom, i.e., regarding the following questions: What is/are the molecular structure(s) of Ni(II) complexes formed from these liberated ions; more specifically, do bioavailable ligands and/or chelators complex them, and if so, which ones are considered important under these conditions? Which complexes are actually formed at physiologically relevant pH values? Do the complexants involved have low or high molecular masses (e.g., proteins for the latter)? Does their structure and electronic charge, if any, permit them to cross cell membranes into the cytosol, where they might then exert any toxic or carcinogenic effects following their entry into sub-cellular compartments?

The importance of metal ion complexation/chelation processes in biosystems is exemplified by the range of different types of bioinorganic chemical reactions involved (predominantly dissolution-assisted, redox, and ligand-exchange activities), which may be limited by their restricted solubilities at physiological pH values. Such processes must be functioning advantageously and/or countered when metal ions such as Ni(II) are taken up by cells. In mammals, the major classes of these complexants include organic acid anions with oxygen donor atoms such as lactate, propionate, and succinate; amino acids containing both oxygen and nitrogen donor atoms, e.g., histidine, glycine, alanine, lysine, and arginine; peptides with oxygen, nitrogen, and/or sulphur donor atoms, the latter including glutathione (GSH); and nucleotides with nitrogen and phosphate–oxygen donor atoms. Moreover, there are, of course, a series of high-molecular-mass protein species such as cysteine-rich metallothioneins localised within the Golgi apparatus membrane [12], which play important roles in the detoxification of metal ions such as cadmium(II) (Cd(II)). Although the liberation of nickel ions from dental alloy materials has been previously explored under different experimental conditions, the overall clinical significance of these in vitro laboratory-based investigations remains complicated. Notably, one major consideration is simply the composition of the solution medium utilised, and experiments featuring the use of synthetic (artificial) rather than real saliva, preferably human, have certainly proven that there are indeed major differences observed between these equilibration media, perhaps the most important being the availability of metal ion-complexing or -chelating biomolecules such as amino acids; carboxylic acid anions such as lactate, succinate, and formate; and relatively high levels of the metal ion-ligating thiocyanate anion (SCN^−^) present in this biofluid [13,14]. Indeed, these complexants can also exert major influences on the shifting thermodynamic equilibria involved from the solid (metallic Ni(0)) to its solution (presumably, Ni(II) ion) states.

In the bioinorganic chemistry research field, Ni(II) complexes are of special interest in view of the quite wide range of spatial configurations possible for them, notably, coordination numbers of four or six [15]. When the coordination number is four, strong-field ligands yield square-planar complex configurations that are diamagnetic with dsp^2^ hybridisation, whereas weaker-field ligands give rise to tetrahedral complex configurations with sp^3^ hybridisation. In contrast, when the coordination number is six, octahedral complex structures are involved, and these are diamagnetic or paramagnetic with strong- and weak-field ligands, respectively (featuring d^2^sp^3^ and sp^3^d^2^ hybridisation, respectively).

Our research group have been forerunners in the early history of NMR-linked salivary metabolomics research and on the applications of high-resolution NMR and NMR-based metabolomics analysis to the dental/oral health research area in general [16,17]. Furthermore, high-resolution NMR technologies have been successfully employed previously by the applicant’s group for the speciation of metal ions in a range of biofluids for many years, e.g., [18,19], although to date, studies based on human saliva have been somewhat limited.

Therefore, for the first time, here we report Ni(II) ion-mediated alterations to the ^1^H NMR profiles of a broad spectrum of possible metabolic complexants for this metal ion present in human whole-mouth salivary supernatants (WMSSs), a laboratory strategy which has the potential to provide valuable information concerning the precise chemical nature and molecular structure of solution-phase Ni(II) complexes in this biofluid. Such complexes may be derived from the oral ingestion of dietary sources of this metal ion. However, these evaluations are also of much relevance to the liberation of this metal ion from the *in vivo* corrosion of metal alloy dental prostheses (MADPs), which represent rich, albeit ‘foreign’, sources of it [20]. When applied to the biological speciation of metal ions, the high-field NMR technique offers many worthwhile advantages over alternative bioanalytical methods, most especially since it has the capacity to simultaneously monitor the metal ion-complexing abilities of many different salivary biomolecules in a virtually non-invasive manner.

We also demonstrate that the incorporation of advanced experimental designs featuring both univariate and multivariate (MV) metabolomics strategies coupled with the ‘stepwise’ added Ni(II) concentration-dependent monitoring of its complex analyte identities arising therefrom using high-field ^1^H NMR spectroscopy serves as a major asset to such speciation investigations. Indeed, we have taken steps to determine the molecular structure of natural Ni(II) complexants/chelators available in WMSS samples at individual levels of added Ni(II), most especially those detectable at the lowest available concentration, since the added quantities of Ni(II) employed here are greater than those commonly encountered *in vivo* [21]. Furthermore, we reveal that the isolation and removal of the potentially interfering components of variance ascribable to differences between participants and the first-order ‘between-added Ni(II) concentrations x between-participants’ interaction effect substantially increases the precision and, hence, the statistical significance of the ‘between-added Ni(II) level’ evaluations explored, and hence, customises the model for this important fixed-effect factor.

These investigations are of further relevance to trace metal ion research in general, specifically, the biomolecular fate of ingested Ni(II) in human saliva along with the dependence of its potential carcinogenic properties on the speciation status of its complexes in this biofluid. Indeed, the biological activities of metal ions are known to be critically determined by their precise molecular natures in variable tissue and biofluid locations [18,19]. Notably, trace levels of Ni are readily detectable in human saliva [22,23] and can be determined therein in order to monitor its dietary or environmental intake together with its possible involvement in a wide range of diseases, oral or otherwise.

## 2. Materials and Methods

### 2.1. Sample Collection and Preparation

Unstimulated human saliva samples were obtained from a total of 12 healthy, non-medically compromised participants, consisting of 8 women and 4 men; the mean ± SD age of participants was 23.58 ± 2.31 years. Full ethical approval for this investigation was obtained from the Faculty of Health and Life Sciences Research Ethics Committee of De Montfort University (approval reference number 457249). Prior to their participation in the study, all volunteers received a participant information sheet (PIS) outlining the nature of the research project being conducted, including its purpose, as well as highlighting the manner in which samples should be collected effectively. In addition to this, the dental and medical treatment history of each individual was disclosed in confidence in order to confirm that none of these participants had any existing Ni- or other metal ion-containing dental implants or amalgams present in their oral environments, including removable partial dentures.

On pre-selected sampling days, participants were requested to collect all saliva available (usually 5–10 mL), referred to as ‘whole’ saliva expectorated from the mouth (WMS), into a plastic universal collection tube. To ensure that no exogenous agents were introduced into the oral environment, since these could significantly interfere with the study, participants were instructed to collect the sample immediately after awakening in the morning. For this purpose, all participants were requested to refrain completely from oral activities for a period of at least 8 h prior to sample collection, such activities including drinking, eating, smoking, tooth brushing, and oral rinsing, for example. Abstinence from these activities was also ensured during the short period between awakening and sample provision, which was approximately 5 min. Participants were also requested not to consume any alcoholic beverages 24 h prior to WMS sample collection.

All saliva specimens were transported to the laboratory on ice, where they were centrifuged immediately on their arrival (10,000 rpm for a 10 min duration) to remove any cells or debris that may have been present therein to yield whole-mouth salivary supernatant (WMSS) samples. Any supernatants that were not undergoing NMR-based Ni(II) titration experiments immediately were stored at −80 °C until required, with a maximum storage duration time of 60 h. Following the above protocols, including their Ni(II) ion treatment regimen specified below, samples were then placed on the NMR spectrometer autosampler at 25 °C, the mean ± SD time period that they remained there prior to analysis being 6 ± 2, 9 ± 3, and 18 ± 6 h for replicates 1, 2, and 3, respectively. This approach also permitted an unconventional, albeit crude, method for evaluating equilibration time for the completion of reactions involving the interaction of added Ni(II) with multiple ranges of WMSS complexants at this temperature. This triplicate analysis of each prepared WMSS sample also served to monitor ‘between-NMR-run’ reproducibility. In comparison with the protein concentration of human blood plasma (65–83 g/L [24]), that of human saliva is much lower, typically 1.40–6.40 g/L [25]. Therefore, the observation and electronic integration of sharp low-molecular-mass biomolecule resonances were sufficiently enabled in view of the lack of interference arising from the presence of only the minimal macromolecular broad resonance envelope arising from salivary macromolecules, including proteins. Notwithstanding, in order to ensure this for our study, the **WA**ter **S**uppression with a **T**ransverse relaxation filter that **E**liminates **D**istortion (WASTED) pulse sequence [26] was utilised. However, it is important to note that only the non-macromolecular-bound portions of these components are reflected in the determined biomolecule concentrations; i.e., they are expected to be marginally lower in comparison with their total salivary levels in consideration of this biofluid’s low protein content [25].

Aliquots of WMSS specimens (0.70 mL) were ‘spiked’ with increasing added µL volumes (5–50 µL) of a stock solution of 10.00 mmol/L nickel(II) chloride (Fisher Scientific Ltd., Loughborough, UK) in HPLC-grade water. Untreated WMSS samples underwent ^1^H NMR analysis alongside the Ni(II)-spiked samples to serve as essential controls for each experiment. Once spiked, all samples were mixed thoroughly with a vortex mixer to ensure homogenisation before undergoing centrifugation again (10,000 rpm for 20 min) to remove any possible precipitative deposits arising from the added metal ion; however, no such precipitation was observed for added Ni(II) at the above concentrations.

Routinely, 0.06 mL aliquots of D_2_O, containing sodium 3-trimethylsilyl [2,2,3,3-^2^H_4_] propionate (TSP) (both Fisher Scientific Ltd., Loughborough, UK) at a concentration of 2.90 mmol/L, were added to the above untreated control or Ni(II)-treated WMSS samples (final TSP level 229 µmol/L for those which were untreated, and marginally decreased 227.5, 226, 223, 220, 217.5, and 215 µmol/L values for those titrated with 5, 10, 20, 30, 40, and 50 µL aliquots of the stock Ni(II) solution, respectively). In relation to ^1^H NMR analysis, TSP served as both a ^1^H NMR chemical shift reference (δ = 0.00 ppm) and an internal standard for the purpose of some quantitative NMR (QNMR) experiments. Although presenting only a minor analytical complication with a negligible maximal dilution level of only 6%, the sequentially dependent, slightly lower final TSP concentrations of the Ni(II)-treated WMSS solutions satisfactorily negated the small dilution effect encountered via the addition of small volumes of this stock metal ion solution to 0.70 mL volumes of WMSS samples. Uniform volumes of these samples (0.60 mL) were then removed and transferred to 5 mm diameter NMR tubes for ^1^H NMR analysis (Section 2.2).

Any concerns regarding possible pH changes arising from the titration of marginally acidic Ni(II)_(aq.)_ stock solutions (10.00 mmol/L, final pH 6.53) into naturally buffered WMSS samples were circumvented as outlined in Section S1 (Supplementary Information). Additionally, the potential ability of salivary proteins and perhaps further macromolecules therein to broaden the TSP’s internal standard resonance through protein-binding equilibria involving its negative charge and/or other physicochemical attractive phenomena were explored by the method described in Section S2.

However, in view of the affinity of Ni(II) ions for carboxylato O-donor ligands, we also preliminarily monitored any enhancements of TSP’s line width at half-height (Δ*v*_1/2_) values (Hz) with increasing added Ni(II) concentrations in WMSS samples (Section S3 and Figure S3.1). Since the only non-significant difference found was that observed between the 0 (control) and the 71 µmol/L concentration of added Ni(II), we elected to retain TSP as a quantitative internal standard agent for selected further studies only (Section 3.5 and Section 3.6.3). Therefore, for the relative quantification of WMSS sample biomolecule signals, in all other experiments conducted, the probabilistic quotient normalisation (PQN) or constant sum normalisation (CSN) strategies, which do not require reference to an added quantitative internal standard, were utilised (details available in Section 2.3 below).

#### 2.1.1. Quality Control Monitoring of WMSS Samples for ^1^H NMR Analysis

Additionally, Ni(II) speciation experiments were also performed on a sample ‘pooled’ from n = 5 WMSS samples collected from different participants for quality control (QC) purposes, as recommended in [26]. This pooled QC standard was prepared by the admixing of 2.00 mL volumes of each sample (final volume 10.00 mL). Three separate aliquots of this admixed pooled QC sample were treated with increasing Ni(II) concentrations as described above (again, final levels of 0, 71, 140, 280, 410, 540, and 670 µmol/L were added) and then processed and stored for ^1^H NMR analysis in exactly the same manner as our individual WMSS samples; 63 samples in total were analysed. Each ^1^H NMR profile was acquired in triplicate.

The untreated pooled control sample was also randomly inserted in the NMR autochanger device during the complete acquisition of the ^1^H NMR profiles of n = 5 out of 12 of the individual WMSS samples to ensure homogeneity of their compositions between analysis schedules. Between-assay coefficients of variance (CV) values for the determination of selected metabolites, e.g., lactate, acetate, formate, succinate, and ethanol (PQN-normalised), were all ≤6.1%.

### 2.2. ^1^H NMR Measurements

Proton (^1^H) NMR measurements on the above prepared human WMSS samples were conducted on a JEOL 600 MHz NMR facility (JNM-ECZ600R/S1 spectrometer, De Montfort University, Leicester, UK) operating at a frequency of 600.17 MHz and with a probe temperature of 298 K. For each acquired spectrum, the ^1^H NMR acquisition parameters were 256 scans with 4 pre-scans, 16,384 data points, a 1.00 s relaxation delay, a 1.82 s acquisition time for each free induction decay (FID), and a sweep width of 9.00 kHz. The WASTED-II pulse sequence was employed for the acquisition of spectra [27], for which a pulse power of 8.3 dB, a pulse duration of 7.5 μs, and an irradiation frequency of δ = 4.68 ppm was used for the suppression of the broad, intense residual H_2_O/HOD signal. An exponential line-broadening function of 0.20 Hz was applied to FIDs prior to Fourier transformation of them to spectral profiles using zero filling and auto phase.

All spectra were manually referenced, phased, and baseline corrected, with chemical shift values being referenced to that of the internal TSP standard introduced into the samples during the preparation stages. The use of a sample autochanger carousel enabled continuous delivery of the samples (at random), allowing spectra to be acquired in an automated manner, although this randomisation process was stratified somewhat in accordance with those within each of the three analysis-time-dependent replicate groups of samples only, as described above in Section 2.1. Reference ^1^H NMR spectra of metabolites available in supporting databases such as the *Human Metabolome Database* (*HMDB*) [28] and in the published literature [17,29] facilitated these assignments. *ChenomX* software (*ChenomX* NMR suite, Professional Edition (2023)) (ChenomX Inc., Edmonton, AB, Canada) was also employed to confirm ^1^H NMR resonance assignments.

The acquisition of two-dimensional (2D) ^1^H-^1^H correlation spectroscopy (COSY) also served to verify assignments for many of the resonances detectable in the ^1^H NMR profiles of WMSS samples. These spectra were also acquired on the JNM-ECZ600R/S1 600 MHz spectrometer using only 64 scans and 256 slices with low-power pre-saturation to suppress the water resonance (69.2 dB attenuation) at a temperature of 25 °C, p-90 (7.5 µs), sweep width 12.00 ppm, and 2K data points in F2 dimension with an acquisition time of 0.227 s and a relaxation delay of 1.0 s, which yielded a total acquisition time of just under 6 h.

### 2.3. Preprocessing of Salivary ^1^H NMR Data

Chemical shifts were internally referenced to the -Si(CH_3_)_3_ signal of TSP (δ = 0.00 ppm). The -CH_3_ group resonances of acetate (*s*, δ = 1.920 ppm), alanine (*d*, δ = 1.487 ppm), and lactate (*d*, δ = 1.330 ppm) served as secondary internal chemical shift references in all non-Ni(II) ion-treated samples analysed. The identities of all metabolite resonances present in the complete salivary ^1^H NMR profiles acquired were routinely assigned via a consideration of chemical shift values, coupling patterns, and coupling constants, and each of these was then cross-checked with actual or predicted 600 MHz spectra available in the *HMDB*. WMSS sample dataset matrix generation was performed through the application of macro procedures (JEOL *Delta 5* software v5.3.1). An exponential default line-broadening function of 0.20 Hz was applied to FIDs before their Fourier transformation to spectral profiles using zero filling and auto phase. Moreover, gradient shimming was applied, and the optimised shim values (Z1 = −358.99 Hz; Z2 = 180.89 Hz; Z3 = −54.77 Hz; Z4 = −46.93 Hz; Z5 = −8.02 Hz; Z6 = 14.34 Hz; Z7 = 0.00 Hz; and Z8 = 0.00 Hz) were followed and recorded for each individual ^1^H NMR spectral dataset (standard JEOL NMR data format). The JEOL automatic polynomial baseline correction was applied to the spectra.

The above tasks were followed by the employment of an independent macro for a pre-fixed 0.04 ppm bucketing processing subroutine. These work tasks were conducted with the ACD/Labs *Spectrus Processor 2022 and 2023* software packages (ACD/Labs, Toronto, ON, Canada M5C 1T4). Prior to performance of this fixed bucketing process, all spectral profiles were visually examined for any distortions and, where required, were manually corrected. The residual H_2_O/HOD signal (δ = 4.65–5.16 ppm) region remaining following application of the WASTED pulse sequence was removed from all spectral profiles prior to both univariate and multivariate (MV) statistical analysis.

Fixed 0.04 ppm chemical shift buckets were employed in this study rather than the usual ‘intelligent bucketing’ processing subroutine, since this permitted the possible observation of added Ni(II)-induced resonance broadenings that stretched to directly adjacent resonance buckets as well as their original (albeit major) ones. Hence, this process involved the monitoring of decreases in the intensities of their original, untreated control sample ^1^H NMR signal buckets arising from the effects of added Ni(II), coupled with any corresponding increases in those located directly upfield or downfield of them, if indeed not completely localised to the resonance-centred 0.04 ppm bucket itself. Similarly, any added Ni(II)-mediated, slow ligand-exchange modifications in biomolecule signal chemical shift values were also considered to be more readily observable when using this form of bucketing, with the presumption that the change observed features up- or downfield shifts of these to adjacent or perhaps even remote paramagnetically shifted buckets.

The above preprocessing strategy yielded a global table of fixed 0.04 ppm bucket intensities, which was then transferred to MS Excel for additional manipulation. For MV data analysis, fixed-width salivary bucket intensities were ***not*** normalised to that of the TSP resonance, but were instead row-wise probabilistic quotient normalised (PQN) [30], as described below, in view of the significant broadening of TSP’s reference signals observed at added Ni(II) levels ≥ 140 µmol/L. Uniform corrections for the very small dilution effect engendered by added Ni(II)_(aq.)_ at titration volumes of up to 50 µL were made by the method noted above (Section 2.1). Bucket widths for each of the above-referenced acetate-, alanine-, and lactate-CH_3_ group resonances were determined within the chemical shift value ranges of 1.92–1.96 ppm, a combination of 1.44–1.48 and 1.48–1.52 ppm, and 1.32–1.36 ppm, respectively. These datasets were then generalised logarithm (glog)-transformed and Pareto-scaled before conducting MV data analysis. Subsequently, all univariate and MV data analysis models featured row-wise PQN using the between-participants mean spectral profile of all n = 12 untreated WMSS samples as a normalisation reference matrix input. This approach was considered to be the most appropriate in view of the importance of considering the analysis of a pooled WMSS sample as a QC monitoring standard during metabolomics analysis [26]; in this case, a ‘computer-pooled’ median spectral profile for all the (untreated) zero control samples (n = 12) was employed. However, the CSN normalisation process was also applied where specified.

The 0.04 ppm ^1^H NMR bucket margins are depicted as δ = 0.92–0.96 ppm, δ = 1.32–1.36 ppm within the main manuscript, or as [0.92 .. 0.96], [1.32 .. 1.36], etc. in the tables or figures, and indicated as such, where required.

### 2.4. Univariate and Multivariate Analysis of ^1^H NMR-Based Metabolomics Data

Both univariate and MV statistical analysis strategies were performed using *MetaboAnalyst 6.0* software options (University of Alberta and National Research Council, National Institute for Nanotechnology (NINT), Edmonton, AB, Canada). For the former (statistical analysis [one factor] module), a basic one-way analysis of variance (ANOVA) model (model 1) was primarily employed to detect the false discovery rate (FDR)-corrected significance of differences between the mean ^1^H NMR bucket intensity values observed at increasing concentrations of added Ni(II) ions (71–670 µmol/L added to 0.70 mL of n = 12 different untreated WMSS samples collected from n = 12 different donors prior to the addition of 0.06 mL of the ^1^H NMR analysis-required TSP/^2^H_2_O solution). The FDR-corrected *p* values were determined, and then violin plots were generated for all those which attained an FDR-corrected *p* value of <0.05. Subsequently, principal component analysis (PCA) was applied to determine the contributions of the differing added Ni(II) levels for each NMR bucket variable, along with individual participants, to load onto different principal components (PCs), i.e., as determined loading vectors to PCs 1–5. Additional higher-order UV ANOVA-based experimental designs, both fixed-effect and mixed models, were performed using *XLSTAT2014* and *2020* modules (Addinsoft, Paris, France). Outlines of the univariate and MV statistical analysis methods employed in this investigation are provided in Ref. [31].

These data analysis protocols were followed in order to explore sequential spectral changes occurring at each increasing Ni(II) concentration added. For this purpose, primarily two-comparator PLS-DA models were constructed for evaluating such modifications for each of the 71 vs. 0.00, 140 vs. 0.00, 280 vs. 0.00, 410 vs. 0.00, 540 vs. 0.00, and 670 vs. 0.00 contrasts, and variable importance parameters (VIPs) were computed for each of the key carboxylate anion complexants (datasets were PQN or CSN-normalised, glog-transformed, and Pareto-scaled prior to analysis). Subsequently, such VIP values were also computed for key metabolites involved in Ni(II) complexation in models which compared WMSSs containing the lowest added concentrations of Ni(II) with the zero control specimens only, again as two-comparator systems, i.e., 71 µmol/L vs. 0.00, 140 µmol/L vs. 0.00, etc. In this manner, low concentration Ni(II) complexants which were efficient at low added levels in WMSSs, such as selected amino acids and amines, could be successfully evaluated and their contributions towards these ^1^H NMR phenomena assessed.

Furthermore, paired sample *t*-tests, which successfully avoided any confounding effects exerted by the between-participants variance component, were also utilised for these two group comparator models (univariate in this case), with only single, low-added-Ni(II) level groups being evaluated against that of the untreated control.

Additionally, we were able to analyse the TSP-normalised dataset, but only for the lowest added Ni(II) concentration (71 µmol/L) and the zero control groups, since at this level there was not a statistically significant rise in the Δ*v*_1/2_ value of the ^1^H NMR resonance of the TSP internal standard subsequent to glog transformation and Pareto scaling (Section S3). This comparison was made using a paired *t*-test.

Also, an agglomerative hierarchal clustering (AHC)-facilitated heatmap displaying colour-coded individual ^1^H NMR bucket responses to increasing added Ni(II) levels was performed on a CSN-normalised dataset. Indeed, this supporting AHC analysis was employed to support investigations of added Ni(II) concentration-dependent changes to the spectral profiles of WMSS samples and whether any ^1^H NMR bucket clusters were distinctive from each other. Response classes arising therefrom (classes 1–4) were further characterised by the acquisition of ANOVA-assisted aggregated plots of normalised mean resonance intensities versus added Ni(II) levels.

Subsequently, we conducted an evaluation of both one- and two-way ANOVA models, the former with only the ‘between-added Ni(II) concentrations’ effect considered (as model 1 above), the latter with this and the between-participants effect also incorporated (model 2), and with and without the inclusion of a first-order ‘between-added Ni(II) concentrations × between-participants’ interaction effect, i.e., an extension of model 2 (model 3). In this manner, we were able to perform covariate adjustments for determining the statistical significance of the ‘between-added Ni(II) concentrations’ source of variation for all ^1^H NMR bucket variables both in the presence and absence of the potentially confounding between-participants component of variance in this model, the former both with (model 3) and without (model 2) the ‘between-added Ni(II) level x between-participants’ first-order interaction effect considered. For these comparisons, datasets were again PQN-normalised, glog-transformed, and Pareto-scaled, and *p* values determined were Bonferroni-corrected but not FDR-corrected.

Finally, we applied a MV statistical analysis *MetaboAnalyst 6.0* module to conduct an ANOVA simultaneous component analysis (ASCA) model in order to evaluate the MV significance of the added Ni(II) level and between-participants main factors (fixed and random effects, respectively), together with that of their first-order interaction simultaneously. This process involved the upload of an additional metadata table featuring the codes of participants involved together with added Ni(II) concentrations considered. ANOVA-based multifactorial interactive PCA score plots and heatmaps were also generated on the dataset evaluated, which was also PQN-normalised, glog-transformed, and Pareto-scaled.

## 3. Results

### 3.1. One-Dimensional ^1^H NMR Analysis of WMSS Samples

**Figure 1:**
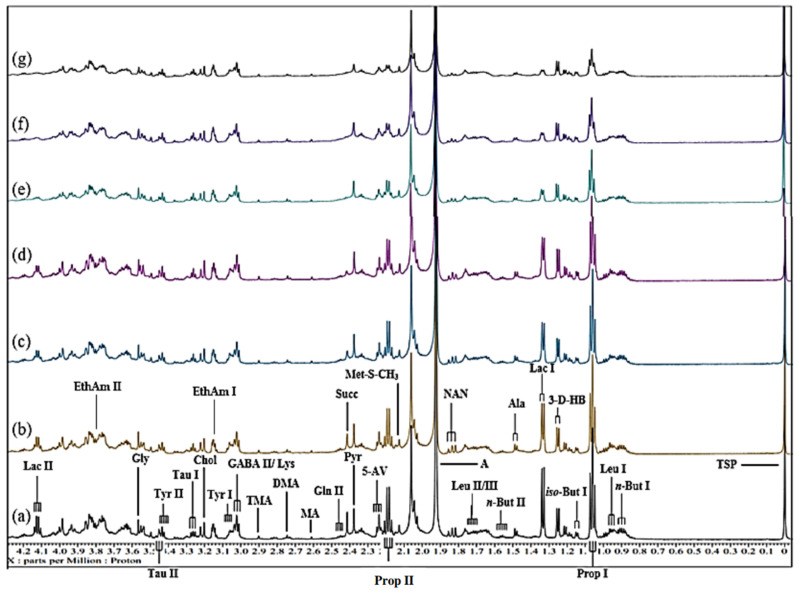

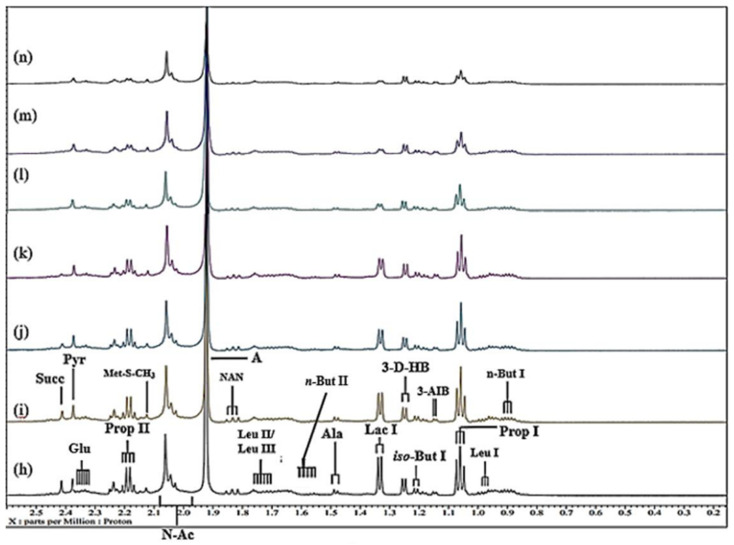
Expanded 0.00–4.25 ppm region of the 600.17 MHz ^1^H NMR spectra of (**a**) a control human salivary supernatant (WMSS) sample and the same sample following equilibration with Ni(II) at added concentrations of (**b**) 71 µmol/L, (**c**) 140 µmol/L, (**d**) 280 µmol/L, (**e**) 410 µmol/L, (**f**) 540 µmol/L, and (**g**) 670 µmol/L (top profile) with (**h**–**n**) showing the expanded 0.16–2.60 ppm regions of the spectra displayed in (**a**), (**b**), (**c**), (**d**), (**e**), (**f**), and (**g**), respectively (bottom profile). Typical spectra are shown. Abbreviations: A, acetate-CH_3_; 3-AIB, 3-aminoisobutyrate-CH_3_; Ala, alanine-CH_3_; 5-AV, 5-aminovalerate-δ-CH_2_; 3-D-HB, 3-D-hydroxybutyrate-CH_3_; *iso*-But I, *iso*-butyrate-CH_3_s; *n*-But I and II, *n*-butyrate-CH_3_ and β-CH_2_ protons, respectively; EthAm-I and -II, ethanolamine-CH_2_NH_2_ and -CH_2_OH, respectively; GABA-II, γ-aminobutyrate γ-CH_2_; Glu, glutamate-γ-CH_2_; Gln II, glutamine-γ-CH_2_; Gly, glycine-CH_2_; Lac I and II, lactate-CH_3_ and -CH protons, respectively; Leu-I, II, and III, leucine-CH_3_s, β-CH, and γ-CH_2_, respectively; Lys, lysine ε-CH_2_ group; MA, DMA, and TMA, -N(CH_3_)_n_ group resonances of methylamine, dimethylamine, and trimethylamine, respectively; Met-S-CH_3_, methionine-S-CH_3_; N-Ac, region for acetamido methyl groups (i.e., -NHCOCH_3_) of N-acetylsugars (the sharper signals are conceivably ascribable to free sugars or to low-molecular-mass oligosaccharide fragments containing these sugars and the broader one(s) to those present in the molecularly mobile portions of N-acetylated glycoproteins (GlycA signal)); NAN, free N-acetylneuraminate (sialate)-C3H (β) resonance; Prop-I and II, propionate-CH_3_ and -CH_2_, respectively; Pyr, pyruvate-CH_3_; Succ, succinate-CH_2_s; Tau I and II, taurine-CH_2_NH_2_ and ^−^O_3_SCH_2_− proton resonances, respectively; Tyr I and II, tyrosine-β-CH_2_ and α-CH protons, respectively; TSP, trimethylsilyl-[2,2,3,3-^2^H_4_] propionate-Si(CH_3_)_3_.

**Figure 2:**
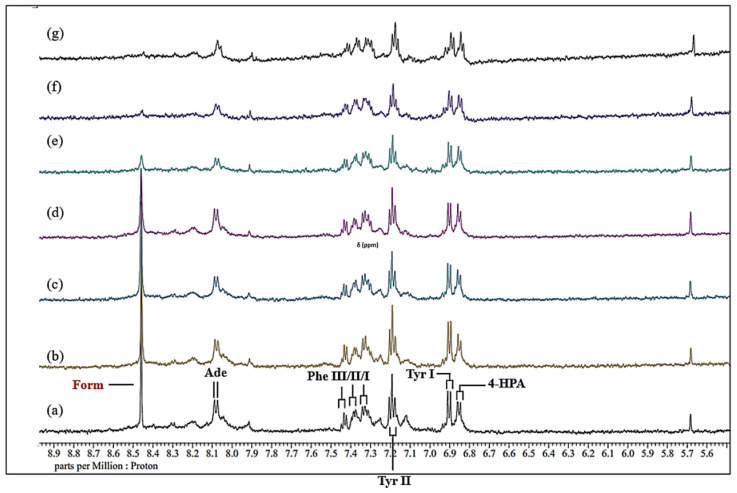

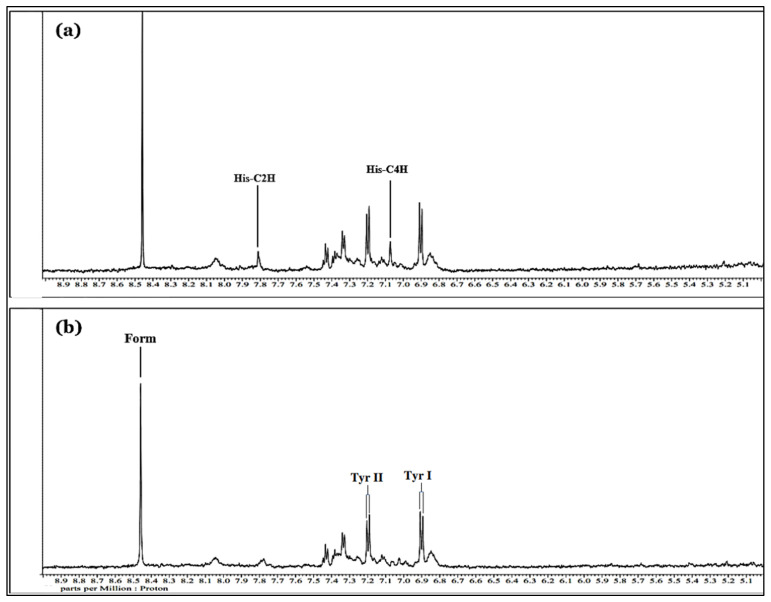
(**Top**)**:** Partial (5.50–8.98 ppm regions) 600.17 MHz ^1^H NMR spectra of (**a**) a control human WMSS specimen and the same sample following the addition of (**b**) 71 µmol/L, (**c**) 140 µmol/L, (**d**) 280 µmol/L, (**e**) 410 µmol/L, (**f**) 540 µmol/L, and (**g**) 670 µmol/L of Ni(II). (**Bottom**)**:** Expanded 5.00–9.00 ppm regions of a further WMSS sample spectrum (**a**) prior to and (**b**) subsequent to the addition of 71 µmol/L Ni(II), showing the complete Ni(II)-mediated removal of the histidine imidazole ring proton resonances. Typical spectra are shown. Abbreviations: Ade, adenine-C3H and -C8H singlets; Form, formate-H; His-C2H and -C4H, histidine imidazole ring-C2H and -C4H proton resonances, respectively; 4-HPA, 4-hydroxyphenylacetate-C3H/C5H aromatic ring protons; Phe I, II, and III, phenylalanine-C2H/C6H, C4H, and C3H/C5H aromatic ring protons, respectively; Tyr I and II, tyrosine-C3H/C5H and C2H/C6H aromatic ring protons, respectively. The key Ni(II) ion WMSS ligand formate is labelled in red. In the top series of spectra, the 4-hydroxyphenylacetate-C2H/C6H doublet resonance (δ = 7.16 ppm) overlaps significantly with that of the corresponding signal of tyrosine, so the superimposed signal appears to be a triplet. In the top WMSS spectral profiles, the histidine imidazole ring proton resonances were not clearly detectable.

As previously observed [13,17], many prominent, sharp signals assignable to a wide range of low-molecular-mass biomolecules can be observed in the 1D 600 MHz ^1^H NMR profiles of control (untreated) human WMSS samples (Figure 1 and Figure 2). The high- and low-field regions (0.00–4.28 and 5.50–8.98 ppm, respectively) for a typical WMSS spectrum acquired are shown in Figure 1a and Figure 2a, respectively, and a total of 26 salivary biomolecules were assigned and identified using this technique. In particular, signals arising from short-chain organic acid anions such as acetate, formate, lactate, propionate, pyruvate, 5-aminovalerate, and succinate were observed in conjunction with those of amino acids such as glycine, alanine, valine, histidine, phenylalanine, and tyrosine and carbohydrates, including the molecularly mobile carbohydrate side chains of acute-phase glycoproteins, the latter being known as the GlycA signal [32]. Such resonances were observable throughout the entire (0.70–9.00 ppm) spectral range.

Although all study participants were strictly instructed not to consume alcoholic beverages during the 24 h period prior to sample collection, further ‘in-house’ studies have revealed that this period may not be sufficient to ensure the exclusion of dietary sources of this agent completely from all WMSS samples analysed, so therefore it remains a possibility that traces of ethanol may still be derived from its dietary consumption prior to the 8 h cut-off threshold instituted.

In the aromatic regions of the spectra acquired (δ = 6.80–8.00 ppm), distinctly visible broad signals centred at ca. 6.90, 7.12, 7.25, and 7.92 ppm (Δ*v*_1/2_ ≥ 20 Hz) were perceptible in many of the spectra acquired. These resonances are conceivably attributable to the aromatic ring protons of tyrosine, phenylalanine, histidine, and/or tryptophan residues present in one or more WMSS proteins, their enhanced line widths reflecting the diminished molecular mobility and hence shorter T_2_ values of such biomacromolecules. Further broad macromolecule signals were also visible at δ = 8.05 and 8.21 ppm.

### 3.2. Two-Dimensional Homonuclear ^1^H-^1^H COSY Analysis of WMSS Samples

**Figure 3:**
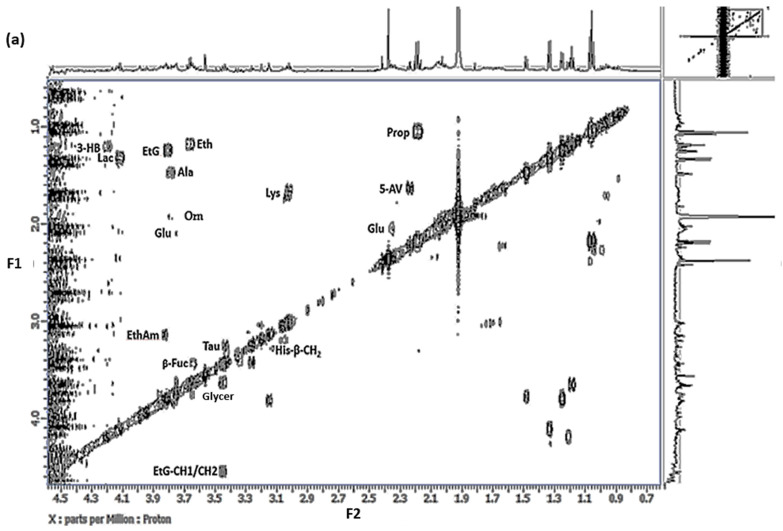

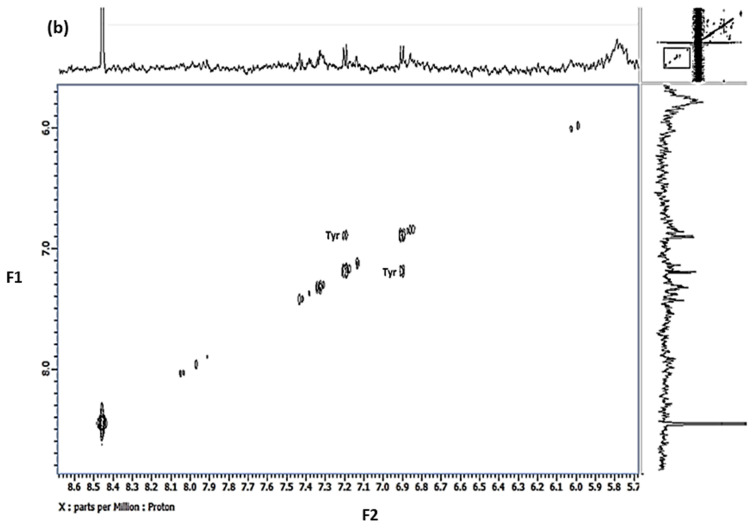
(**a**,**b**) Partial 0.62–4.60 and 5.70–8.70 ppm regions of 600 MHz ^1^H-^1^H COSY NMR spectra of a WMSS sample, respectively. Typical spectra are shown. Connected resonances shown are those for alanine-CH_3_/-CH (Ala); 5-aminovalerate-α-CH_2_/β- and γ-CH_2_ (5-AV); ethanol-CH_3_/-CH_2_OH (Eth); ethanolamine-CH_2_NH_2_/-CH_2_OH (EthAm); ethyl glucuronate-CH_3_/-CH_2_ signals (EtG); ethyl glucuronate ring-C1H/C2H protons (EtG-CH1/CH2); β-fucose ring protons (β-Fuc); glutamate side-chain protons, β-CH_2_/γ-CH_2_ (Glu); glycerol-CH_2_OH/-CHOH (Glycer); histidine-β-CH_2_ protons (His); 3-D-hydroxybutyrate-CH_3_/>CHOH (3-HB); lactate-CH_3_/-CHOH protons (Lac); amino acid lysine side-chain protons (Lys); ornithine amino acid side-chain protons (Orn); propionate-CH_3_/-CH_2_ (Prop); taurine-CH_2_NH_2_/-CH_2_SO_3_^−^ (Tau); and tyrosine- C2H/C6H and -C3H/C5H aromatic ring protons (Tyr).

Figure 3 shows the 0.66–4.58 and 5.68–8.68 ppm regions of a typical 2D ^1^H-^1^H COSY spectrum acquired on a WMSS sample to confirm the ^1^H NMR assignments made. These profiles clearly reveal confirmatory resonance connectivities for lactate (δ = 1.33 (*d*) and 4.13 ppm (*q*)), propionate (δ = 1.05 (*t*) and 2.17 ppm (*q*)), 5-aminovalerate (δ = 1.64 (*m*), 2.23 (*t*), and 3.02 ppm (*t*)), and 3-D-hydroxybutyrate (δ = 1.23 (*d*) and 4.16 ppm (*m*)); the amino acids alanine (δ = 1.48 (*d*) and 3.78 ppm (*m*)), glutamate (δ = 2.05 (*m*), 2.13 (*m*), and 2.35 (*dt*) ppm), histidine (δ = 3.15 (*m*) and 3.22 ppm (*m*) β-CH_2_ resonances), taurine (δ = 3.24 (*t*) and 3.43 ppm (*t*)), and tyrosine (δ = 6.89 (*d*) and 7.23 ppm (*d*)); ethanolamine (δ = 3.13 (*t*) and 3.80 ppm (*t*)); and the L-carbohydrate β-fucose (coupled-C2H (*dd*) and -C3H (*dd*) resonances located at δ = 3.45 and 3.64 ppm, respectively). Also visible were glycerol (δ = 3.54/3.65 and 3.77 ppm) (-CH_2_OH and -CHOH, respectively (ABX coupling pattern)) and ethanol (δ = 1.17 (*t*) and 3.66 ppm (*q*)) resonances, small levels of the latter being retained in WMSS samples, as noted above. Moreover, uniquely, the ethanol metabolite ethyl glucuronide was also detectable in such COSY spectra (δ = 1.24 (*t*) and 3.73 (*q*) ppm together with glucuronyl residue -C1H and -C2H multiplets located at δ = 4.51 and 3.48 ppm, respectively [33]), an agent which would not normally be readily distinguishable from a visual inspection of the 1D spectra acquired on this biofluid. Ethyl glucuronide’s resonance patterns are clearly distinguishable from that of the further ethanol metabolite ethyl glucoside in the ^1^H-^1^H COSY spectra [33]. To the best of the authors’ knowledge, this is the first time that this ethanol metabolite has been detected in human saliva by ^1^H NMR analysis. Therefore, as expected, this 2D NMR technique was highly informative for seeking and confirming the identities of the molecular chains present in salivary biomolecules, a development permitting the unambiguous assignment of their ^1^H signals. Indeed, 2D ^1^H-^1^H COSY NMR analysis served to identify a further seven WMSS metabolites over those identifiable with only the 1D ^1^H NMR approach, including ornithine, ethanolamine, and ethyl glucuronide. Additionally, it confirmed the identities of more than ten of the coupled proton resonance-containing biomolecules primarily detected using the 1D ^1^H NMR analysis strategy, for example lactate, propionate, and alanine, amongst others.

### 3.3. ^1^H NMR Analysis of Pooled WMSS Admixture Samples for QC Purposes

With the exception of adenine and threonine, the ^1^H NMR spectra acquired on these repeatedly analysed QC samples were found to contain all metabolite resonances detectable in all of the profiles of the n = 12 cohort of (untreated in vitro) healthy human WMSS samples analysed in this study (Figure S4.1 in Section S4). These QC samples were randomly inserted on the NMR autochanger device during the routine triplicate acquisition of the ^1^H NMR profiles of individual WMSS samples (n = 63 in total) in order to condition the analytical platform, perform intra-study reproducibility measurements (QC), and also to correct mathematically for any systematic errors.

It should also be noted that the PQN row-wise normalisation treatment employed [30] prior to full data analysis effectively provided a full median-based spectrum for comparative normalisation purposes, and hence, this precaution taken is similar to the generation of pooled WMSS samples described here.

### 3.4. Spectral Titrations of WMSS Samples with Increasing Added Concentrations of Ni(II)_(aq.)_

Routine visual inspection of all ^1^H NMR profiles of WMSS samples acquired showed that with increasing sequential levels of added paramagnetic Ni(II)_(aq.)_ ions, the selected biomolecule resonances therein demonstrated concentration-dependent increases in the line widths of their resonances. Indeed, the high- and low-field regions of the spectra shown in Figure 1 and Figure 2, respectively, show an example of a spectral titration involving the stepwise addition of final Ni(II) concentrations of 71 to 670 µmol/L to a typical WMSS sample. Corresponding spectral titrations of the same or alternative typical donor WMSS samples with Ni(II) ion are shown in the expanded regions of such spectra (Section S5, Figures S5.1–S5.3). Clearly visible line-broadening effects were observed for the salivary succinate (δ = 2.41 ppm (*s*)) and histidine imidazole ring resonances (δ = 7.07 (*s*) and 7.84 ppm (*s*)) along with those of ethanol (δ = 1.18 (*t*) and 3.66 (*q*) in some WMSS samples in which it was present) at the lowest added concentration of Ni(II). Broadenings to lactate’s two signals (δ = 1.33 (*d*) and 4.13 ppm (*q*)) were also visible. Hence, these data provided evidence for the importance of these salivary biomolecules to complex this metal ion when dietarily ingested, or, alternatively, when liberated from dental prostheses containing significant contents of this metal ion *in vivo*.

Furthermore, enhancement of the added Ni(II) ion level to a level of 140 µmol/L gave rise to significant broadenings of the resonances attributable to formate (δ = 8.46 ppm (*s*)) and pyruvate (δ = 2.39 ppm (*s*)). Indeed, the formation of formato- and pyruvato-nickel(II) complexes, respectively, is responsible for the observed broadening of these ^1^H NMR signals, and such data also provide evidence for the complexometric consumption of Ni(II)_(aq.)_ by these ligands/chelators, the first of these presumably as [Ni^II^(HCO_2_)]^+^ and higher complex species [34]. Further increases in the levels of added Ni(II) ion to 280 and 410 µmol/L gave rise to metal ion-mediated broadenings of the ^1^H NMR resonances of glycine (δ = 3.56 ppm, (*s*)), acetate (δ = 1.92 ppm (*s*)), and N-acetylneuraminate (δ = 2.06 ppm (*s*)), all at 280 µmol/L, along with those broadened at the 410 µmol/L added Ni(II) level, including alanine (δ = 1.48 ppm, (*d*)) and 5-aminovalerate (δ = 2.23 ppm (*t*)) in the high-field region of the spectra obtained. These, along with the propionate signals, which become broadened at the 280 µmol/L Ni(II) concentration, may therefore also be classified as significant Ni(II) complexants. Also visible were Ni(II)-induced broadening effects on WMSS 3-aminoisobutyrate, 3-D-hydroxybutyrate, and adenine, which were observable at the 410 µmol/L added Ni(II) level. Moreover, the resonances found to significantly broaden at added Ni(II) concentrations of ≥ 540 µmol/L were those arising from choline, γ-aminobutyrate, methylamine species, phenylalanine, and tyrosine.

With spectro-visual examination, it was also clear that at the highest levels of added Ni(II) ion (410–670 µmol/L), the WMSS signals arising from biomolecules with the most Ni(II)-complexing power became virtually indistinguishable in the acquired spectra (Figure 1 and Figure 2). Furthermore, it was found that all resonances influenced by the lower added levels of Ni(II) demonstrated further increases in their line-width values as the concentration of added Ni(II) increased further. Additionally, the intermediate level Ni(II) ion-induced broadenings of the N-acetyl sugar acetamido-CH_3_ group series of resonances observed also provided evidence for the involvement of these species in the complexation/chelation of this transition metal ion in human saliva. Certainly, it appears that these agents may be participating in this interaction with Ni(II) as free sugars and/or low-molecular-mass oligosaccharides and also as residues present in N-acetylated glycoprotein carbohydrate side chains (visible as the broad salivary GlycA resonance). Table 1 below summarises which metabolites were most visually impacted by the addition of Ni(II) and at which concentrations these changes were first visibly observed in the WMSS spectra acquired.

However, as recommended by Strathmann and Myneni [35], each WMSS sample was reacted for a minimum period of time (2–3 h in this case) prior to ^1^H NMR analysis in order to ensure that complexation equilibria were achieved in these biofluid solutions. This was considered relevant since Ni(II)-water exchange rates are ca. 10^4^ s^−1^ [36]. In order to assess these potential time-dependent effects, an ANOVA model which considered the factors ‘between-added Ni(II) concentrations’ and ‘between-participants’ as fixed effects, and ‘between-replicates’ as a random effect (the latter the case without a significant deviation from the null hypothesis of no between-replicate, albeit analysis time-dependent, differences being observed) was employed. This model was applied to all 85 ^1^H NMR buckets at all added Ni(II) levels for all 12 participants. Although both these main fixed effects displayed very highly significant *p* values for an extensive array of these buckets, this replicative ‘random’ effect was not found to be statistically significant (i.e., uncorrected *p* > 0.05) in any of them, so this model’s validity was upheld. For example, 95% CIs for triplicate sample random effects coefficients found for the -CH_3_ group buckets of WMSS biomolecules were found to be −0.01 to 0.09, −0.06 to 0.04, and −0.08 to 0.02 for replicate 1, 2, and 3 determinations (with increasing consecutive analysis times), respectively, made on propionate; −0.03 to 0.14, −0.07 to 0.10, and −0.15 to 0.01, respectively, for those made on 3-D-hydroxybutyrate; and −0.01 to 0.05, −0.04 to 0.02, and −0.04 to 0.02, respectively, for those made on lactate; i.e., all were not significantly different from zero, nor indeed were all three of these estimates for all other buckets tested in this manner at all added Ni(II) levels and for all participants. Hence, the ^1^H NMR profiles generated from the replicated Ni(II)-WMSS admixtures were independent of the equilibration time periods evaluated at 25 °C, and hence, a minimum analysis time of ca. 2.0 h was found to be sufficient for reactions to proceed to completion. Moreover, suitable chemical model system experiments featuring the reactions of Ni(II) with both amino acid (e.g., histidine and alanine) and carboxylic acid anion ligands (e.g., lactate and formate) confirmed that these reactions were complete within the mixing time at this temperature.

From this spectro-visual inspection, which included consideration of all spectral regions (Figure 1 and Figure 2, and those shown in Sections S4 and S5 of the Supplementary Information), the order of complexation of Ni(II) by salivary biomolecules was found to be lactate ≈ succinate ≈ histidine ≈ ethanol > formate ≈ pyruvate > glycine ≈ propionate ≈ acetate ≈ 5-aminovalerate > alanine ≈ 3-aminoisobutyrate ≈ 3-D-hydroxybutyrate ≈ adenine > choline ≈ GABA ≈ methylamines ≈ tyrosine ≈ phenylalanine. However, it is important to note that many Ni(II) ion-induced changes were not clearly visible from direct visual examination of the ^1^H NMR profiles alone, and therefore, MV metabolomics analysis was performed in order to elucidate these spectral modifications. The strategies employed for this analysis are described in Section 3.6 below.

Clearly, the expanded spectral region shown in Figure S5.3 verifies that added Ni(II) contributes towards a substantial broadening of the lactate-CH resonance. As observed for this metabolite’s -CH_3_ group signal (Figure 1), its intensity is significantly reduced after the addition of 280 µmol/L of Ni(II), and it is virtually completely depleted at an added level of 670 µmol/L. Similarly, significant intensity reductions were also observed for the glycolate-, creatine-, and phosphocreatine-CH_2_ signals at added Ni(II) levels of 280–540 µmol/L. The latter phosphocreatine resonance also undergoes an upfield shift of ca. 10 Hz at the added Ni(II) level of 280 µmol/L, and more so at higher concentrations, as do the creatine, glycolate, and 3-D-hydroxybutyrate signals, albeit to a lesser extent.

### 3.5. Influence of Added Ni(II) Ions on the Intensities and Line Widths of Key Biomolecular Salivary Complexants

Figure S6.1(a) shows mean ± SD values of the line width at half-height (Δ*v*_1/2_) parameters of the lactate-CH_3_, acetate-CH_3_, and formate-H resonances for n = 7 separate donor WMSS samples investigated. These data indicate that despite some similarities between this Δ*v*_1/2_ parameter for all three metabolite signals up to an added Ni(II) concentration of 280 µmol/L, at higher levels, the lactate-CH_3_ doublet signal was broadened more than the formate-H singlet, which, in turn, was broadened more than that of the acetate-CH_3_ group (singlet also). Similarly, Figure S6.1(b) shows added Ni(II) ion concentration-dependent decreases in the TSP-normalised integrated intensity of the δ = 8.44–8.48 ppm formate singlet resonance bucket as mean ± 95% confidence intervals (CIs) for all n = 12 different Ni(II)-titrated WMSS samples along with corresponding intensity modifications observed in its adjacent resonance buckets, specifically, the δ = 8.40–8.44 and 8.48–8.52 ppm ones. These data provided evidence that the Ni(II)-induced broadening effect on this resonance was limited to the 8.44–8.48 ppm bucket only, and this was predictable from the maximal increase in its Δ*v*_1/2_ value of ca. 9 Hz (equivalent to 0.015 ppm at an operating frequency of 600 MHz, which is less than half the bucket width of 0.040 ppm) observed at an added level of 470 µmol/L. Further information on these experimental data is provided in Section S6.

This effect on formate was further ratified via an examination of any bucket superimposition for this formate signal, and this verified that all such Ni(II)-induced broadenings of it were exclusively confined to this bucket alone, without any visible superimposition on neighbouring ones at added concentrations of 270 µmol/L or higher (Figure S6.1(c)). The overall mean intensity of this resonance decreased by a value of as much as 94%, and this indicated that it had been substantially paramagnetically shifted via the influence of the Ni(II) metal ion centre in its complex(es) in aqueous solution.

Also shown in Figure S6.1 is a plot of the chemical shift (δ) values of the lactate-CH_3_ and -CH versus the added Ni(II) concentration in typical WMSS samples (Figure S6.1(d)). Clearly, for this metabolite there was little or no effect of this metal ion on both of its proton resonance δ values (n = 3 different samples were examined).

### 3.6. ^1^H NMR-Linked Metabolomics Tracking of Significant Ni(II)-Complexing Biomolecules in the WMSS Sample Cohort

#### 3.6.1. Violin Plots of the Intensity of Key Added Ni(II)-Influenced Resonances Arising from Application of a Single-Factor One-Way ANOVA Model (Model 1)

Violin plots for both the raw and normalised datasets observed for ^1^H NMR signals assigned to leucine/isoleucine, lactate (x 2), acetate, succinate, and formate are shown in Figure S7.1 of Section S7 (normalised data were PQN-normalised, glog-transformed, and Pareto-scaled). Each of these plots reveal clear patterned decreases in intensity with increasing added Ni(II) level, and hence, they belong to class 1 of the added Ni(II) ion biomolecule responders, as described in Section S8 (Supplementary Information) and Section 3.6.3.

#### 3.6.2. Preliminary Application of Interactive PCA and PLS-DA Strategies

**Figure 4:**
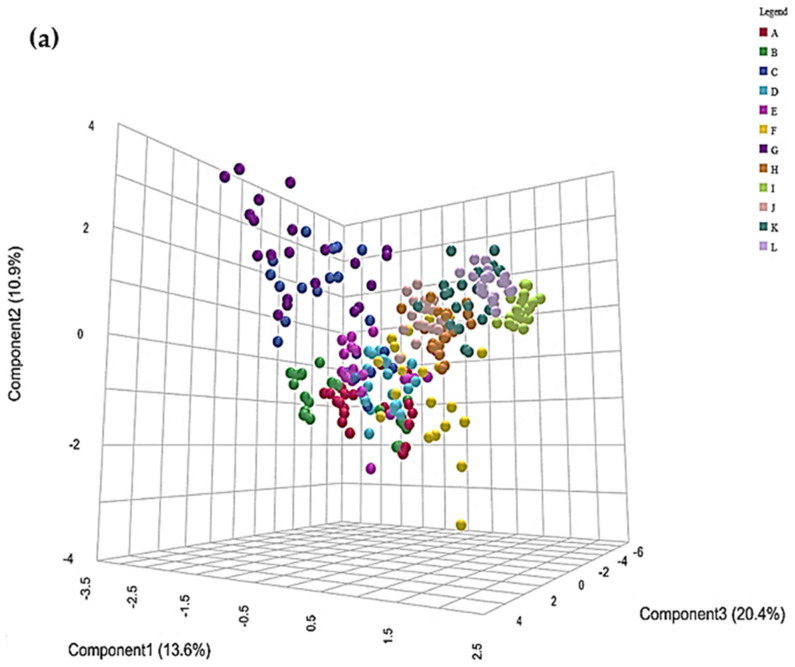

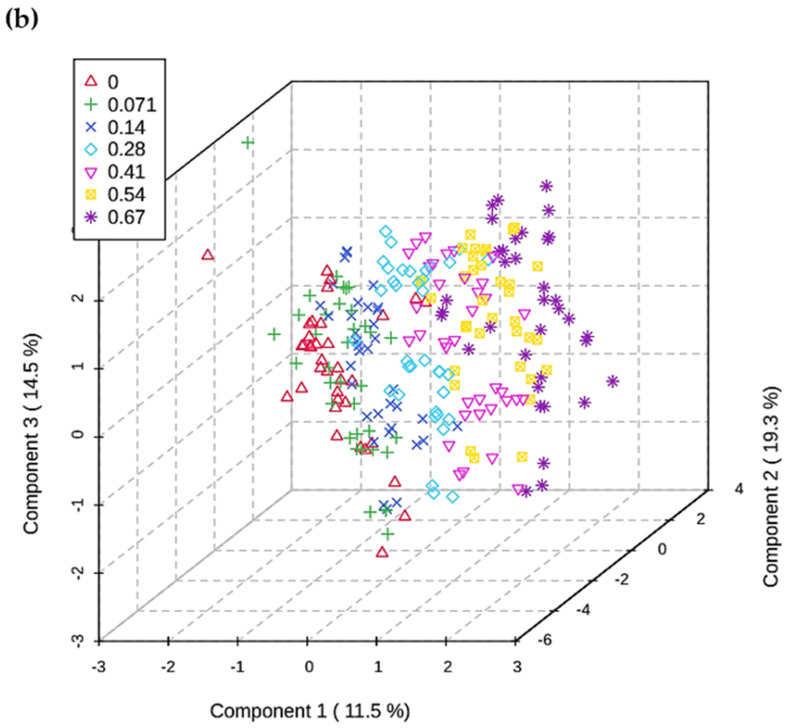
(**a**) Interactive 3D PC3 versus PC2 versus PC1 PCA scores plot showing different clusterings arising from the 12 different WMSS donor study participants. (**b**) Corresponding 3D PLS-DA scores plot showing distinctions between the seven different Ni(II) concentrations added to the WMSS samples (0–0.67 mmol/L). Datasets were constant sum- or probability quotient-normalised (CSN or PQN, (**a**) and (**b**), respectively), and then glog-transformed and Pareto-scaled prior to analysis. Colour coding for different participants (A–L) and increasing added Ni(II) levels (mmol/L) are shown in (**a**) and (**b**), respectively.

Figure 4a exhibits a 3D PC3 versus PC2 versus PC1 scores plot of the Ni(II) WMSS speciation dataset acquired. Interestingly, this plot provides strong evidence for distinctions between each of the study participants, with participants C and G being clearly distinct from the remainder through their higher PC2 scores, and also some clear distinctiveness between participants A, B, D, E, F, H, J, K, and L via combinations of their score values on both PCs 1 and 2. Moreover, corresponding paired PC2 versus PC1, PC3 versus PC1, and PC3 versus PC2 plots confirmed that the between-participants effect largely loaded on PCs 1 and 2, followed by PC3 to a lesser extent. Adjusted pairwise PCA PERMANOVA statistics demonstrated that only three of the participant comparisons were not significantly different from each other, and this confirms a high level of heterogeneity between the different sample donors recruited to the study. However, no clear added Ni(II) concentration-dependent patterns were easily discernible from this PCA 3D scores plot.

Nevertheless, clustering distinctions between the different added Ni(II) concentration groups were much more visible from a corresponding 3D PLS-DA scores plot, and these revealed a clear sequential added Ni(II) concentration dependence (Figure 4b). Indeed, in this plot, groups with the highest added Ni(II) levels (0.54 and 0.67 mmol/L) had higher scores for both components 1 and 2, whereas those with the lowest concentrations had lower component 1 and 2 scores. The third component contributed little to this clustering distinctiveness. This model was evaluated with a satisfactory cross-validating Q^2^ statistic value of >0.85 with a minimum of five components, and a permutation *p* value of <5.0 × 10*^−^*^4^.

Hence, these prior metabolomics analysis methods proved that it was clearly possible to distinguish between WMSS sample clusterings derived from differences between both sample donor participants and increasing added Ni(II) concentrations.

#### 3.6.3. Detailed Metabolomics Investigations of the Dependence of ^1^H NMR Profile Changes on Added Ni(II) Concentrations: Classification of Different Types of Ni(II)-Responsive Biomolecules

A series of four experiments was conducted in order to explore the sequential biomolecular ^1^H NMR bucket responses of participant WMSS samples to the added Ni(II) ion as a function of its added concentration. In this manner, it was possible to distinguish between different classes of Ni(II)-responsive salivary metabolites. Results from these experiments are summarised below, although full details are provided in Section S8.

**(1)** Firstly, a series of two-comparator PLS-DA models which evaluated differences between the ^1^H NMR profiles of WMSS samples obtained at each added Ni(II) level and that of the untreated zero control samples were performed. From these, VIPs and rank Ni(II) complexant orders were determined and reviewed. This approach was also used for the purpose of ascertaining WMSS complexants involved in the early stages of the Ni(II) ion titrations, most especially those at added levels of only 0.071 mmol/L.

From these dual comparisons made, the order of variable importance of the histidinate ligand was found to be 4th, 1st, 2nd, and only 15th at added Ni(II) levels of 0.071, 0.14, 0.28, and 0.41 mmol/L, respectively (as expected, VIPs for this biomolecule were <1 at 0.54 and 0.67 mmol/L added Ni(II) levels), and none of the carboxylate anion complexants found in class 1 specified below classified within the top 10 until an added Ni(II) level of ≥0.28 mmol/L was attained. Prior CSN followed by glog transformation and Pareto scaling was used for these analyses.

**(2)** Secondly, AHC analysis and AHC-facilitated heatmaps were constructed for the whole spectral range in order to segregate responding biomolecular WMSS complexants into different added Ni(II) ion concentration-dependent responding classes. A total of four different responding classes were isolated.

In view of the non-additive nature of this concentration-dependent response, a heatmap displaying colour-coded individual ^1^H NMR bucket responses to increasing added Ni(II) levels was constructed, and for this purpose, we elected to explore a dataset which was also primarily constant sum normalised (Figure S8.1). This figure revealed that the spectral modifications found belonged to one of four different classes (classes 1–4), which were characterised by the performance of a supporting AHC analysis of the chemical shift bucket predictor variables for Ni(II) response. These details and accompanying figures are provided in Section S8 of the Supplementary Information. In summary, two of these major clusters were class 1 (carboxylate anion O-donor ligands which showed the greatest magnitude response throughout the whole spectral titration range) and class 4 (seven biomolecules of low salivary levels, i.e., free amino acids such as taurine, histidine, and phenylalanine, and also ethanolamine and cholines), which all significantly decreased in intensity within the 0.00–0.14 mmol/L added Ni(II) concentration span. Full descriptions of all of these four major AHCs and added Ni(II)-responsive clusters, along with associated plots, are also available in Section S8.

**(3)** Thirdly, paired sample t-tests, which eliminate interfering effects arising from the between-participants component of variance, were employed to explore the sequential concentration-dependent effects of added Ni(II) ions.

Again, these were two group comparator models, with only single Ni(II) level classifications being compared against the untreated control group, this time using the PQN row-wise normalisation strategy prior to glog transformation and Pareto scaling. Corresponding *p* values were estimated for each added Ni(II) concentration, and the Bonferroni correction for multiple comparisons was applied; these are also provided in Section S8. This analysis found that the lower levels of added Ni(II) were found to be largely populated by non-carboxylato donor ligands, including histidine, taurine, ethanolamine, dimethylamine, and the amino acid glutamate, whereas at higher added concentrations, lactate, succinate, formate, and acetate became increasingly dominant, although it should be noted that the β-amino acid taurine still featured as the top Ni(II) complexant at an added level of 0.67 mmol./L Ni(II).

**(4)** Finally, since at an added level of only 0.071 mmol/L Ni(II) there was not a statistically significant increase observed in the Δ*v*_1/2_ value of the TSP internal standard ^1^H NMR resonance (more specifically, little or no complexation of this metal ion by it), as shown in Figure S3.1, a TSP-normalised (i.e., purely quantitative) dataset for this Ni(II)-treated group only was obtained, and following glog transformation and Pareto scaling, made a final comparison of its resonance intensities with that of a correspondingly TSP-normalised and transformed untreated control group (an approach also involving multiple paired sample *t*-tests).

Table S2 provides a list of the top 20 metabolites with the Bonferroni-corrected statistical significance of modifications in their intensities, which all comprised Ni(II)-induced decreases for these experiments. These results again show that with the exception of lactate, ‘early’ low-level added Ni(II) WMSS complexants were not the organic acid anion species found to predominate its complexation at higher added concentrations of this metal ion. Indeed, amino acids such as histidine, taurine, proline, phenylalanine, tyrosine, and glutamate are strongly featured along with other nitrogenous or alcohol-based donor functions present in other metabolites, including methylamines, creatine, creatinine, choline, and 5-aminovalerate.

#### 3.6.4. Application of Covariate- and Interaction-Effect Balancing Two-Way ANOVA Models (Direct Univariate ANOVA-Based Analysis)

This approach was employed in order to adjust the bioanalytical model for any differences found between the n = 12 different sample donors participating in the study, along with the possible ‘between-added Ni(II) concentrations x between-participants’ first-order interaction effect, whilst ascertaining differences between the main source of the variations of interest, specifically those between increasing added Ni(II) levels, for all 85 fixed buckets investigated (Section S9, Supplementary Information). Since each bucket variable was analysed in a univariate ANOVA context only, allowances for differential relationships existing between their mean biomolecule resonance intensity responses and added Ni(II) concentrations (i.e., differential type class 1–4 responses, as outlined above) did not have to be accounted for.

This full ANOVA-based analysis (Table S3) clearly demonstrated that the experimental design-mediated removal of the confounding between-participants random and ‘between-added Ni(II) concentrations x between-participants’ interaction effects sharpened the significance of differences found for the ‘between-added Ni(II) concentrations’ main factor, with Bonferroni-corrected *p* values decreasing from <10^−8^ to <10^−65^ to <10^−86^ for the lactate-CH_3_ signal for the model 1 to model 2 to model 3 analyses, respectively; from <10^−23^ to <10^−72^ to <10^−77^, respectively, for the acetate-CH_3_ resonance; from <10^−27^ to <10^−41^ to <10^−75^, respectively, for the formate-H signal; and not significant (ns) to <10^−13^ to <10^−65^, respectively, for ethanolamine’s -CH_2_OH resonance. Therefore, such careful planning of experimental designs, with a full consideration of all contributory variance components for this class of metabolomics experiments, offers major precision-boosting advantages.

Interestingly, virtually all buckets tested had a statistically significant between-participants random effect, and those with extremely high F variance ratios > 200 were those for ethanol > propionate > lactate > ethanolamine. This revealed that there was a very wide variance (scatter) of these values between participants indeed, and this undoubtedly reflects differential levels of Ni(II)-mediated WMSS sample responses, complexation reaction or otherwise, for each of the participants recruited to the current study. Additionally, the high-level statistical significance of the first-order interaction effect for many salivary metabolites, including those for formate, propionate, lactate, and tyrosine, demonstrates that the patterns of their responses towards increasing added Ni(II) concentrations were certainly not uniform for each participant involved, and therefore, this may act as a measure of their salivary response heterogeneities, which may represent a valuable ‘scatter’ measure for many future metabolomics studies, e.g., those monitoring the dosage responses of drugs, etc.

#### 3.6.5. ASCA Analysis of the Metabolomics Dataset

Additionally, we fitted an ASCA model to the dataset with ‘between-added Ni(II) levels’ and ‘between-participants’ as the main factors for consideration, together with their first-order interaction variance contribution. Results obtained from permutation tests (Figure S10.1, Section S10) provided strong evidence that a model with only the two main factors, and not also including the first-order ‘between-added Ni(II) concentrations x between-participants’ interaction effect (CP*_ij_*), was satisfactory to describe the MV ASCA dataset (*p* =< 0.005, <0.005, and 0.76, respectively). From these test values, it is clear that the between-participants effect accounted for a similar proportion of the total variation observed to that of the added Ni(II) concentration in this model. From their threshold leverage and squared prediction errors, key discriminatory metabolites found for the added Ni(II) ion effect were the formate and both lactate-CH_3_ and -CH signals (complete dataset only).

### 3.7. ^1^H NMR-Based Metabolomics Tracking of Significant Ni(II)-Complexing Biomolecules in the Pooled WMSS QC Sample

As noted in Section 3.3, the biomolecular composition of the pooled WMSS sample prepared for QC purposes was found to contain all resonances detectable in the individual samples, bar those arising from adenine and tryptophan. As expected, the ^1^H NMR spectral titration of triplicate aliquots of this sample with the same concentrations of added Ni(II) used in the main focus of the study relayed above also gave rise to major influences on the intensities of a wide range of potential WMSS Ni(II) complexants, i.e., predominantly paramagnetically induced broadenings. Indeed, one-way ANOVA analysis revealed that there were very highly significant ‘between-added Ni(II) level’ differences between mean WMSS biomolecule concentrations, and the results acquired were similar to those described in Section 3.4, Section 3.5 and Section 3.6, with formate, ethanol, succinate, acetate, propionate, ethanolamine, and lactate being the most important, in that order (Table S4, Section S11). Moreover, Table S4 shows the results acquired from the application of the PLS-DA strategy to the analysis of this pooled sample exposed to increasing concentrations of added Ni(II), and this generated a rank order of biomolecular complexants for this metal ion which was determined by their VIP values, the most important being formate > lactate > succinate > acetate > tyrosine > phenylalanine > ethanol.

### 3.8. Reversal of Ni(II)-Induced ^1^H NMR Spectroscopic Modifications with Added EDTA

**Figure 5:**
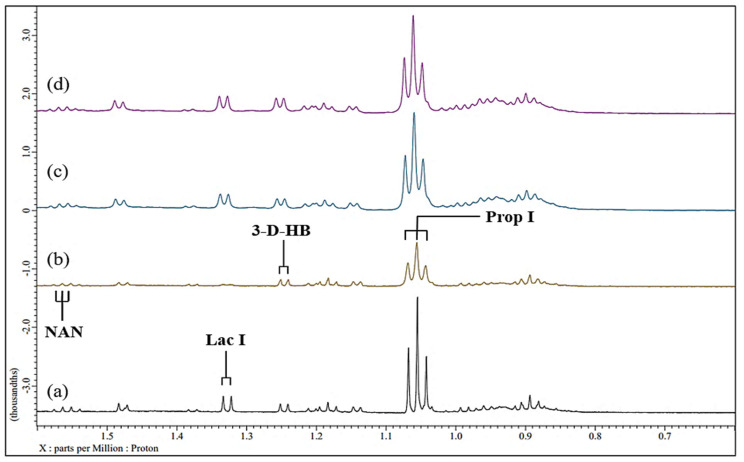

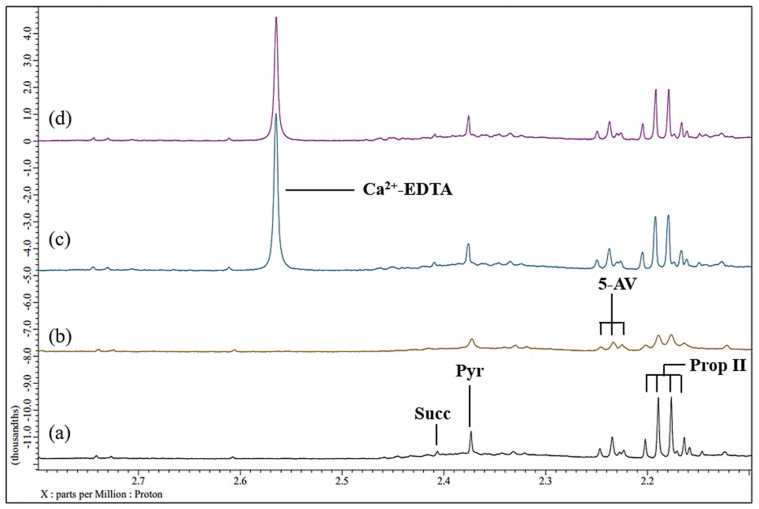

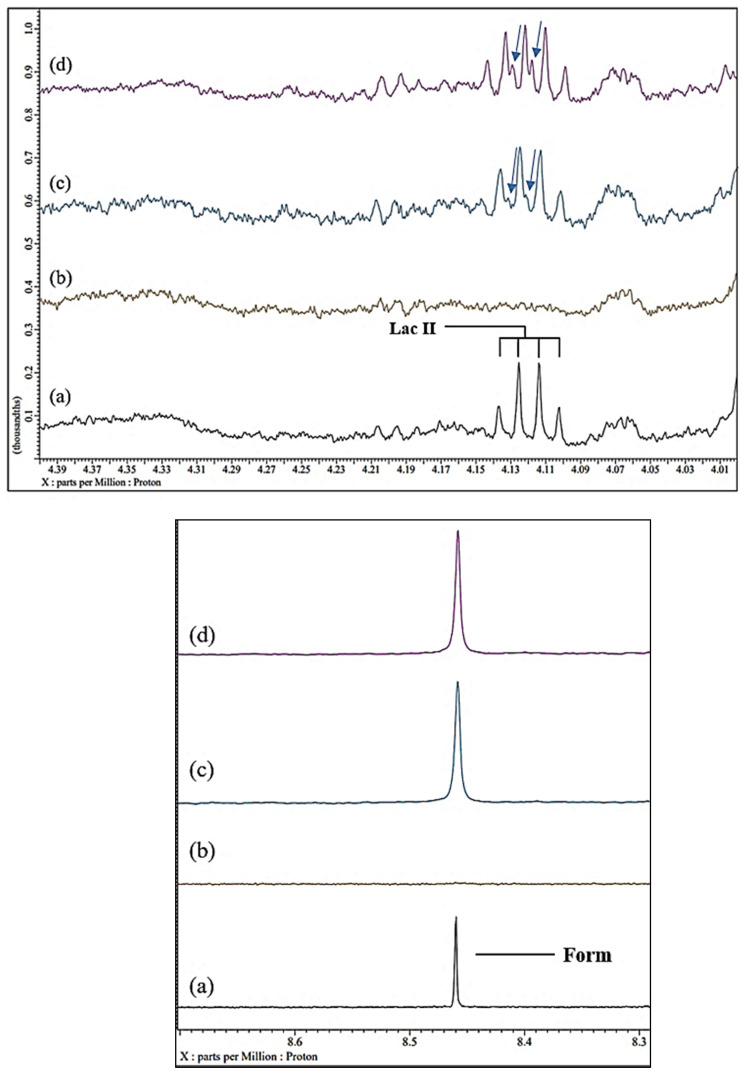
Influence of added EDTA on the ^1^H NMR profiles of Ni(II)-loaded WMSS samples. Partial (expanded) 0.60–1.60 (**top**), 2.10–2.80 (**second from top**), 4.00–4.40 ppm (**third from top**), and 8.00–8.70 ppm (**bottom**) regions of 600 MHz ^1^H NMR spectra of (**a**,**b**), a control healthy human WMSS specimen before and after the addition of 670 µmol/L Ni(II), respectively. (**c**,**d**) Same as (**b**), but subsequently treated with 770 µmol/L EDTA and equilibrated at 37 °C for periods of 30 min and 24 h, respectively. Typical spectra are shown. Abbreviations: same as Figure 1 and Figure 2. The blue arrows in the partial spectra (**c**,**d**) of the third from top diagram indicate new superimposing signals in the lactate-CH resonance region. The *n*-butyrate-CH_3_ and β-CH_2_ signals are those located at δ = 0.89 (*t*) and 1.56 ppm (*m*).

In principle, the Ni(II)-induced spectroscopic changes observed above should be reversible, a process which is promotable via the addition of a chelator that possesses a powerful Ni(II)-chelating ability, for example, one with a high stoichiometric, albeit biofluid pH- and ionic strength-conditional, thermodynamic equilibrium constant (*K*_eq_). This was investigated by the addition of an excess amount of EDTA (770 µmol/L) to WMSS samples containing added Ni(II) ions (670 µmol/L). Predictably, resonances for propionate, lactate, and formate, which were previously substantially broadened by added Ni(II), reappeared in the spectra acquired on the WMSS samples within 30 min after EDTA addition (Figure 5). However, following the 24 h equilibration period, further, albeit minor, changes were visible in the ^1^H NMR spectra, most notably that of the lactate-CH resonance, which revealed a further set of multiplet resonances in parts (c) and (d) of the third series of partial spectra shown in Figure 5. Indeed, there appears to be a time dependence of this spectral modification, with the signal(s) developing at 30 min post-EDTA addition and increasing in intensity within 24 h. This/these signal(s) may arise from an alternative WMSS biomolecule with ^1^H resonances superimposed on the lactate-CH proton quartet, although their resolution from the lactate-CH signal may be mediated by small changes in pH value following EDTA addition. However, no change in chemical shift value was found for this resonance at the 30 min post-EDTA addition time point. Therefore, this observation may also be consistent with the development and ^1^H NMR visualisation of a class of lactato-Ni(II) complex which is exchanging slowly on the NMR timescale, and which may also be diamagnetic under these experimental conditions. Notably, the polymeric or polynuclear nature of the selected lactato-Ni(II) complexes may also contribute towards these effects. Further investigations to explore this are currently in progress.

Despite the re-emergence of most of the Ni(II)-broadened ^1^H NMR resonances of complex-forming salivary biomolecules, it appears that these regenerated signals retain at least some of the broadening effect (Figure 5). Hence, this observation may be reflective of the retention of a small level of Ni(II) by the ligands giving rise to them under our experimental conditions. Indeed, an added EDTA level of 770 µmol/L as in our experiments may not be sufficient to remove all complexed or chelated Ni(II) from its most important WMSS ligands, and this is at least partially expected from the known salivary Ca^2+^ and Mg^2+^ levels, metal ions which also form stable complexes with the EDTA chelator. Consistent with this, also visible in these spectra are those of the ethylenic proton singlets of EDTA itself and its Ca^2+^-complex (δ = 3.22 and 2.57 ppm, respectively), the latter of which exchanges with the free chelator slowly on the ^1^H NMR timescale [37]. Also visible in some, but not all, spectra acquired on these EDTA-treated samples was the ethylenic proton singlet of the Mg^2+^-EDTA complex (δ = 2.76 ppm), but for the spectra displayed in Figure 5, this resonance had a very weak intensity when compared with that of the Ca^2+^-EDTA complex, and this confirmed the very low Mg^2+^ ion concentration of this sample when expressed relative to that of Ca^2+^. The additional, molecular symmetry-interrupting acetate proton resonance of the Ca^2+^-EDTA complex located in the δ = 3.06–3.17 ppm region (AB coupling pattern), was also readily visible in spectra acquired on the EDTA-treated WMSS samples.

## 4. Discussion

Heterogeneities in the molecular nature of transition and other metal ions in biological systems are of critical importance, and each complex species so formed may serve as an important mediator of its pathway-linked biological activities. Notably, in human and animal biofluids and tissues or other biosystems, some trace metal ions will be present as ‘free’ or aquo-/hydroxo-substituted mononuclear ion complexes, others as low-molecular-mass complexes with physiologically available ligands such as amine-NH_2_, carboxylate anion O-donors, and amino acids with both classes of donor atoms, which may be mono- or multinuclear, and also those which are reversibly or irreversibly bound to proteins or other biomacromolecules. Therefore, both careful and cautious approaches should be employed in order to perform state-of-the-art speciation tracking studies, which prior to the advent and advance of high-resolution NMR analysis for such investigations, often involved laborious and time-consuming separation of the metal ion complexes generated, processes providing opportunities for adverse trace element contamination. The concentrations of the metal ions concerned in the biosamples evaluated should also be critically considered, most especially since the distributions of such biomolecular complexes generated will be markedly dependent on the amounts of the complexants present or available. For example, in the current study, it appears that formate and lactate do not effectively compete for any available Ni(II) until its added level exceeds 71–140 µmol/L, but this range far exceeds estimates of that available in human saliva, which is ca. 75 nmol/L [21]. These experimental protocol complexities should also address further problems anticipated, including difficulties, extractive or otherwise, of the biofluid sample matrix compositions; the specificity of analyte analysis [38]; the establishment and maintenance of a suitable metal ion–biofluid ligand equilibration period for reaction completion, where required; and, where appropriate, prior separation and the avoidance of any metal ion–protein binding equilibria.

### 4.1. Thermodynamic Considerations of the Speciation Status and Added Concentration-Dependent Order of Ni(II)-Complex Formation in WMSSs

Results acquired from this study demonstrated that added Ni(II) ions were complexed by a series of ligands/chelators available in human WMSS samples. From our Bonferroni (FDR)-corrected values for covariate (between-participants and first-order interaction effect)-adjusted *p* values (Table S3), the overall ligand order was found to be formate > acetate > lactate > propionate > pyruvate > *n*-butyrate > leucine/isoleucine > alanine > glycine for the most powerful complexants/chelators available in the WMSS samples but at added concentrations of >140 or >280 µmol/L only. Such modifications to the ^1^H NMR profiles of WMSS samples were found to be readily reversible via the subsequent addition of EDTA (Figure 5), although the results acquired herein may also indicate the involvement of polymeric and/or polynuclear carboxylato-Ni(II) complexes which react more slowly with this chelator, with a depolymerisation process representing the rate-limiting step.

These salivary biomolecules may complex or chelate Ni(II), either individually or in concert (the latter including possible mixed-ligand ternary complexes), to generate species which may indeed play roles in determining the nature and scope of toxicity of this redox-active metal ion, which is known to be corrosively liberated from NiC-MADPs as implants. Moreover, some of the most important additional added Ni(II)-dependent resonance broadenings, which were also directly observable via computer-visual inspection of the spectral profiles, were those of succinate, ethanol, N-acetylneuraminate, and 5-aminovalerate, which were first observable at added Ni(II) levels of 71, 71, 280, and 410 µmol/L, respectively. Further biomolecules involved in Ni(II) complexation included histidine, ethanolamine, phenylalanine, creatine and phosphocreatine, choline, and dimethylamine, particularly histidine at the lowest added Ni(II) levels. Hence, the results obtained in the current study have provided much valuable information on the precise molecular nature of trace concentrations of Ni(II) present in human saliva together with their concentration-dependent order of formation, and this potentially offers bioavailable and translocating types of this potentially toxic metal ion in this biofluid.

Application of the above novel experimental design protocol also clearly demonstrated that the sequential removal of the strongly interfering between-participant effect and the ‘between-added Ni(II) ion concentrations × between-participants’ first-order interaction variance-contributing effect (the latter accounting for non-additive peculiarities in (heterogeneities of) the differential biomolecular responses of individual participants’ WMSS samples to increasing concentrations of added Ni(II)) sequentially and substantially enhanced the FDR (Bonferroni)-corrected univariate statistical significance of the resonance changes in the metabolite signals. This interaction effect is also at least partially explicable by the visualisation of lag Ni(II) concentrations prior to any changes being observed at higher added levels, which for selected biomolecules were observable for some study participants but not for others, this effect probably arising from the availability of higher concentrations of competing Ni(II) chelators.

However, when statistical comparisons were made between the untreated control samples and those treated with only the lowest added Ni(II) concentration (71 µmol/L), major differences were found between these results and those of the overall full concentration range study conducted. Indeed, biomolecule resonances which responded to such low added concentrations were found to represent a spectrum of the amino acids histidine and taurine > proline, ornithine, phenylalanine, tyrosine, and glutamate along with methylamines and TMAO, ethanolamine, creatine, phosphocreatine, creatinine, choline, and 5-aminovalerate and also visually observable ethanol. However, propionate and lactate were also included in this prime set of salivary Ni(II) complexants. The eloquent switch of stronger, less concentrated amino acids and other Ni(II) donor molecules to less powerful, albeit much higher salivary level carboxylato ligands in WMSS samples appears to occur in our ^1^H NMR-based datasets from added Ni(II) levels of ca. 140 µmol/L. This is because higher levels of such ligands are required for Ni(II) complexation when it is present at concentrations which are greater than that of the primary complexants/chelators available, e.g., amino acids such as histidine (as noted in Table S5), and hence, this second phase of spectral modifications occurring, which predominantly includes carboxylate anion O-donors, accounts for this. Therefore, this serves to explain the Ni(II) concentration lag phases observed for the broadenings of the normalised intensities of acetate, formate, and lactate, for example (Figures S6.1 and S7.1) and also the notable decrease in the selected resonance intensities observed for the first phase of the largely amino acid/amine complexants (group 4 > group 3) at added Ni(II) concentrations of ≤140 or 280 µmol/L.

An exhaustive list of stepwise stability constants for complexes of Ni(II) with a range of biomolecules, along with their literature sources, is provided in Table S5 of Section S12. Hence, the above orders of ^1^H NMR resonance broadenings observed are concordant with the abilities of these complexants/chelators to compete for added Ni(II) ions, firstly in terms of thermodynamic equilibrium constants for the stability (formation) of their complexes (and which are cumulative in situations where 1:1, 1:2, and/or 1:3 complexes may be generated), specifically, amino acids/amines > citrate ≈ succinate > lactate ≈ glycolate > acetate ≈ propionate ≈ *n*-butyrate ≈ formate, and secondly, albeit more importantly, their absolute WMSS concentrations [17,39]. Indeed, the mean human salivary concentrations of lactate, succinate, propionate, *n*-butyrate, formate, acetate, and pyruvate have been previously reported as values ranging from 1 to >100 mmol/L [17], and these parameters, along with those for many further potential salivary Ni(II) complexants, are also provided in Table S5. Notably, salivary organic acid anions generally have much higher salivary concentrations than amino acids and further N-donor function ligands [17,39], and although the latter predominantly have higher or much higher stability constants, the higher concentration factor for salivary carboxylato ligands appears to exert a dominating effect for added Ni(II) complexation when this metal ion is present at levels > 140 µmol/L. However, cumulative stability constants for the 1:1 and 1:2 Ni(II)-amino acid complexes are generally within the 10^5^–10^11^ and 10^10^–10^20^ ranges, respectively.

With the exception of lactate, the carboxylate ligands acetate, *n*-butyrate, formate, and propionate all form similarly thermodynamically stable complexes with Ni(II) (Table S5). However, the mean concentration of acetate in the WMSS samples is approximately five-fold greater than the next nearest carboxylic acid anion level (i.e., that of propionate), and therefore, it will be expected to compete more effectively for any available Ni(II) ions.

### 4.2. Previous Investigations of Ni(II) Speciation in Human Biofluids

Although rather limited, there are a number of reports available on investigations focused on the speciation of Ni(II) ions in biofluids, but to date, none of these have employed high-resolution NMR analysis as an investigational technique. Moreover, few or none of these were focused on human saliva. As early as 1972, Van Soestbergan and Sunderman [40] examined the molecular nature of nickel complexes in blood serum and urine following the injection of radioactive ^63^NiCl_2_ into rabbits at a dosage level of 0.24 mg Ni/kg body mass, and primarily, they found that this isotope was rapidly cleared from the serum during the 1–48 h duration post-dosing (t_1/2_ = 8.2 h), whereas its clearance was slower (t_1/2_ = 95 h) within the 4–7 day period thereafter. During the first 24 h period subsequent to injection, a high proportion (90%) of the mean serum ^63^Ni concentration was albumin-bound, the remaining 10% being low-molecular-mass complex based (i.e., it was ultra-filterable). Further experiments involving Sephadex G-25 chromatography revealed no fewer than five distinct ^63^Ni (presumably Ni(II)) complexes present in these ultrafiltrates. Likewise, of the average of 78% of ^63^Ni excreted in the urine, three of its low-molecular-mass complexes detected had identical chromatographic mobilities to those of rabbit serum ultrafiltrates. Although the precise chemical identities of these complexes were not determined, it appeared that one of these complexes resembled a histidinato-Ni(II) species, information which is of much relevance to the current study. From this pioneering work, the authors concluded that such low-molecular-mass Ni(II) complexants present in biofluids exert an important physiological homeostatic activity for nickel ions by acting as diffusible vehicular receptors for their extracellular mobility and renal excretion.

Similarly, Asato et al. [41] subsequently explored the binding of ^63^Ni (10–100 µmol./L, levels similar to those of the experiments performed in the current study) to ultra-filterable agents present in rabbit blood serum following its equilibration with whole serum in vitro (2.0 h at 37 °C) and at increasing time points after its administration (40–160 µmol/kg body wt.) to these animals *in vivo* (0.25–22.00 h). These investigations revealed that although the mean percentage of ultra-filterable ^63^Ni was 36% of the total present in the in vitro studies conducted, that from the *in vivo* experiments was only 15%. However, this disparity was precluded when experiments were repeated using bilaterally nephrectomised rabbits with ligated common bile ducts. In view of their reactivities with ninhydrin, it appeared that all such ultra-filterable species consisted of those featuring Ni(II)-complexed amino acids, an observation which fits in with our results, but only those obtained at lower, albeit more physiologically relevant added Ni(II) concentrations.

Although valuable, such studies were somewhat restricted since, like ours, they involved high doses (i.e., much greater than normal physiological intakes of naturally abundant Ni). Moreover, it has been speculated that sample contamination occurring throughout their collections and dietary intakes of this metal ion may have adversely contributed to variations in the results acquired [42].

In 1998, Nomoto and Sunderman [43] explored the molecular nature of some Ni-containing complexing agents in human serum, and they discovered associations of this metal ion with α2-macroglobulin and albumin along with ultra-filterable complexes such as those containing amino acids. Correspondingly, the findings of Nielsen et al. demonstrated that nickel(II) forms complexes with the proteins prealbumin, albumin, α2-macroglobulin, α1-antitrypsin, and α1-lipoprotein [44]. Further investigations of Ni(II)-complexing proteins (one using a protein blotting approach with a radioactive ^63^Ni tracker) were reported in [45,46].

Of special interest to the current study, in 1985, Cole et al. [47] conducted a computer simulation study of the molecular nature and distribution of both cadmium(II) and nickel(II) ions amongst low-molecular-mass biomolecular ligands in human blood plasma. These complex distributions were computed for almost 50 different biofluid ligands, and the salient formation constants required for the metal ion complexes considered were experimentally determined under physiological conditions. From these experiments, it was found that nickel(II) was largely in the form of a ternary complex involving cysteinate and histidinate amino acid ligands, whereas Cd(II) predominantly existed as a binary cysteinate complex.

Previously reported NMR-linked investigations of the use of NMR analysis for the speciation of other (non-Ni(II)) metal ions in human body fluids are outlined in Section S13.

### 4.3. Summary of Previous Investigations Focused on Ni(II) Ion Speciation in Plant-Based Biosystems

Since previous investigations of the speciation status of Ni(II) ions in biological systems have been dominated by those focused on plants, most especially Ni(II)-hyperaccumulating species, this section briefly reviews the results acquired from such studies. In 2008, Montarges-Pelletier et al. [48] explored the Ni(II)-complexing abilities of chelators present in three different Ni-hyperaccumulating plants, and for this purpose, X-ray absorption spectroscopy at Ni K-edge was employed to analyse plant leaves, stems, and roots. Their Ni K-edge analysis results were compared with those of aqueous solutions of Ni(II) complexes containing a range of different biomolecular chelators. The major ligands responsible for Ni(II) transference in these plant systems were found to be citrate and malate, with the former serving as the predominant complexant in the stems of Leptoplax and Alyssum and the latter acting as the major Ni(II) chelator within the leaves of all three plants evaluated. However, L-histidine was undetectable in all parts of the plant samples analysed in this manner.

In Ni(II)-hyperaccumulating plants, originally, the amino acid histidine was identified as the major Ni(II) complexant in the genus Alyssum; indeed, its xylem concentration has been shown to escalate with increasing uptake of this metal ion [49,50]. However, for a number of the hyperaccumulating plant species explored earlier, the ubiquitous metal ion chelator citrate was found to be the most important chelator for Ni transport [51,52].

Nevertheless, as with our studies, it may be that the speciation status of plant Ni(II) ions is critically dependent on the amount hyperaccumulated and that relatively high levels of it are predominantly detectable as lower-affinity citrate and malate complexes in view of the availability of higher or much higher plant levels of these chelators and, conceivably, also that of available Ni(II) over those of amino acids such as histidine. Relevant information on risk factors for the dietary Ni(II) intake in humans is provided in Section S14 of the Supplementary Information.

### 4.4. Cellular Uptake of Ni(II) Complexes and Particulates Generated Therefrom

Notably, the precise molecular nature of the Ni(II) complexes arising from the interaction of structurally simple Ni(II)_aq._ species, including corrosion-released adducts with salivary biomolecular ligands or chelators, undoubtedly plays an important role in the adverse toxic effects exerted by this metal ion *in vivo*, notably its inflammatory, allergenic, and carcinogenic properties. Indeed, the charge or electronic neutrality, molecular size, overall structural nature, and dimensions of such complexes will readily influence their ability to cross cell membranes into cells and thence, perhaps, into target organelles such as the nucleus via intracellular targeting mechanisms (processes which present some major barriers [53]), where they may subsequently exert their key toxicological actions, if any. Moreover, if formed in sufficient quantities, polymeric Ni(II) complexes such as some of its carboxylato-Ni(II) adducts may be recognised as ‘foreign’ by phagocytic cells such as macrophages, and hence, may be directly phagocytosed as such. A similar consideration may be made for nickel particulates released from dental alloy materials.

Unfortunately, there are only a limited number of published reports available on cellular uptake mechanisms, and to date, the intracellular accumulation of the anti-cancer square planar platinum(II) drug cisplatin has received much of the attention [54]. Such Pt(II) drugs may enter cells via a series of routes, including passive diffusion, the actions of organic cation transporters, and potentially also by endocytosis. Notwithstanding, the high-affinity copper ion uptake protein and transporter Ctr 1 may also be involved in this process. Hence, the passage of Ni(II) through cellular membranes may feature similar or related transference mechanisms. Moreover, lipophilic metal ion cation complexes or uncharged non-polar ones, can diffuse across the plasma membrane in reciprocation to the membrane potential. Interestingly, in 2007, Ke et al. [55] reported the use of fluorescent tracking to explore the transfer of nickel compounds and complexes into cultured human cells in vitro and found that both water-soluble and -insoluble Ni compounds (as nickel(II) chloride and sulphide, respectively) were taken up by cells, which gave rise to Ni ion accumulation in cytoplasmic and nuclear sites. However, on termination of the nickel compound exposure, Ni ions persisted in cells exposed to Ni_3_S_2_ for lengthier durations than in those exposed to NiCl_2_ in both the nucleus and the cytoplasm. The authors therefore proposed a probable explanation for known differences in carcinogenicity between water-soluble and -insoluble Ni compounds in experimental animals and indicated possible mechanisms for why the latter are more potent carcinogens than the soluble species [55].

### 4.5. Influence of the Speciation Status of Ni(II) Ion on Its Ability to Induce Oxidative Stress In Vivo: Toxicological and Pro-Carcinogenic Ramifications

Ni(II) has been found to propagate lipid peroxidation and enhance DNA strand breakages both *in vitro* and *in vivo* [56], processes which may indeed promote the potential roles of bioactive Ni(II) complexes in the pathogenesis of a number of cancer conditions [57,58,59]. Nevertheless, Ni(II)-promoted oxidative stress is quite restricted in view of a series of physicochemical considerations [59]. Aqueous Ni(II) ions are not powerful generators of reactive oxygen species (ROS) from dioxygen, hydrogen peroxide (H_2_O_2_), and conjugated hydroperoxydienes, the latter produced from the peroxidative degradation of polyunsaturated fatty acids *in vivo*. However, such reactions are controlled by chelation or complexation of this metal ion by biomolecular ligands, for example, L-histidine or L-cysteine [47,60]. Indeed, these complexation reactions exert a major influence on the thermodynamic redox potentials of the Ni(III)/Ni(II) couple, and in some cases, a significant lowering of such values may warrant the participation of Ni(II) ions in pseudo-Fenton-type reactions (Equation (1)).

Ni(II) + H_2_O_2_ → Ni(III) + ^●^OH + OH^−^ (1)

A series of *in vivo* investigations has previously demonstrated peroxidative cell and tissue damage induced by the toxicological actions of Ni(II), and this provided evidence for the adverse production of ROS as an important phase of this process [57]. Indeed, with 5,5′-nitrilobarbiturate (murexide) as a bleaching marker, Torreilles and Guerin [61] investigated the abilities of Ni(II) complexes of histidyl peptides to catalyse metal ion-dependent electron donation to hydrogen peroxide (H_2_O_2_) in order to generate oxidant species with ‘^●^OH radical-like’ reactivities. It was found that peptides with the glycyl-glycyl-L-histidyl peptide chain gave rise to the nickel ion-dependent generation of ROS, which could cause damage to proteins, deplete sample tryptophan contents, and produce significant levels of the bi-tyrosine radical attack product along with the triggering of polyunsaturated fatty acid peroxidation. These researchers also found that the peptide histidine residue was damaged selectively (presumably in a site-specific manner) and that peptide degradation by this mechanism suppressed further ^●^OH or ‘pseudo-^●^OH’ radical generation.

In an intriguing study, Lin et al. [62] explored the ability of Ni(II) complexes to capture carbon-centred radical species and then participate in the production of C-C bonds from Ni(III) intermediates, which were detectable by electron paramagnetic resonance (EPR) spectroscopy.

Of notable further interest, the production of reactive free radical species via the reactions of nickel(II)-thiolate complexes with molecular dioxygen and model conjugated hydroperoxydienes was explored by EPR analysis with 5,5-dimethyl-1-pyrroline-N-oxide (DMPO) as an ^●^OH radical spin trap [63].

### 4.6. Limitations of the Study

One major limitation of the current study is the enhanced range of final concentrations of Ni(II) added to the WMSS samples during the ^1^H NMR titrations conducted. Indeed, in our experiments, this was ca. 100- to 1000-fold greater than the mean human saliva concentration determined in previous studies (75 nmol/L, Ref. [21]). However, this high range of Ni(II) ion concentrations was primarily selected in order to provide a full, extreme scope of the biomolecular complexants present in this biofluid, and in this manner, the sequential dependence of such effects on added concentrations of this metal ion could be monitored. Moreover, at these added Ni(II) concentrations, the changes occurring to the WMSS sample resonances were readily visible with ^1^H NMR. However, it is conjectured that valuable ^1^H NMR results might also have been achieved using lower levels of added paramagnetic Ni(II) ions, perhaps those ranging from 10 or more -fold lower than those employed herein.

Although this investigation would have benefitted from the performance of computer-based simulations of the molecular nature of Ni(II) in human saliva along with their added metal ion concentration dependencies, such lengthy studies, which are considered to be of critical importance, will be performed in our laboratory in the near future and will be reported and published elsewhere. This future computer simulation study will be conducted in conjunction with laboratory ^1^H NMR titrations conducted at the above lower added Ni(II) levels, i.e., perhaps from 5 to 100 µmol/L. Nevertheless, such biofluid computational simulation information is already available, but for human blood plasma only [47], where it was predicted that low-molecular-mass Ni(II) was predominantly present in the form of a ternary Ni(II)-histidinate/cysteinate complex.

A further limitation is the overlap of resonances, which do occur despite the high NMR operating frequency applied here. Attempts to limit this complication were of course applied, but in situations where it was not possible to distinguish between superimposing resonances, combinations of these are recorded in all assignment tables provided. A further complication is that in view of the fixed bucket width protocol applied, a small number of ^1^H NMR signals were at least partially ‘split’ into two adjacent buckets, for example, the 4.08–4.12 and 4.12–4.16 ppm ones for the lactate-CH resonance. Although such signal overlap was readily resolvable using 2D homonuclear spectroscopy (e.g., in ^1^H-^1^H COSY spectra, Figure 3), the problems associated with narrow bucket superimpositions of integratable resonances present in 1D spectra remained. However, in the low- and high-field regions of the spectra acquired, the number of such overlapping signal issues remained quite low, whereas there were, of course, larger numbers of these in the more crowded mid-field region. The acquisition of 2D COSY spectra certainly facilitated or confirmed the assignment of a series of the resonances found in 1D spectra and identified the molecular nature of a significant number of further signals.

Additional considerations include the participant sample size recruited to this study (n = 12), which may appear to be somewhat limited. However, it should be noted that 7 × ca. 0.60 mL aliquots of each sample, which were originally collected as quite high volumes, were individually treated with increasing levels of Ni(II)_aq._ (from 0.00 to 0.67 mmol/L), and n = 3 replicate spectra were acquired for each control or Ni(II)-treated sample.

Although largely exhaustive in the current study, the application of further MV analysis and computational intelligence analysis strategies for segregating differential variance contributions and isolating those of interest, e.g., self-organising map analysis strategies [64] for Ni(II)-interactant clustering class studies, may indeed offer approaches for further optimising the analysis of metal ion speciation datasets, NMR-based or otherwise.

Finally, unfortunately, to date NMR techniques have often been plagued with ‘bad press’ regarding their apparent limited sensitivity, although such criticism often arises from those who are unaware of or have simply not kept up-to-date with the rapidly expanding technological developments in this area, sensitivity-enhancing or otherwise. Indeed, the direct high-field ^1^H NMR analysis of human biofluids such as WMSSs can now be viewed as a high-sensitivity technique, and at operating frequencies of 600 MHz or above, it is now possible to detect, and also determine, metabolite concentrations of less than several µmol/L, this being achievable without the pre-application of any extraction or pre-concentration strategies. Furthermore, its virtual non-destructive nature permits the use of more sensitive, albeit destructive, techniques following this analysis.

## 5. Conclusions

This study represents the very first investigation of the application of state-of-the-art ^1^H NMR-based metabolomics technologies to promote the rapid identification of and to evaluate the relative importance of a series of biomolecular complexants/chelators for bioactive and/or toxic metal ions, in this case for Ni(II) added to human saliva in vitro. Indeed, the application of such metabolomics strategies saves much valuable time by avoiding the lengthy and arduous computer-based spectro-visual inspection of individual spectra sequentially by trained laboratory staff. Employment of covariate-balancing factorial ANOVA design analyses found that the salivary ^1^H NMR resonances most affected by the consideration of effects observed at **all** added Ni(II) levels (i.e., broadenings and intensity reductions) by added Ni(II) were those assignable to formate > acetate > lactate > propionate > pyruvate/succinate > *n*-butyrate > leucine/isoleucine > alanine > glycine, in that order. With a number of exceptions, including ethanol and free N-acetylneuraminate, these data were found to be in good agreement with results obtained from the direct spectral scrutinisation methods applied (Figure 1 and Figure 2 and Table 1). However, crucially, at low added Ni(II) concentrations only, amino acids such as histidine and taurine, methylamines, and further N-donor atom biomolecules were found to represent the most important Ni(II) complexants. Hence, at low added Ni(II) levels (≤ 71 µmol/L), the complexation of adventitious, dietary, or dental metal alloy-released Ni(II) appears to primarily involve low-concentration amino acids such as L-histidine, etc., and they were distinguishable as class 4 of the Ni(II)-responsive metabolites found here. However, at higher added concentrations, a secondary order of Ni(II) complexation was dominated by oxygen-donor carboxylato ligands (defined as class 1 here). These phenomena largely reflect the overall sensitivity of the WMSS ligand/chelator ^1^H NMR signals to added Ni(II) and verify that the metabolites tracked in this manner compete for this metal ion, a phenomenon contingent on (1) their available WMSS concentrations, (2) their highly variable Ni(II)-complex stability constants (Table S5), and, critically, (3) the amount of Ni(II) available for complexation together with a series of physicochemical considerations such as localised pH and ionic strength values, etc. Hence, data acquired in the current study are clearly of much relevance to the molecular reactivity, and hence, the mechanisms for the toxicity of this metal ion, which may be dietarily consumed or corrosively liberated from NiC-MADPs *in vivo*.

## Figures and Tables

**Table 1:** Salivary metabolite ^1^H NMR resonances influenced by the addition of increasing Ni(II) concentrations, along with their assignments. The added Ni(II) concentrations at which changes in these resonances were first observed are indicated. This order was based on the direct visual inspection of the spectra acquired. Abbreviations: GABA, γ-aminobutyrate; n/a, not applicable. * Only visible in ca. 60% of spectra acquired. ** Although participants were requested **not** to consume any alcoholic beverages for a minimum duration of 24 h prior to sample donation, low levels of ethanol were still detectable in ca. 30% of the samples collected.

Added [Ni(II)] (µmol/L)	Resonance(s) (ppm)	Biomolecule ^1^H NMR Assignment
71	1.33 (*d*) and 4.13 (*q*)	Lactate-CH_3_ and -CH, respectively
	2.41 (*s*)	Succinate-(CH_2_)_2_
	7.07 (*s*), 7.84 (*s*), and 3.15/3.22 (*m*)	Histidine imidazole ring-C4H and -C2H, and β-CH_2_ protons, respectively *
	1.17 (*t*) and 3.66 (*q*)	Ethanol-CH_3_ and -CH_2_, respectively **
140	8.46 (*s*)	Formate-H
	2.39 (*s*)	Pyruvate-CH_3_
280	3.56 (*s*)	Glycine-CH_2_
	1.05 (*t*) and 2.17 (*q*)	Propionate-CH_3_ and -CH_2_, respectively
	1.92 (*s*)	Acetate-CH_3_
	2.06 (*s*) and 1.82 (*dd*)	N-Acetylneuraminate-NHCOCH_3_ and -C3H (β), respectively
	2.13 (*s*)	Methionine-SCH_3_
	3.93 (*s*)	Glycolate-CH_2_
	3.97 (*s*)	Creatine (Cr)-CH_2_
	3.99 (*s*)	Phosphocreatine (PCr)-CH_2_
410	1.48 (*d*)	Alanine-CH_3_
	1.64 (*m*), 2.23 (*t)*, and 3.00 (*t*)	5-Aminovalerate-3/4-(CH_2_)_2_, -2-CH_2_, and -5-CH_2_, respectively
	1.16 (*d*), 2.61 (*m*), and 3.02 (*dd*)/3.10 (*dd*)	3-Aminoisobutyrate (3-AIB)-2-CH_3_, -2-CH, and -3-CH_2_, respectively
	1.24 (*d*), 2.31 (*dd*)/2.41 (*dd*), and 4.16 (*dt*)	3-D-Hydroxybutyrate-4-CH_3_, -2-CH_2_, and -3-CH, respectively
	8.07 (*s*) and 8.09 (*s*)	Adenine-C2H and -C8H, respectively
540 and 670	3.20 (*s*)	Choline-N(CH_3_)_3_^+^
	3.01 (*t*)	GABA-γ-CH_2_/Lys-ε-CH_2_/5-Aminovalerate-δ-CH_2_/Creatinine-N(CH_3_)_3_
	2.51 (*s*)	Methylamine-N(CH_3_)
	2.76 (*s*)	Dimethylamine-N(CH_3_)_2_
	2.91 (*s*)	Trimethylamine-N(CH_3_)_3_
	6.88 (*d*) and 7.23 (*d*)	Tyrosine-C2H/C6H and -C3H/C5H, respectively
	7.32 (*m*), 7.37 (*m*), and 7.43 (*m*)	Phenylalanine-C2H/C6H, -C4H, and -C3H/C5H, respectively.

## Data Availability

Data presented in this study are available through contact with the correspondence author of this article (email: mgrootveld@dmu.ac.uk).

## Supplementary Materials

The following supporting information can be downloaded at: https://www.mdpi.com/article/10.3390/metabo15010004/s1, Section S1: Possible effect of Ni(II) solution-mediated pH modifications on the ^1^H NMR chemical shift values of WMSS biomolecules; Section S2: Investigation of the ability of salivary proteins to broaden the TSP internal standard/chemical shift reference -Si(CH_3_)_3_ resonance (δ = 0.00 ppm); Section S3: Influence of added Ni(II) ion on the line-width at half-height (Δ*v*_1/2_) value of the TSP internal standard -Si(CH_3_)_3_ ^1^H NMR resonance; Section S4: ^1^H NMR analysis of the pooled WMSS admixture quality control sample; Section S5: Expanded 0.90-1.90, 1.30-2.50 and 3.85-4.40 ppm regions of ^1^H NMR spectral titrations of WMSS samples with Ni(II); Section S6: Details regarding Ni(II) ion-induced modifications to the line-widths at half-height, intensities and chemical shift values of key WMSS carboxylate complexants; Section S7: Preliminary investigations of the dependence of selected resonance intensities on added Ni(II) ion concentrations: Influence of the row-wise PQN normalisation protocol; Section S8: Segregation of fixed ^1^H NMR metabolite buckets according to their concentration-dependent responses to added Ni(II) ion; Section S9: Methods and results arising from the application of covariate- and interaction effect-balancing two-way ANOVA models (direct univariate ANOVA-based analysis); Section S10: Application of a MV ASCA model to analysis of the added Ni(II) ion- and participant-dependent metabolomics dataset; Section S11: Ni(II) ion speciation experiments conducted with the pooled WMSS QC sample; Section S12: Stepwise stability constants for Ni(II)-biomolecule complexes and their literature sources; Section S13: Previously reported NMR-based studies of metal ion speciation in human biofluids; Section S14: Risk factors for dietary Ni(II) intakes in humans. References [42,65,66,67,68,69,70,71,72,73,74,75,76,77,78,79,80,81,82,83,84,85,86,87,88,89,90,91,92] are cited in the Supplementary Materials.
