# Supplementary material for: Speciation of Potentially Carcinogenic Trace Nickel(II) Ion Levels in Human Saliva: A Sequential Metabolomics-Facilitated High-Field 1H NMR Investigation
Source: Metabolites. 2024 Dec 30;15(1):4. doi: 10.3390/metabo15010004 (PMC11768044; doi:10.3390/metabo15010004)
Supplement: Supplementary file 1 [file metabolites-15-00004-s001.zip › metabolites-3284428-supplementary.pdf]

Speciation of Potentially Carcinogenic Trace Nickel(II) Ion Levels in Human Saliva: A Sequential Metabolomics-Facilitated High-Field ^{1}H NMR Investigation

Kayleigh Hunwin, Georgina Page, Mark Edgar, Mohammed Bhogadia and Martin Grootveld *
Leicester School of Pharmacy, De Montfort University, Leicester LE1 9BH, UK.

***Correspondence: Email: mgrootveld@dmu.ac.uk**

Supplementary Information

Section S1: Possible effect of Ni(II) solution-mediated pH modifications on the ^{1}H NMR chemical shift values of WMSS biomolecules

In order to determine the possible influence on biomolecule resonance δ values of any marginal pH changes arising from the titration of marginally acidic $Ni(II)_{(aq.)}$ stock solutions (10.00 mmol/L, final pH 6.53) into naturally-buffered WMSS samples, larger volumes of the latter were collected from n = 3 separate participants, and to 5-fold scaled-up 3.50 mL volumes of these samples was added an equivalently scaled-up aliquot (250 μL) of the above $Ni(II)_{(aq.)}$ solution, this being equivalent to the maximal volume of 50 μL of the metal ion solution added to a 700 μL volume of the biofluid supernatant in our speciation experiments (equivalent to a final added Ni(II) level of 0.67 mmol/L). WMSS pH values were 6.68, 6.71 and 6.62 before stock solution addition, and 6.47, 6.44 and 6.37, respectively, afterwards. Therefore, the mean pH value reduction observed was only 0.25, and with the exception of metabolites with proton pKa values within the 6.50-6.80 ppm range, it was concluded that spectral titration of WMSS samples with a 10.0 mmol/L Ni(II) stock solution as described above was very unlikely to give rise to any significant pH-dependent changes in the chemical shift values of salivary biomolecules monitored in this study. Similarly, a corresponding experiment conducted with HPLC-grade water in place of WMSS samples showed a pH decrease from 7.09 to 6.88, i.e., a reduction of 0.21 only.

Section S2: Investigation of the ability of salivary proteins to broaden the TSP internal standard/chemical shift reference -Si(C$\underline{H}_3$)$_3$ resonance (δ = 0.00 ppm)

An additional further potential bioanalytical issue relates to the ability of salivary proteins and perhaps further macromolecules therein to broaden the TSP internal standard resonance through protein-binding equilibria involving the latter's negative charge and/or other physicochemical attractive phenomena. Therefore, an evaluation of the influence of WMSS proteins on the line-width at half-height ($\Delta v_{1/2}$) values of the TSP resonance when added to n = 7 different WMSS samples at the same level employed for their ^{1}H NMR analysis was undertaken, with comparisons being made against those observed in this NMR standard diluted into an aqueous solution buffered at pH 7.00. The mean±95% confidence intervals (CIs) for this $\Delta v_{1/2}$ parameter for the -Si(C$\underline{H}_3$)$_3$ signal of TSP added to human WMSS samples was found to be 1.79±0.10 Hz, whereas that observed in aqueous solution without any additives besides a pH 7.00 phosphate buffering system was only very slightly lower, i.e. 1.69 Hz (spectra were acquired as outlined in Section 2.2). This difference was not at all statistically significant (two sample t-test). Therefore, from these experiments it was concluded that the concentration of proteins and other biomacromolecules present in the WMSS samples evaluated were not sufficient to affect the intensity, nor line-widths of this TSP resonance, unlike that commonly encountered when it is added to human blood plasma samples which have a much higher protein level, *ca.* 10-fold greater than that of human saliva, as noted in Section 2.1 [24,25].

Section S3: Influence of added Ni(II) ion on the line-width at half-height ($\Delta v_{1/2}$) value of the TSP internal standard -Si(C$\underline{H}_3$)$_3$ ^{1}H NMR resonance

In view of the affinity of Ni(II) ions for carboxylato O-donor ligands, we also preliminarily monitored any enhancements of TSP's $\Delta v_{1/2}$ values with increasing added Ni(II) concentrations in WMSS samples. Such line-width modifications arising from this added metal ion were analysed using a two-way randomized blocks analysis-of-variance (ANOVA) model considered without interactions (as described

in Section 2.4). These data clearly showed that the $\Delta v_{1/2}$ value of the TSP resonance pre-added to WMSS samples (final concentration 229 µmol/L) increased with increasing added Ni(II) concentration (Figure S1), the only non-significant *post-hoc* comparison of means being that observed between the zero control and the 71 µmol/L concentration of added Ni(II). However, overall, statistical significance levels for the 'between-added Ni(II) concentrations' (fixed) and the 'between-participants' (random) effects were p = 2.17 x 10^{-11} and 0.035 respectively.

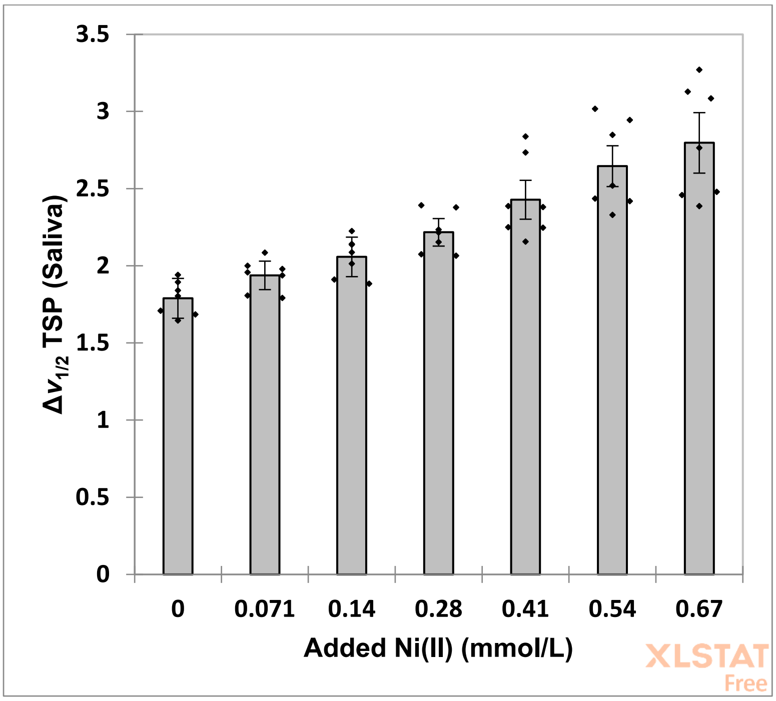

Figure S3.1 Plot of mean±95% confidence intervals (CIs) line-width at half-height ($\Delta v_{1/2}$) values for the TSP resonance *versus* added Ni(II) level for a total of n = 7 WMSS samples and a final TSP concentration of 229 µmol/L. The only Ni(II)-treated samples which did not give rise to a significant increase in $\Delta v_{1/2}$ over that of the untreated zero control value was that with a 0.071 mmol/L added concentration of this metal ion.

Section S4: ^{1}H NMR analysis of the pooled WMSS admixture quality control sample

(a)

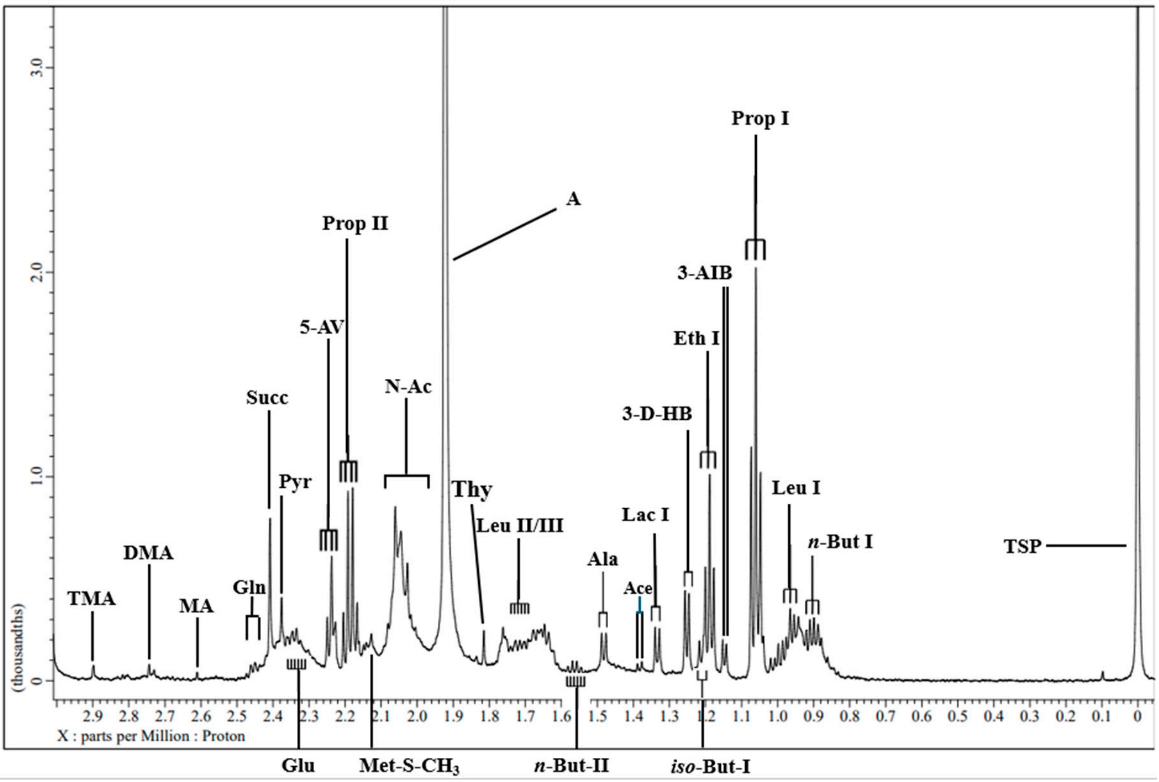

(b)

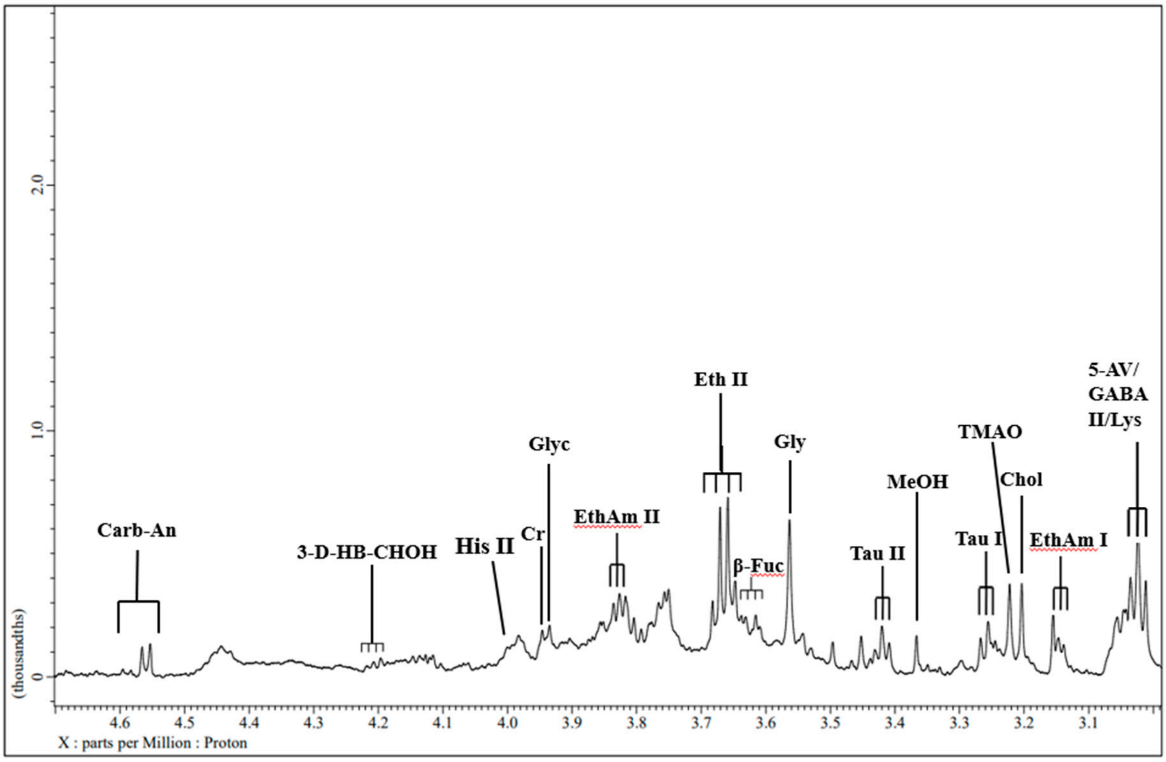

(c)

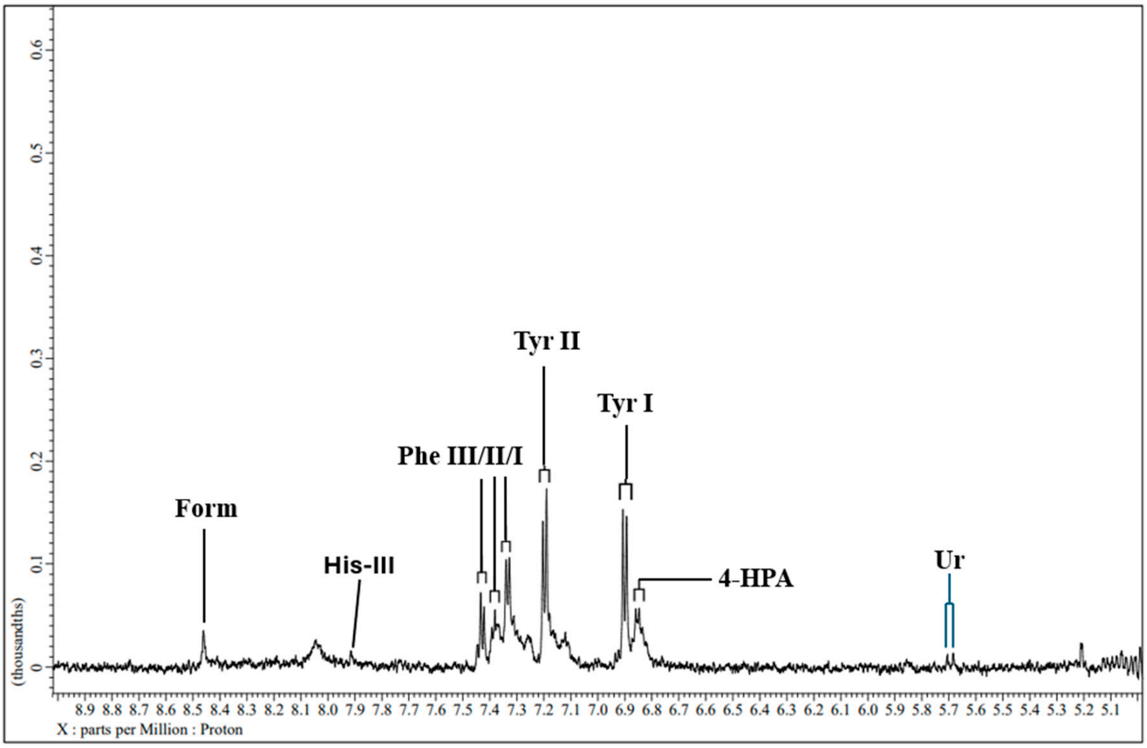

Figure S4.1 Expanded (a) 0.00–3.00, (b) 3.00-4.70 and (c) 5.00-9.00 ppm regions of the 600.17 MHz ^{1}H NMR spectrum of a pooled WMSS QC sample prior to equilibration with increasing added Ni(II) concentrations (0-670 µmol/L). A typical spectrum is shown. Spectra were acquired as described in Section 2.2. Abbreviations: as Figures 1-6, with GABA-II, γ-aminobutyrate-δ-$C\underline{H}_2$; Lys, lysine-ε-$C\underline{H}_2$; EthAm-I and -II, ethanolamine-$C\underline{H}_2NH_2$ and -$C\underline{H}_2OH$ respectively; β-Fuc, β-fucose-C3$\underline{H}$; His-II and -III, histidine-α-C$\underline{H}$ and imidazole ring-C2$\underline{H}$; Ur, uracil-C2$\underline{H}$; Carb-An, 2 x carbohydrate anomeric ring proton resonances (doublets), that at δ = 4.59 ppm possibly arising from β-glucose.

Section S5: Expanded 0.90-1.90, 1.30-2.50 and 3.85-4.40 ppm regions of ^{1}H NMR spectral titrations of WMSS samples with Ni(II)

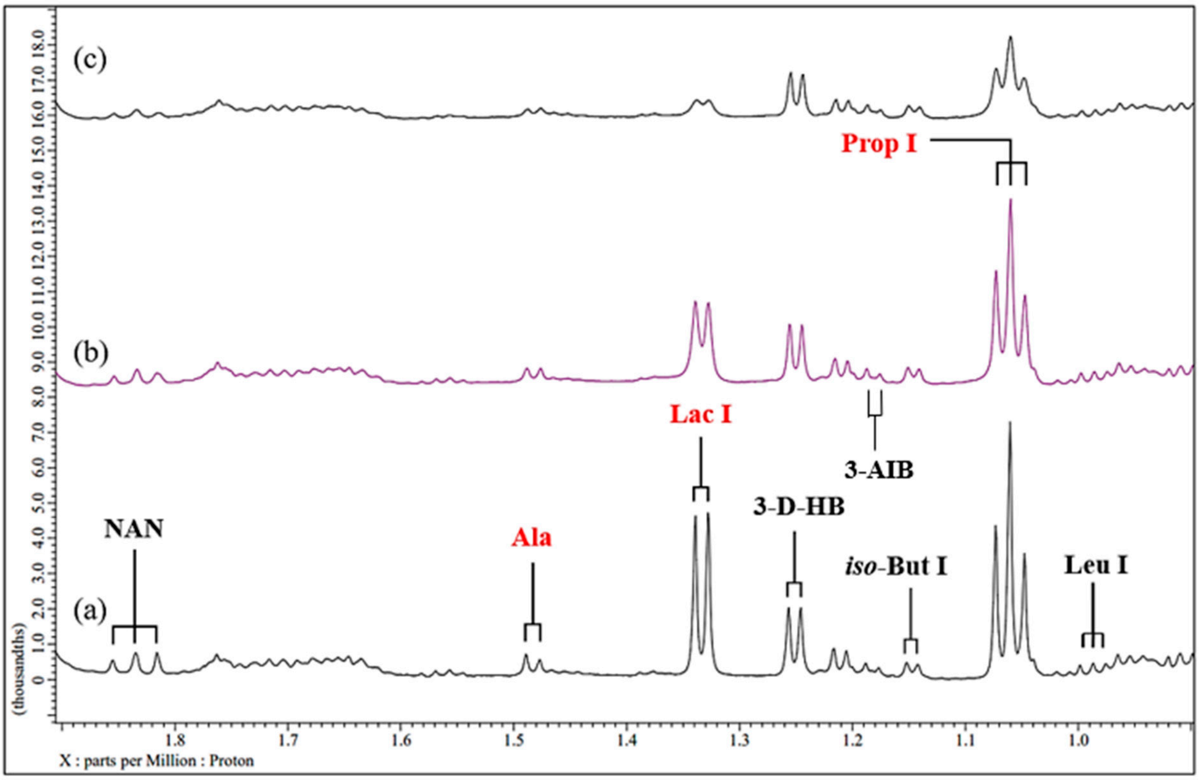

Figure S5.1 Partial (expanded 0.90–1.90 ppm region of) 600.17 MHz ^{1}H NMR spectra of (a) a control human salivary supernatant specimen, and the same sample following the addition of (b) 280 µmol/L, and (c) 670 µmol/L Ni(II). Resonances for the key Ni(II) ion WMSS complexants lactate, propionate and alanine are labelled in red. Typical spectra are shown. Abbreviations: as Figure 1.

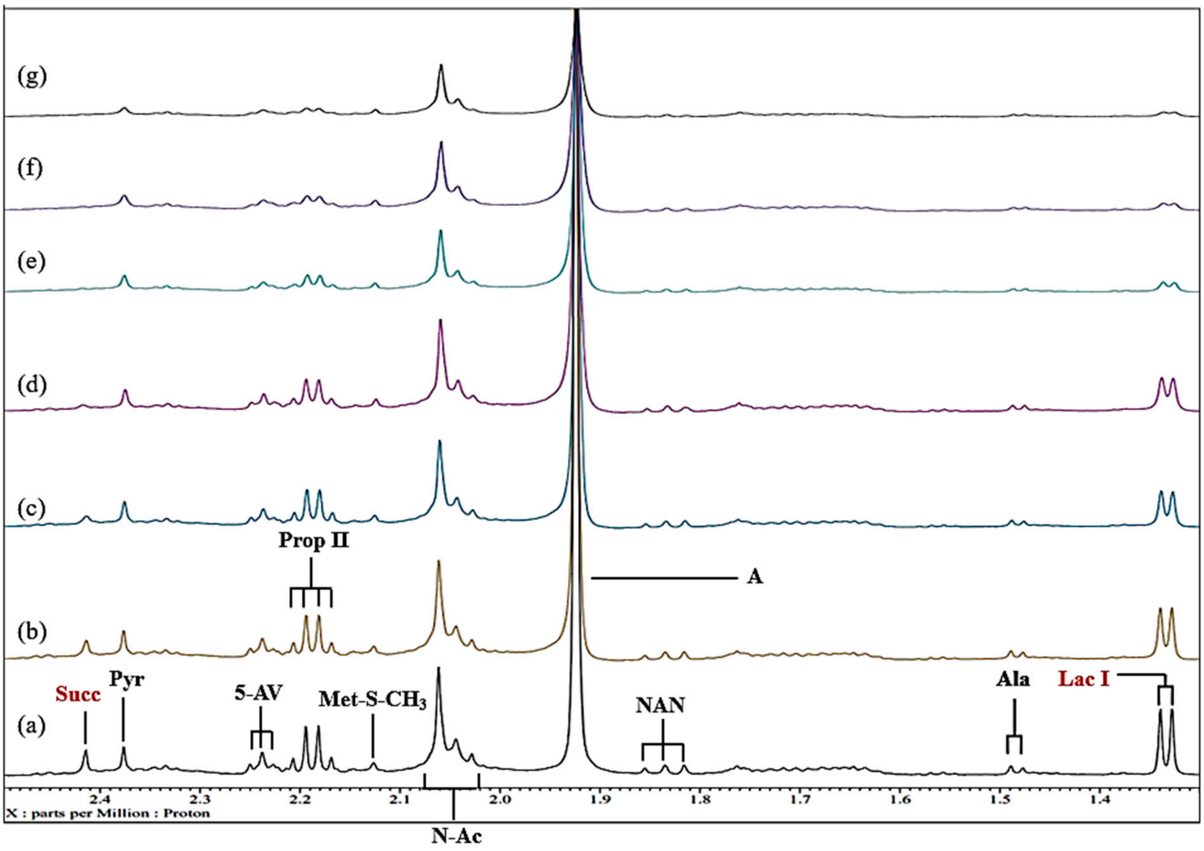

Figure S5.2. Expanded 1.30–2.50 ppm regions of the 600.17 MHz ^{1}H NMR spectra of (a) a control human salivary supernatant specimen, and the same sample following equilibration with Ni(II) at added final concentrations of (b) 71 μmol/L, (c) 140 μmol/L, (d) 280 μmol/L, (e) 410 μmol/L, (f) 540 μmol/L and (g) 670 μmol/L. Typical spectra are shown. Abbreviations: as Figure 1.

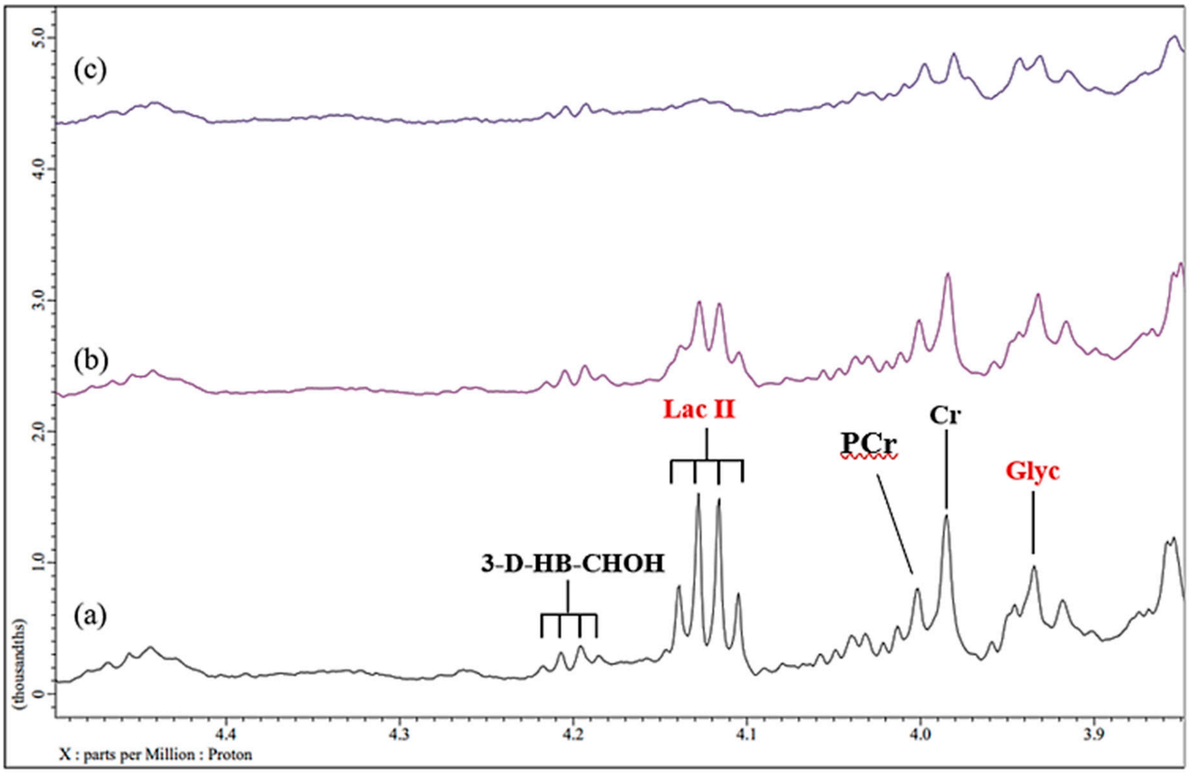

Figure S5.3 Partial (expanded 3.85–4.40 ppm lactate-CH proton region of) 600.17 MHz ^{1}H NMR spectra of (a) a control WMSS specimen, and the same sample following the addition of (b) 280 µmol/L and (c) 540 µmol/L Ni(II). ^{1}H NMR signals for the key WMSS complexants glycolate and lactate are labelled in red. Typical spectra are shown. Abbreviations: As Figure 1, with Glyc representing glycolate-CH_2; Cr, creatine-CH_2; PCr, phosphocreatine-CH_2; 3-D-HB-CHOH, 3-D-hydroxybutyrate-CHOH proton resonances.

Section S6: Details regarding Ni(II) ion-induced modifications to the line-widths at half-height, intensities and chemical shift values of key WMSS carboxylate complexants

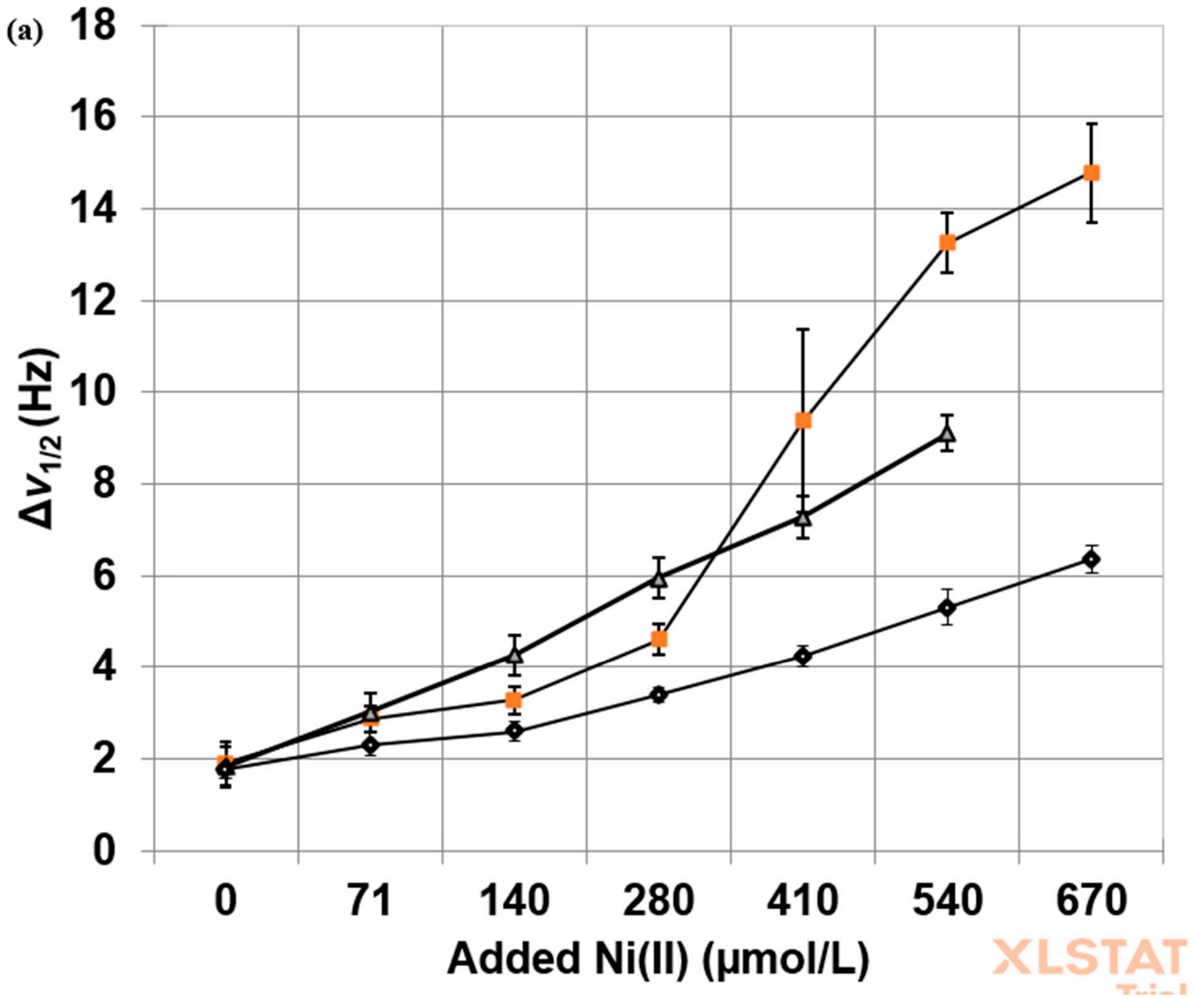

(b)

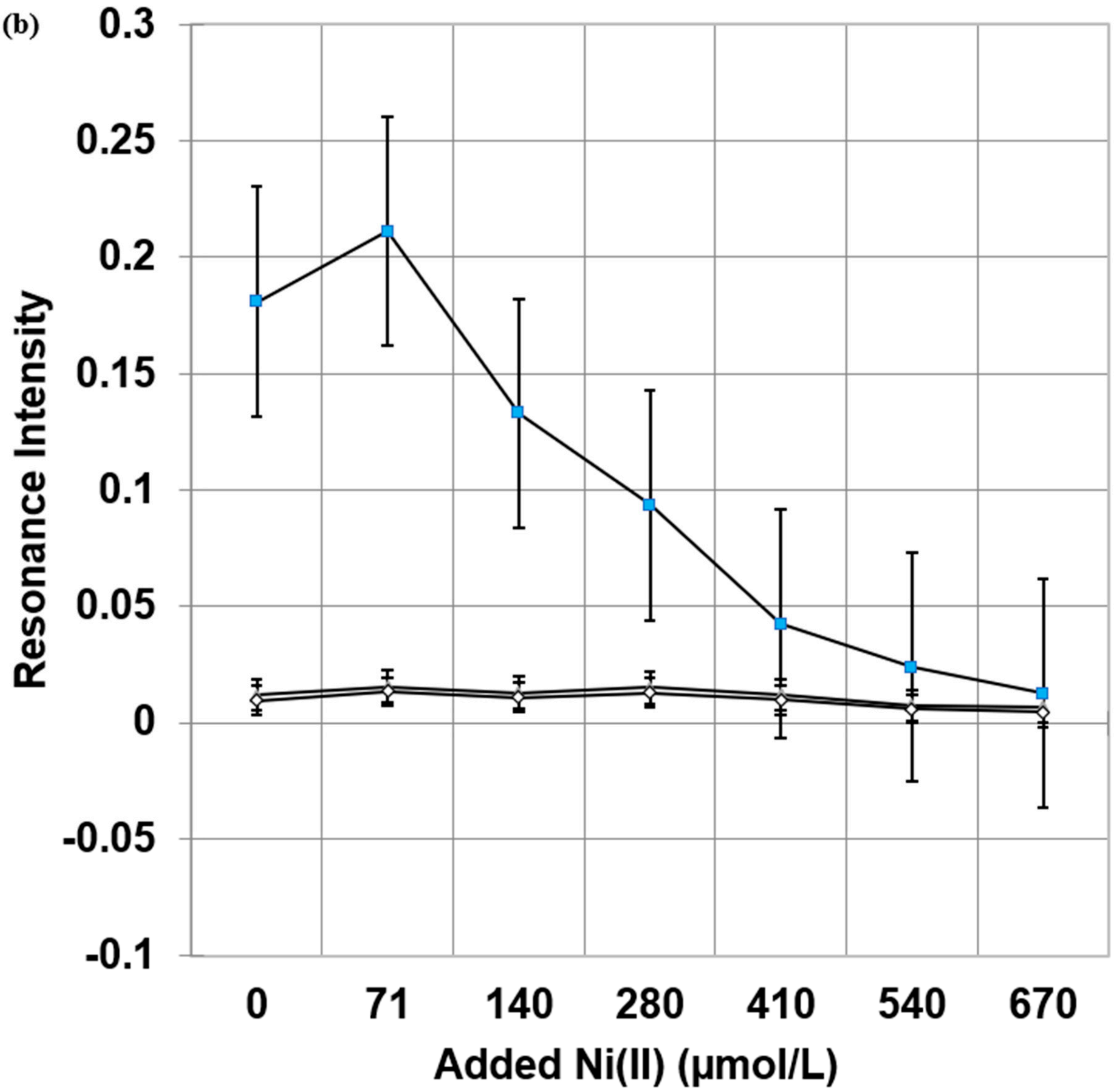
0.3
0.25
0.2
0.15
0.1
0.05
0
-0.05
-0.1
Resonance Intensity
0
71
140
280
410
540
670
Added Ni(II) (µmol/L)

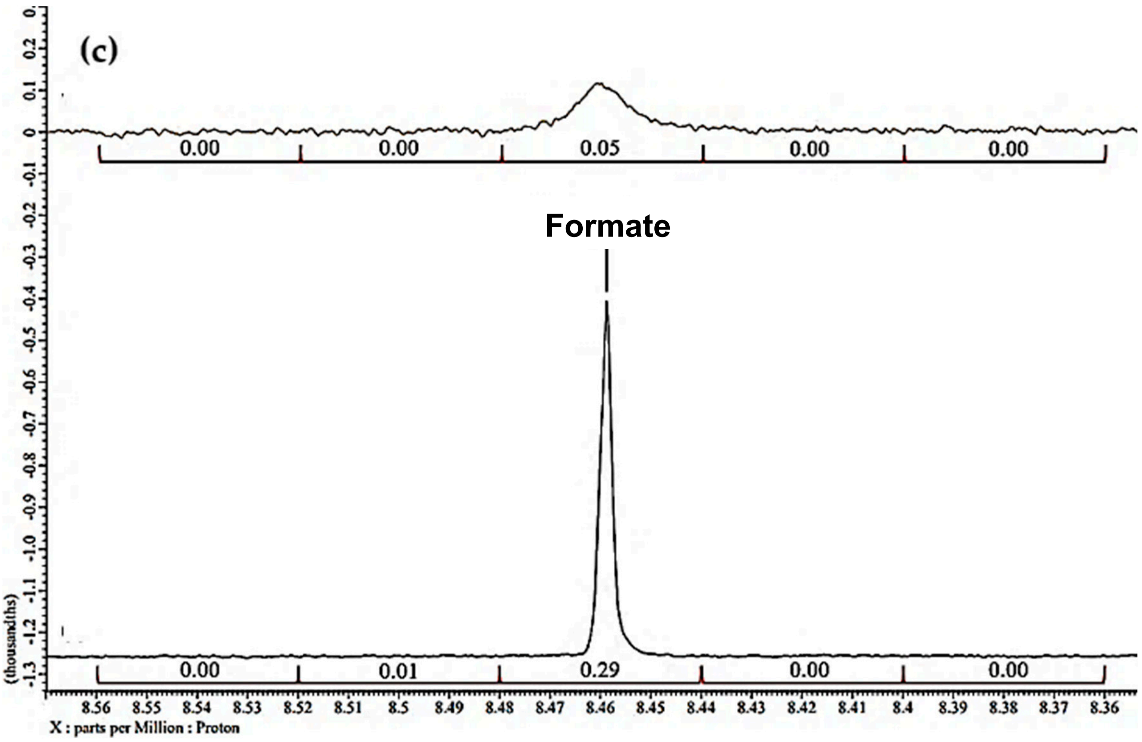

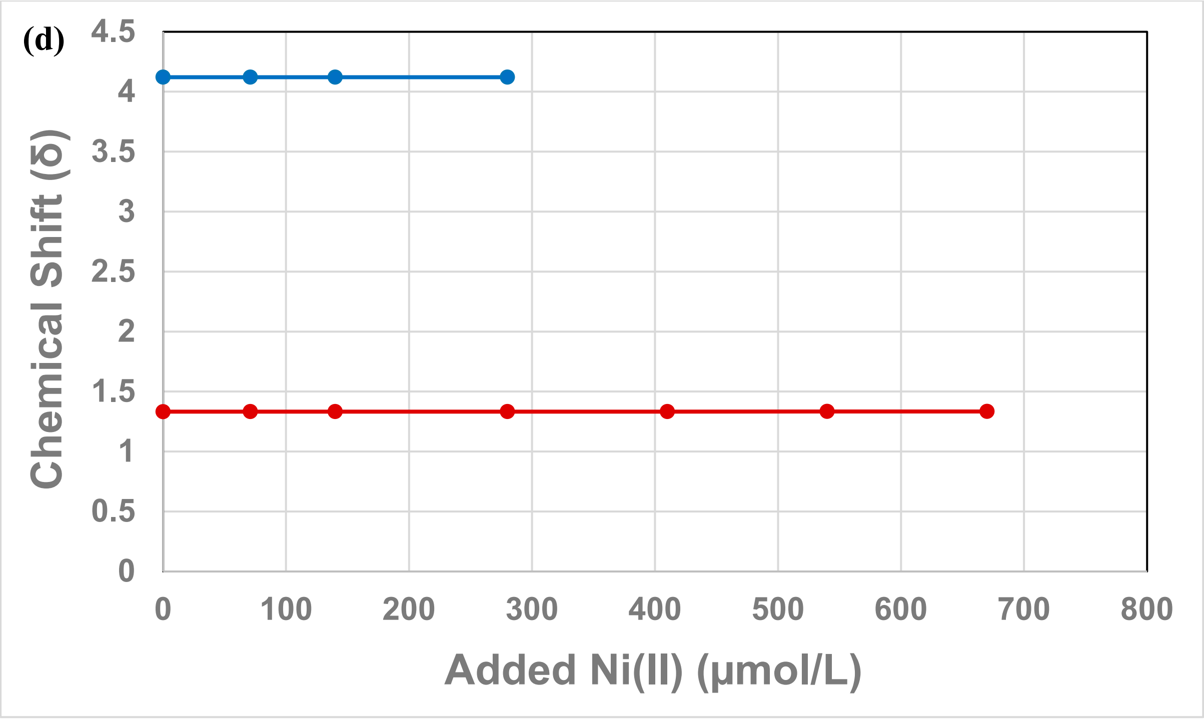

Figure S6.1 Influence of increasing added Ni(II) levels on the line-width at half-height ($\Delta v_{1/2}$) and bucket intensity values of WMSS lactate-CH_3, acetate-CH_3 and formate-H resonances (δ = 1.33, 1.92 and 8.46 ppm respectively). (a) Plots of mean±95% CI increases in the $\Delta v_{1/2}$ values of the lactate-C<u>H</u>$_3$ (orange squares), acetate-C<u>H</u>$_3$ (white diamonds) and formate-<u>H</u> (grey triangles) resonances with increasing added Ni(II) concentrations (n = 7 participants). Since the δ = 8.46 ppm formate resonance had completely disappeared from spectra at an added Ni(II) level of 670 μmol/L, it was not possible to

provide its $\Delta v_{1/2}$ parameter at this concentration. (b) Plot of mean±95% CIs for electronically-integrated TSP-normalised bucket intensities *vs.* added Ni(II) level for the δ = 8.44-8.48 ppm maximal formate resonance 0.04 ppm width bucket (blue squares, resonance centroid at δ = 8.46 ppm), along with that of its two adjacent buckets (δ = 8.40-8.44 (white diamonds) and 8.48-8.52 ppm (white circles)). (c) 8.35-8.57 ppm region of the 600 MHz ^{1}H NMR spectrum of a WMSS sample prior (lower profile) and subsequent to (upper profile) the addition of a final Ni(II) concentration of 270 μmol/L, with localised 0.04 ppm buckets shown around the central formate signal one (δ = 8.44-8.48 ppm). The electronic intensities of the bucketed formate signals were 0.29 and 0.05 for the formate resonance bucket before and after Ni(II) ion treatment, respectively. (d), Influence of added Ni(II) concentration on the chemical shift (δ) values of the WMSS lactate-C$\underline{H}_3$ (red) and -C$\underline{H}$ (blue) proton resonances at an operating frequency of 600 MHz (that for the -C$\underline{H}$ signal was not obtainable at Ni(II) levels >280 μmol/L because of resonance broadening).

A randomized blocks two-way ANOVA model applied to these datasets found that only the formate-specific 8.44-8.48 ppm fixed bucket width had a significant 'between-added Ni(II) concentrations' effect (p = 1.15 x 10^{-6}), which was very highly significant indeed; corresponding differences between the δ = 8.36-840, 8.40-8.44, 8.48-8.52 and 8.52-8.56 ppm buckets were, as expected, not found to be statistically significant. Notwithstanding, with the exception of the 8.48-8.52 ppm bucket, all the others did have highly significant 'between-participants' random effect variances.

Hence, for the case of the carboxylato oxygen atom donor formate, such resonance broadenings were bucket-specific, with little or no influence of added Ni(II) exerted on either of the directly adjacent lower- and higher-field buckets. Moreover, Figure S6(b), which shows plots of the mean±95% CI resonance intensities of the 8.44-8.48 ppm bucket, together with those of its two adjacent δ = 8.40-8.44 and 8.48-8.52 ppm buckets, confirmed this, with little or no effect of added Ni(II) concentration on the latter two (although added Ni(II) was also found to significantly broaden TSP's δ = 0.00 ppm resonance (Figure S1), presumably via its ability to complex Ni(II) through its propionate carboxylate function O-donors, this broadening effect was actually much smaller than those observed for the formate-$\underline{H}$, acetate-C$\underline{H}_3$ and lactate-C$\underline{H}_3$ signals).

Section S7: Preliminary investigations of the dependence of selected resonance intensities on added Ni(II) ion concentrations: Influence of the row-wise PQN normalisation protocol

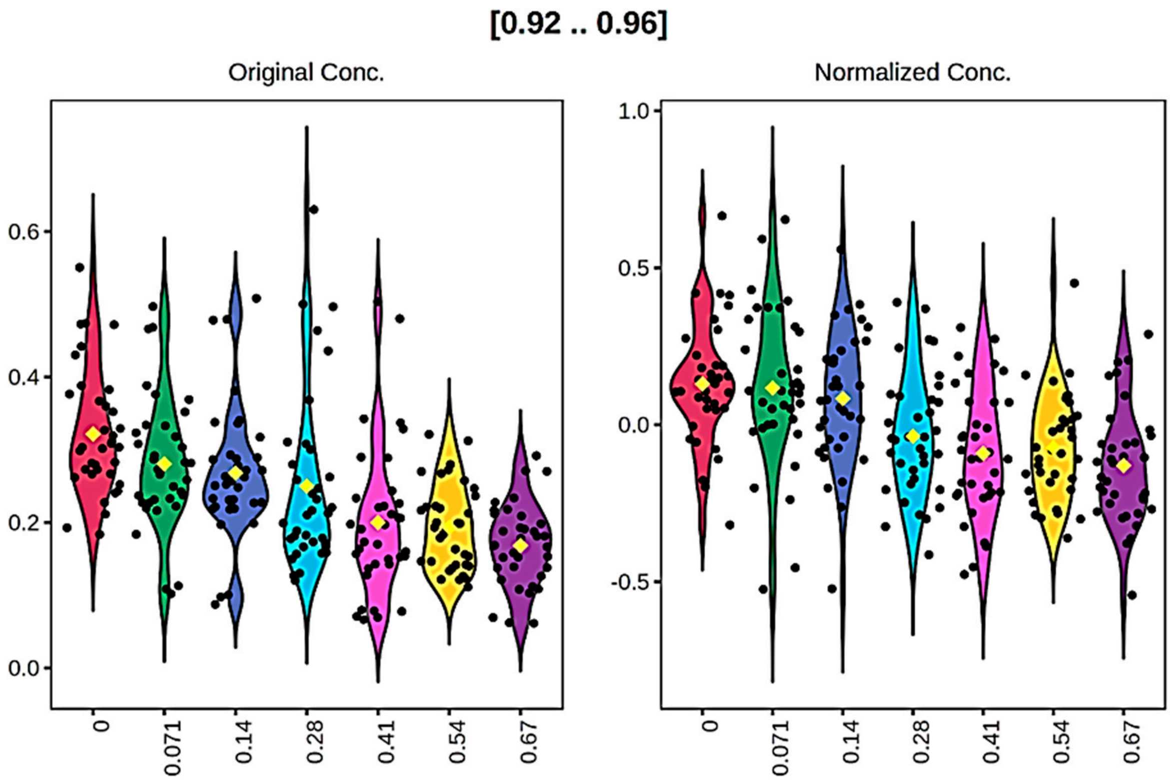

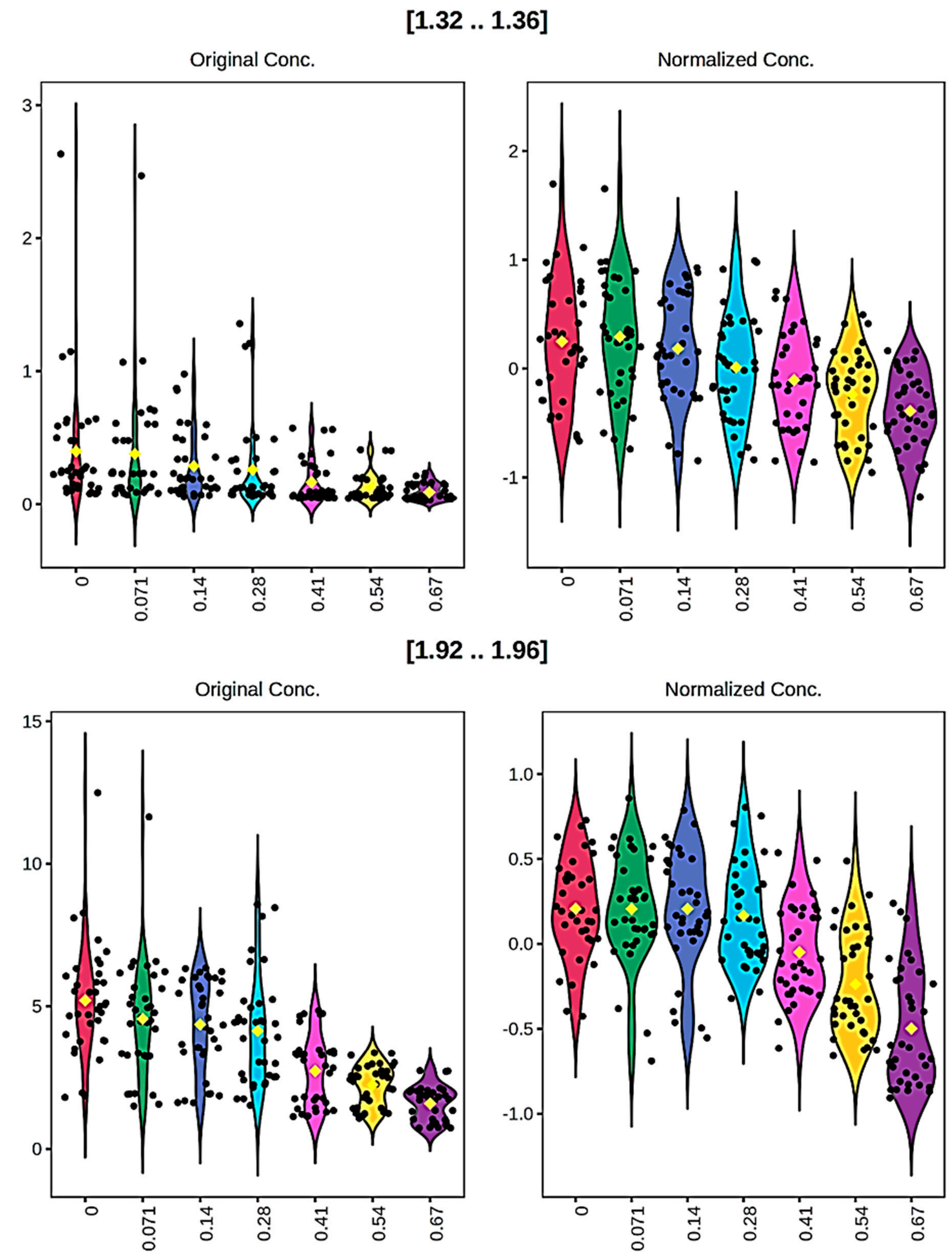
[1.32 .. 1.36]
Original Conc.
Normalized Conc.
0
0.071
0.14
0.28
0.41
0.54
0.67
[1.92 .. 1.96]
Original Conc.
Normalized Conc.
0
0.071
0.14
0.28
0.41
0.54
0.67

[2.40 .. 2.44]

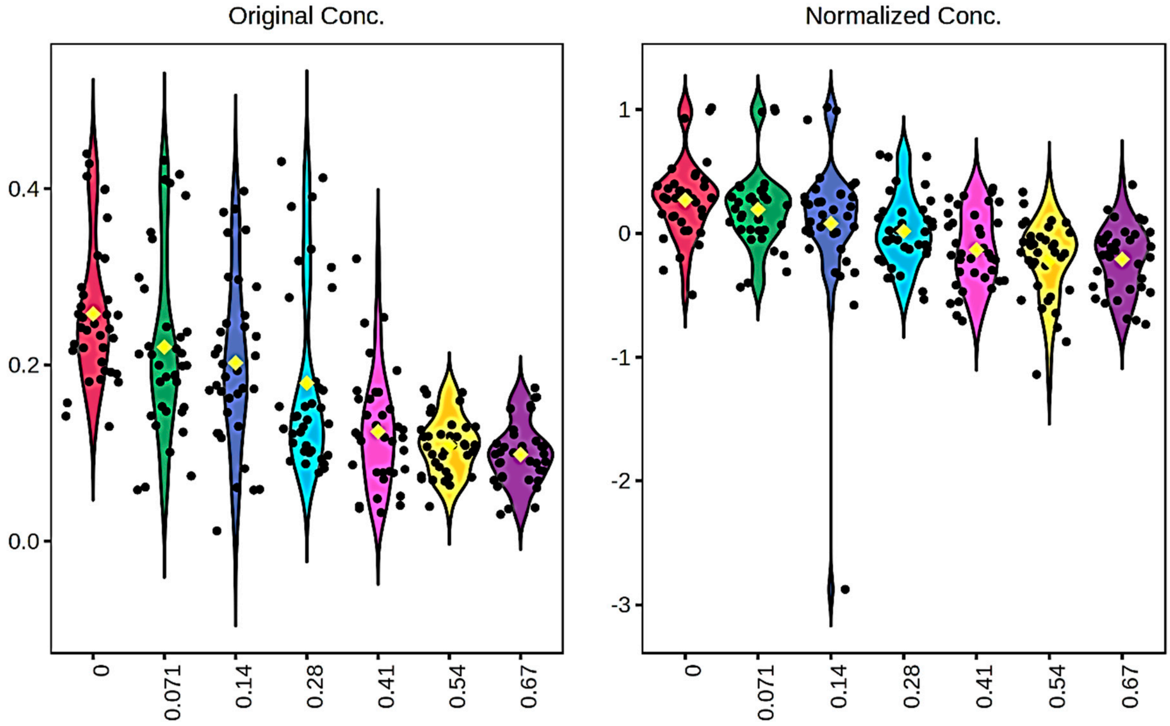

[4.12 .. 4.16]

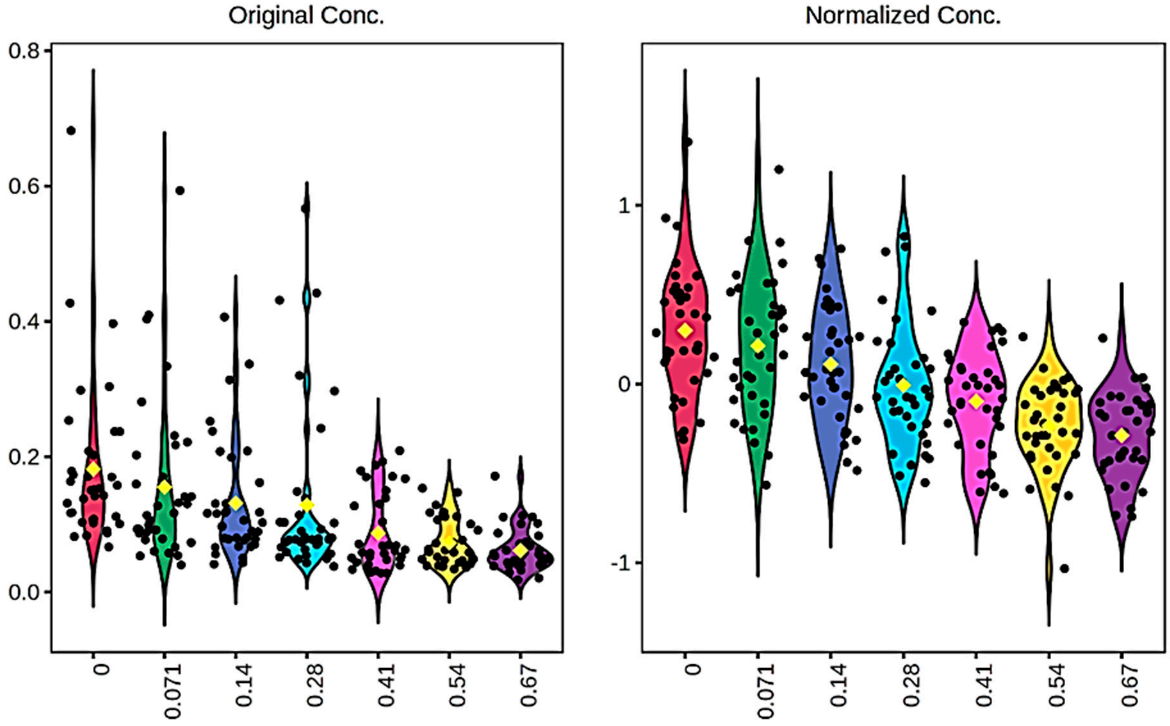

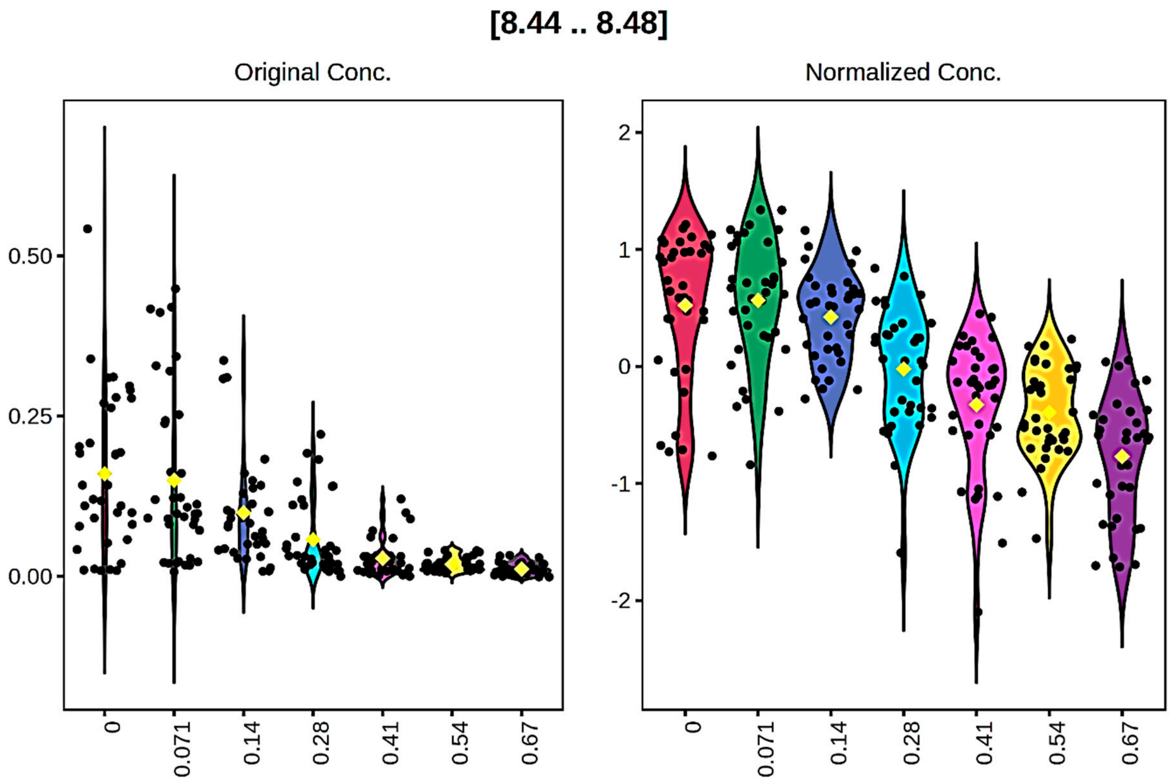

Figure S7.1 Violin plots of the spectral intensities of six of the most discriminatory ^{1}H NMR buckets obtained from application of the one-way completely randomised design ANOVA model (model 1). These plots are shown for both original and 'normalised' concentrations, the latter being PQN-normalised, glog-transformed and Pareto-scaled data. Results acquired clearly show significantly decreased intensities for some of the most statistically significant fixed ^{1}H NMR-bucketed biomolecule resonances, i.e. [0.92..0.96] ppm (leucine-C$\underline{H}_3$/isoleucine-C$\underline{H}_3$); [1.32..1.36] and [4.12..4.16] ppm (lactate-C$\underline{H}_3$ and -C$\underline{H}$ respectively); [1.92..1.96] ppm (acetate-C$\underline{H}_3$); [2.40..2.44] ppm (succinate-C$\underline{H}_2$s); and [8.44..8.48] ppm (formate-$\underline{H}$) with increasing concentrations of added Ni(II) (abscissa axis, 0.071-0.67 mmol/L). The yellow points represent median values, and the 'violin' widths indicate 'between-participant' datapoint frequencies at differing added Ni(II) levels, original or normalised.

The added Ni(II) concentration at which its effect on resonance intensities commenced varied significantly between the fixed metabolite buckets featured. For example, that observed for succinate occurred from the first 0-71 μmol/L treatment, whereas those for lactate (-CH_3 signal), formate and acetate did not commence until at or after added concentrations of 71, 140 and 280 μmol/L Ni(II) respectively; these results differ somewhat from those shown in Table 1 in view of difficulties experienced with directly viewing these changes spectro-visually, specifically complications with monitoring resonance line-shapes, amplitudes, and even their frequencies manually. Further detailed information on these results and the classification of Ni(II) complexants is available below and in Section 3.6.3.

From these violin plots, particularly notable was the observation that the median values of a series of PQN-normalised signals increased in intensity with increasing added Ni(II) level, whereas corresponding plots for the raw data generally only revealed decreases in these values (these observations were predominantly visible in corresponding plots excluded from Figure S3). This is

ascribable to the normalization approach taken, and that the PQN intensities of these resonances can elevate from higher Ni(II) concentration-induced resonance decreases occurring elsewhere in the spectrum, i.e. those which very significantly broaden or are even eliminated, such as the those of the powerful Ni(II) complexants formate, lactate and acetate, the latter of which accounts for an average value of >30% of the total spectral profile intensity. Indeed, overall this normalization process is based on a maximal dilution probability, which is estimated via computation of the most probable quotient between evaluated and reference spectra, in this case those of the added concentration-dependent Ni(II)-treated and untreated (zero control) samples respectively (this PQN approach involves consideration of the median values of many such estimates as a normalization standard, rather than a single total resonance sum as used in constant sum normalization (CSN)) [30]. Similarly, a significant number of metabolite buckets from study datasets which were CSN- and not PQN-normalised were also found to increase rather than decrease in response to added Ni(II) ion (also with subsequent glog-transformation and Pareto scaling) before statistical analysis. For this reason, we explored the classification of these added Ni(II)-responsive resonances and their assignments, in CSN-normalised datasets, into a total of four different categories (classes 1-4), and this is described in detail in Section S8 below. These four classes were found to determine both the nature and Ni(II) ion dependence of changes observed in WMSS sample ^{1}H NMR signals, and in particular they provided an estimation of the differing added Ni(II) concentrations at which they were found to commence for each salivary bucket analyte evaluated.

Notably, the Figure S7.1 plots also indicate the differing responses of participants to added Ni(II) in the context of metabolic responses of their saliva samples to this treatment. For example, at added levels of 280 μmol/L or above, there appears to be bi- or even trimodal 'between-participant' responses to added Ni(II) treatment for some of the buckets, most especially at the higher levels evaluated.

Therefore, the above results indicate the relative orders of Ni(II) complexation of added Ni(II) by WMSS ligands, although clearly this bioanalytical approach offers more relevant detail than those observed via direct visual inspection, most notably because at least some of these broadenings occur for resonances which are located in crowded spectral regions, and therefore are less easy to observe and decipher by the simpler of the two methods. It was, however, found that there was some reasonable agreement between the above ANOVA- and violin plot-based Ni(II)-complexing order and that found for direct visual inspection, although the computerised statistical analysis methods provided further valuable information and insight regarding the featuring of amino acid resonance intensity changes which were not so easily inspection-visible, notably at the lower added levels. Moreover, as noted above, signal modifications occurring in 'crowded' spectral regions were more easily decipherable via linked metabolomics analysis.

Section S8: Segregation of fixed ^{1}H NMR metabolite buckets according to their concentration-dependent responses to added Ni(II) ion

(1) Performance of two-comparator pairwise PLS-DA models to assess differences between the ^{1}H NMR profiles of WMSS samples obtained at each added Ni(II) level and that of the untreated zero control group.

As noted above from Figure S7.1, there was a significant concentration-based lag-phase observed for the most important Ni(II)-complexing carboxylato ligands present in WMSS samples (i.e., for acetate and lactate, in addition to formate), in which there was little of no added Ni(II)-induced change to their resonance patterns at levels of up to 71-280 μmol./L (Figures S6.1 and S7.1). Indeed, Table S1 shows the variable importance parameter (VIP) values for a series of two-comparator PLS-DA models which directly evaluated modifications to the ^{1}H NMR spectral profiles of untreated WMSS samples against each Ni(II) level investigated individually (values <1 are not considered to be significant). Table S1 also reveals that the above Ni(II) complexants only dominate the complexation order at its higher added concentrations, although the sequential order of resonance changes observed was formate > lactate ≈ succinate > acetate within the 0.28 to 0.67 mmol/L added Ni(II) level range. Although it appears that lactate and succinate have a similar affinity for added Ni(II) ion under our experimental conditions, the much higher WMSS level of the former ligand over the latter over-compensates for the known higher stability constants for succinato-Ni(II) complexes (Section 4.1, *oc cit*). As expected, model Q^2 cross-validation values increased from 0.62 to 0.89 as the added Ni(II) concentration is raised from 0.071 to 0.67 mmol/L Ni(II) (validating permutation tests for all these PLS-DA comparisons were all highly significant (p <5 x 10^{-4})).

Table S1. VIPs and rank orders of key carboxylate anion Ni(II) complexants for PLS-DA models which directly compared Ni(II)-induced modifications to the ^{1}H NMR spectra of untreated WMSS samples against those for each Ni(II) level investigated individually, i.e. two-comparator systems only. Model cross-validating Q^2 and permutation test p values are also listed. Datasets were PQN-normalised, glog-transformed and Pareto-scaled prior to analysis.

**Biomolecule**	**Added [Ni(II)]**					
	**0.071**	**0.140**	**0.280**	**0.410**	**0.540**	**0.670**
**Acetate-CH_3 (rank)**	<1	<1	<1	1.40 (12)	1.92 (5)	2.46 (2)
**Formate-H (rank)**	<1	1.20 (20)	3.87 (1)	4.34 (1)	4.15 (1)	4.63 (1)
**Lactate-CH_3 (rank)**	<1	<1	1.50 (11)	1.76 (5)	2.04 (3)	2.40 (3)
**Lactate-CH (rank)**	<1	1.41 (14)	1.65 (7)	1.80 (4)	2.00 (4)	2.02 (4)
**Succinate-$(CH_2)_2$ (rank)**	<1	<1	1.60 (8)	1.97 (2)	1.88 (7)	1.74 (6)
**Maximum Q^2 (minimum no. of components)**	0.62 (8)	0.77 (8)	0.84 (6)	0.83 (6)	0.82 (6)	0.89 (5)
**Permutation test *p* value**	<5 x 10^{-4}	<5 x 10^{-4}	<5 x 10^{-4}	<5 x 10^{-4}	<5 x 10^{-4}	<5 x 10^{-4}

In order to further clarify the 'early' stage of Ni(II) complexation or chelation, which mainly involves amino acids and amines, etc. (i.e., class 4 as defined below), we then elected to explore these processes at its lowest added concentration (0.071 mmol/L) in some detail, and for this purpose PLS-DA two-comparator models were constructed for evaluating differences between the ^{1}H NMR profiles of 0.071 mmol/L Ni(II)-treated WMSS samples and those of the untreated control cohort; corresponding dual comparisons between the 0.14 *versus* 0.00, the 0.28 *versus* 0.00, and the 0.41 *versus* 0.00 added Ni(II) levels were also made using this methodology. For the first (0.071 mmol/L) comparison, corresponding PLS-DA variable importance parameter (VIP) values (in brackets) for the most important Ni(II)-induced changes to biomolecule signals found were N-acetylneuraminate/ornithine (2.70) > methylamine (2.48) > tentatively β-glucose (2.48 and 1.63) > histidine (2.36) > thymine (2.14) > dimethylglycine (1.93) > ethanolamine (3.16) > taurine (1.73 and 1.64), i.e., this order contains many more N-donor ligands than those discovered at the higher added Ni(II) concentrations, and this is of much importance regarding the known estimated salivary levels of trace Ni(II), which are at least 2-3 orders of magnitude lower than the smallest one involved here [21].

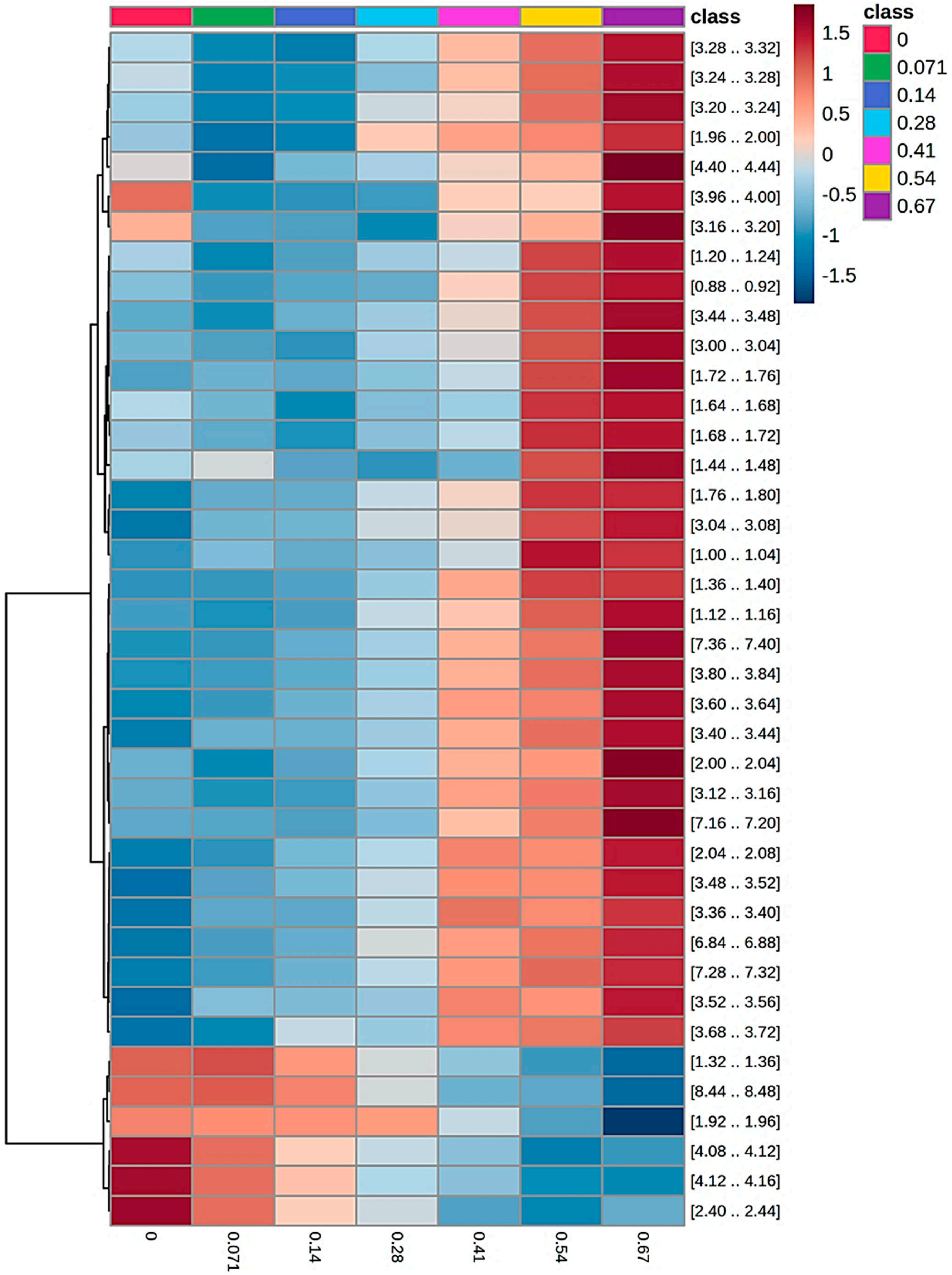
class
1.5
1
0.5
0
-0.5
-1
-1.5
class
0
0.071
0.14
0.28
0.41
0.54
0.67
[3.28 .. 3.32]
[3.24 .. 3.28]
[3.20 .. 3.24]
[1.96 .. 2.00]
[4.40 .. 4.44]
[3.96 .. 4.00]
[3.16 .. 3.20]
[1.20 .. 1.24]
[0.88 .. 0.92]
[3.44 .. 3.48]
[3.00 .. 3.04]
[1.72 .. 1.76]
[1.64 .. 1.68]
[1.68 .. 1.72]
[1.44 .. 1.48]
[1.76 .. 1.80]
[3.04 .. 3.08]
[1.00 .. 1.04]
[1.36 .. 1.40]
[1.12 .. 1.16]
[7.36 .. 7.40]
[3.80 .. 3.84]
[3.60 .. 3.64]
[3.40 .. 3.44]
[2.00 .. 2.04]
[3.12 .. 3.16]
[7.16 .. 7.20]
[2.04 .. 2.08]
[3.48 .. 3.52]
[3.36 .. 3.40]
[6.84 .. 6.88]
[7.28 .. 7.32]
[3.52 .. 3.56]
[3.68 .. 3.72]
[1.32 .. 1.36]
[8.44 .. 8.48]
[1.92 .. 1.96]
[4.08 .. 4.12]
[4.12 .. 4.16]
[2.40 .. 2.44]
0
0.071
0.14
0.28
0.41
0.54
0.67

Figure S8.1 Agglomerative Hierarchical Clustering (AHC)-supported ANOVA-based heatmap showing the top 40 bucket predictor variables based on the antecedent of their mean values, showing decreases or increases in their intensities with increasing added Ni(II) levels. The colour code keys provided in the top right-hand side of the diagram shown depict those for increasing added Ni(II) concentrations (0.00-0.67 mmol/L). ^{1}H NMR bucket intensities (in ppm on the ordinate axis) are plotted against the added Ni(II) level variable (abscissa axis). Datasets were constant sum-normalised, glog-transformed and Pareto-scaled prior to analysis.

(2) AHC analysis and facilitated heatmap construction for the whole spectral range to segregate responding biomolecular WMSS complexants into four different added Ni(II) ion concentration-responding classes.

AHC analysis of ^{1}H NMR bucket 'predictor' variables displayed in this heatmap (Figure S8.1, left-hand side ordinate axis) revealed that these formed four separate major cluster classifications, i.e. classes 1-4. Fixed 0.04 ppm buckets within these classes, and their ^{1}H NMR assignments, are provided below.

Class 1 Bucket Biomolecule Assignments: [0.92..0.96], leucine-C$\underline{H}_3$/isoleucine-C$\underline{H}_3$; [1.32..1.36], lactate-C$\underline{H}_3$; [8.44..8.48], formate-$\underline{H}$; [1.92..1.96], acetate-C$\underline{H}_3$; [4.12..4.16], lactate-C$\underline{H}$; [2.40..2.44] ppm, succinate-C$\underline{H}_2$s; and [4.08..4.12], lactate-C$\underline{H}$.
Class 2 Bucket Biomolecule Assignments: [3.68..3.72], 1-methylhistidine-N1(C$\underline{H}_3$)/3-methylhistidine-N3(C$\underline{H}_3$)/lysine-α-C$\underline{H}$; [3.52..3.56], glycine-α-C$\underline{H}_2$/threonine-α-C$\underline{H}$/valine-α-C$\underline{H}$; [7.28..7.32], phenylalanine-C2$\underline{H}$/C6$\underline{H}$; [6.84..6.88], 4-hydroxyphenylacetate-C3$\underline{H}$/C5$\underline{H}$ aromatic ring protons; [3.36..3.40], methanol-C$\underline{H}_3$; [3.48..3.52], phenylacetate-C$\underline{H}_2$; [2.04..2.08], GlycA /low-molecular-mass N-acetyl transfer agents-NHCOC$\underline{H}_3$; [7.16..7.20], 4-hydroxyphenylacetate aromatic ring-C2$\underline{H}$/C6$\underline{H}$; [3.12..3.16], dimethylsulphone-SO(C$\underline{H}_3$)$_2$/1/2 phenylalanine-β-C$\underline{H}_2$; [2.00..2.04], GlycA /low-molecular-mass N-acetylsugar-NHCOC$\underline{H}_3$; [3.40..3.44], taurine-C$\underline{H}_2$SO$_3^-$/carnitine-β-C$\underline{H}_2$/proline-α-C$\underline{H}$; [3.60..3.64], propane-1,3-diol-C$\underline{H}_2$OH/β-fucose-C2$\underline{H}$; [3.80..3.84], ethanolamine-C$\underline{H}_2$OH; [7.36..7.40], phenylalanine aromatic ring-C4$\underline{H}$; [1.12..1.16], 3-AIB-C$\underline{H}_3$; and [1.36..1.38], acetoin-C$\underline{H}_3$.
Class 3 Bucket Biomolecule Assignments: [1.00-1.04], valine-C$\underline{H}_3$; [3.04..3.08], creatine-N(C$\underline{H}_3$)/putrescine-α-C$\underline{H}_2$; [1.76..1.80], leucine-β-C$\underline{H}_2$; [1.44..1.48], alanine-C$\underline{H}_3$; [1.68..1.72], leucine-γ-C$\underline{H}_2$/lysine-δ-C$\underline{H}_2$/arginine-γ-C$\underline{H}_2$/putrescine-β-C$\underline{H}_2$; [1.64..1.68], 5-aminovalerate-3/4-C$\underline{H}_2$s/ leucine-γ-C$\underline{H}_2$; [1.72..1.76], lysine-δ-C$\underline{H}_2$; [3.00..3.04], creatinine-N(C$\underline{H}_3$)/GABA-γ-C$\underline{H}_2$/lysine-ε-C$\underline{H}_2$/5-aminovalerate-δ-C$\underline{H}_2$; [3.44..3.48], β-fucose-C2$\underline{H}$; [0.88..0.92], *n*-butyrate-C$\underline{H}_3$; and [1.20..1.24], 3-D-hydroxybutyrate-C$\underline{H}_3$.
Class 4 Bucket Biomolecule Assignments: [3.16..3.20], ethanolamine-C$\underline{H}_2$NH$_2$/choline-N(C$\underline{H}_3$)$_3$; [3.96..4.00], phosphocreatine-C$\underline{H}_2$/histidine-α-C$\underline{H}$; [4.40..4.44], dihydroxyacetone-C$\underline{H}_3$s/broad macromolecule resonance protons; [1.96..2.00], 2-hydroxyglutarate-γ-C$\underline{H}_2$/proline-γ-C$\underline{H}_2$; [3.20..3.24], taurine-C$\underline{H}_2$NH$_2$/histidine-β-C$\underline{H}_2$; [3.24..3.28], taurine-C$\underline{H}_2$NH$_2$/histidine-β-C$\underline{H}_2$/trimethylamine-N-oxide (TMAO)-N(C$\underline{H}_3$)$_3$; and [3.28..3.32], 1/2 phenylalanine-/tryptophan-β-C$\underline{H}_2$.
Abbreviations: AIB, 3-aminoisobutyrate; GABA, γ-aminobutyrate.

Plots of mean±95% CIs for the normalised mean intensities of all metabolites located within these clusters *versus* added Ni(II) level are shown in Figure S8.2(a)-(d), and these clearly show that cluster 1 comprises all the major organic acid anion complexants, which depicts a 'lag' Ni(II) concentration phase before significantly decreasing at higher added levels in a sequential manner. In contrast, cluster 4 demonstrates a prior significant Ni(II)-induced complexation decrease at 0.07-0.14 mmol/L added

levels of this metal ion, and then steadily increases from 0.14-0.28 to 0.67 mmol/L Ni(II), a consequence of the marked spectral consumption of class 1 ligands and their ^{1}H NMR resonances. Class 4 contains some key Ni(II)-complexing amino acids such as proline, histidine and taurine, together with ethanolamine and creatines, although assignments for their buckets overlapped significantly, and this complicated the analysis performed. Corresponding plots for classes 2 and 3 showed rises in normalised mean intensities with increasing Ni(II) concentration, the latter not commencing until after 0.14 mmol/L added Ni(II). The small, albeit reproducible decrease in intensity from added Ni(II) levels of 0.00 to 0.14 mmol/L observed for class 3 biomolecules presumably arises from resonance broadenings, which in turn reflect a relatively low level of Ni(II) complexation. Descriptions of the biomolecular constituents of classes 1-4 are provided below.

Class 1: The first of these classes (class 1) consisted of six of the top, most discriminatory buckets discovered, which were predominantly ascribable to the carboxylate-O-donor ligands lactate, formate, acetate and succinate, and for the first three or four of these there was a Ni(II) concentration-based lag phase for intensity reductions (from 0.00-0.14 mmol/L for lactate and formate, 0.00-0.28 mmol/L for acetate, but only 0.00-0.071 mmol/L for succinate), which then all sequentially progressed to much reduced levels up to a final added value of 0.67 mmol/L Ni(II) (Figure S8.2(a)). However, in view of the marked differences found in the response of WMSS ^{1}H NMR signals to Ni(II) added at its lowest level, it is important to add that this 'top 6' description only applies to the total metabolite concentrations consumed via complexation reactions in total across all concentrations studied (up to the highest added level of 0.67 mmol/L). Moreover, one further source of this correlated class of mainly carboxylic acid anions is their predominantly oral microbial source, as demonstrated in Ref. [25], although it should be noted that this may only be the case for the untreated samples shown, and not those resulting from Ni(II) titration.

Class 2: The second cluster class of resonances affected by Ni(II) addition encompassed 16 buckets assigned to an almost equivalent number of metabolites, and which in terms of the CSN process applied, sequentially increased in intensity from added Ni(II) concentrations of 0.00 to 0.67 mmol/L, the starting-point being low 'normalized-equivalent' concentrations. Assignments for these buckets covered a range of amino acids, both aliphatic and aromatic, dimethylsulphone, phenylacetate, 4-hydroxyphenylacetate and acetoin. This increase certainly appears to arise from the important Ni(II)-induced consumption of carboxylate anion donor metabolites in cluster pattern 1, which occurs following a metabolite-dependent lag concentration phase for these biomolecules (Figure S4). Notably, in untreated samples, the percentage spectral contributions of carboxylate anion resonances towards the total CSN dataset were lactate (2.04%), formate (6.30%), succinate (1.15%) and acetate (as much as 21.89%), i.e. a total of more than 30% respectively, and major decreases in the intensities of these species were observable from added Ni(II) levels of 0.28 or 0.41 mmol/L. Consequently, class 2 metabolite signals were inflated to a large extent, and this sequential increase was clearly dependent on increasing added Ni(II) concentration (Figure S8.2(b)). However, since no paramagnetically-induced decrease in bucket intensities was observed, this dependence indicated only a limited participation of class 2 metabolites in complex formation, or alternative interactions, with added Ni(II) ions.

Class 3: In addition to creatine/putrescine, n-butyrate, 5-aminovalerate, β-fucose, dimethylsulphone and 3-D-hydroxybutyrate, the class 3 cluster pattern contains a range of low salivary concentration amino acids which also have quite powerful affinities for and form known complexes with Ni(II), for

example alanine, arginine and lysine (11 metabolites in total). This titration curve featured a minor decrease in normalized intensity from 0.00-0.28 mmol/L, which then increased in an S-shaped (sigmoidal) fashion to reach a maximum at 0.67 mmol/L added Ni(II) (Figure S8.2(c)). This initial decrease in mean intensity value confirmed the Ni(II)-induced paramagnetic broadenings of the above biomolecules prior to influence of the increasing relative proportionate contributions of these and all other 'surviving' ^{1}H NMR WMSS metabolite signals, which was found to increase across class 3 biomolecules from the addition of 0.28 mmol/L Ni(II).

Visual inspection of spectra acquired at added Ni(II) levels of 0.41-0.67 mmol/L revealed that resonances with intensities increased over those of the untreated control samples in classes 2 and 3 were those which were still sharper and hence more visible, i.e. they were broadened less so than those of others, an observation indicating that their affinities for Ni(II) were low or limited. An additional complication is the 'spreading' of broadened adjacent resonances into those with fixed 0.04 ppm buckets in these groups.

Class 4: The fourth class of Ni(II) ion responders, however, featured biomolecules with resonance buckets reflecting only low salivary concentrations for them (seven in total), mainly free amino acids such as histidine, taurine and phenylalanine, along with ethanolamine and phosphocreatine, which significantly diminished in intensity within the 0.071-0.14 mmol/L Ni(II) range, but following the addition of 0.28 mmol/L Ni(II), then began to increase quite substantially over and above their original (Pareto-scaled) logarithmic ^{1}H NMR intensities (and which are proportional to CSN-proportionate concentrations) at the highest values (Figure S8.2(d)). This is attributable to their prior complexation or interaction with added Ni(II) (reductions in resonance intensities) at the early titration points, followed by normalized enhancements in these parameters in view of the substantial consumption of cluster pattern 1 organic acid anion complexant contributors to total spectral signal intensities described above, as expected. Therefore, this class should be considered the most important one for Ni(II) speciation studies at the lowest added concentration evaluated. Nevertheless, the assignments of resonances in the quite crowded spectral region involved for this class (mainly 3.00-4.50 ppm) are rendered very complex in view of the superimposition of two or more resonances within each fixed 0.04 ppm bucket discovered for it. However, class 4 is the only cluster pattern which clearly demonstrates highly statistically significant reductions in resonance intensities at the lowest added Ni(II) level, and therefore further experimental strategies were adopted to further explore significant complexants for this metal ion at the lowest concentration tested. Such investigations (as described in Section 3.6.3 of the main text) also have the ability to distinguish between constituents of this class and the pattern described by class 1 above.

CIs (95%) calculated for each individual metabolite buckets at all added Ni(II) concentration additions, and across all participants and replicate samples tested, were used to confirm the elimination of many of them from these Ni(II) response classifications, notably those which did not appear in the four cluster sets depicted in Figure S8.1 with buckets elsewhere in the ^{1}H NMR profiles acquired, and which had substantially overlapping CIs throughout the entire spectral titration range used.

Similar observations were made when using the PQN row-wise normalization approach, and this is to be expected since all dataset buckets are normalized to a median spectral profile computed from all non-Ni(II)-treated zero control samples [41]. Since this median profile remains constant and unchanged

throughout the whole analysis procedure, relatively large decreases in the intensities of the above class 1 carboxylato ligands induced by added Ni(II) will be expected to result in net increases in those of the remaining classes, most especially for biomolecules which have much lower salivary concentrations than those present in class 1. Indeed, class 4 amino acids and amines, etc. become saturated at early stages of the Ni(II) titration (i.e., highly significant decreases in intensity observed ascribable to resonance broadenings), but this is then followed by relatively large increases in this parameter from added Ni(II) levels of 0.14-0.28 mmol/L. Alternatively, if they have no or only limited affinities for this metal ion (as in class 2 metabolites), their relative intensities will be expected to increase with increasing Ni(II) level at lower added concentrations in view of the intensity decreases observed for resonances of the high concentration Ni(II)-complexing class 1 biomolecules.

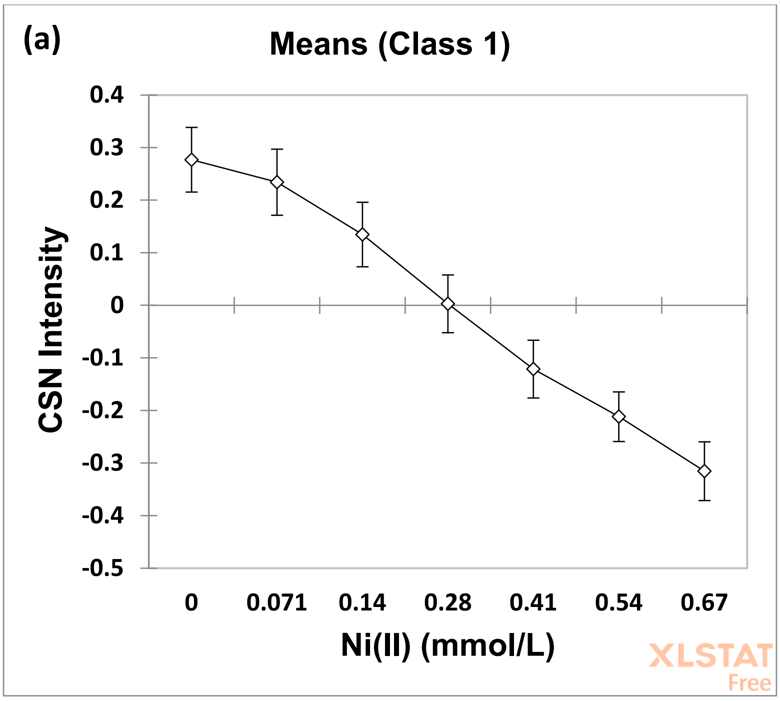

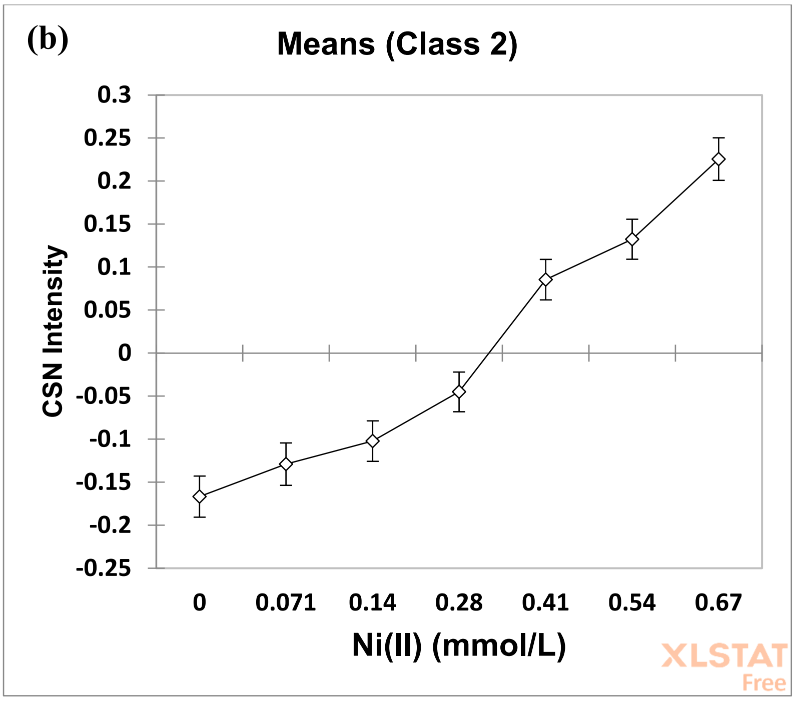
(b)
Means (Class 2)
0.3
0.25
0.2
0.15
0.1
0.05
0
-0.05
-0.1
-0.15
-0.2
-0.25
CSN Intensity
0
0.071
0.14
0.28
0.41
0.54
0.67
Ni(II) (mmol/L)
XLSTAT
Free

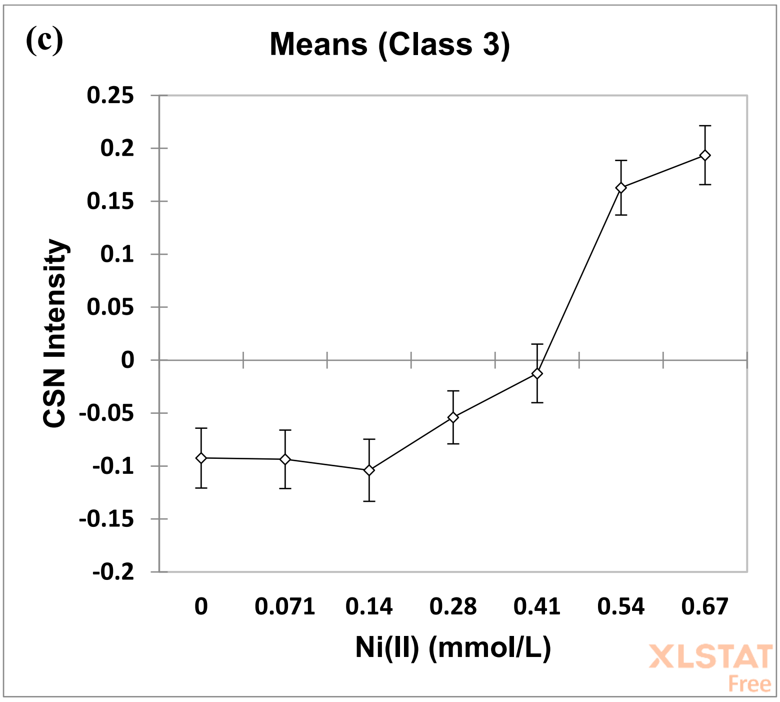
(c)
Means (Class 3)
0.25
0.2
0.15
0.1
0.05
0
-0.05
-0.1
-0.15
-0.2
CSN Intensity
0
0.071
0.14
0.28
0.41
0.54
0.67
Ni(II) (mmol/L)
XLSTAT
Free

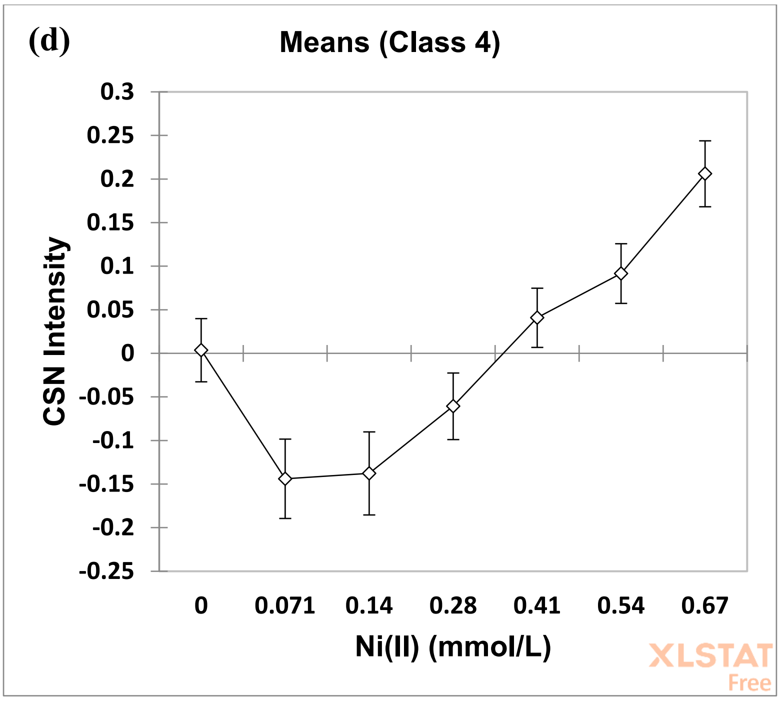
(d)
Means (Class 4)
CSN Intensity
0.3
0.25
0.2
0.15
0.1
0.05
0
-0.05
-0.1
-0.15
-0.2
-0.25
0 0.071 0.14 0.28 0.41 0.54 0.67
Ni(II) (mmol/L)
XLSTAT Free

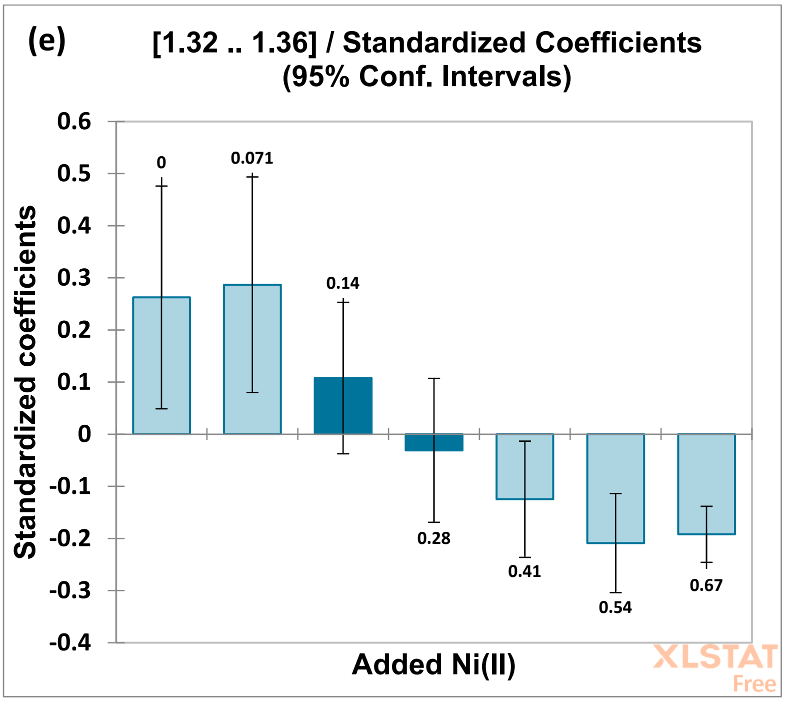
(e)
[1.32 .. 1.36] / Standardized Coefficients
(95% Conf. Intervals)
Standardized coefficients
0.6
0.5
0.4
0.3
0.2
0.1
0
-0.1
-0.2
-0.3
-0.4
0
0.071
0.14
0.28
0.41
0.54
0.67
Added Ni(II)
XLSTAT Free

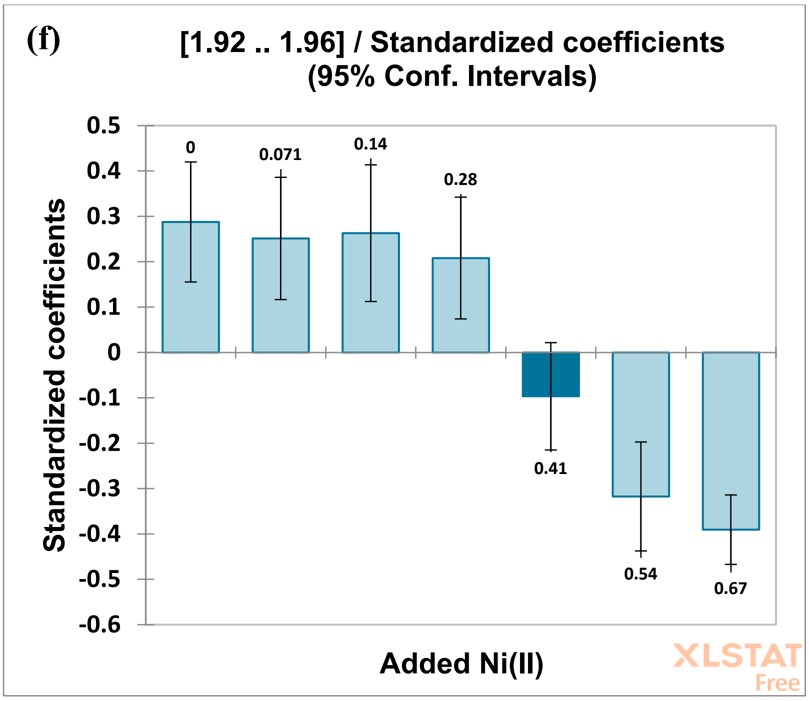
(f)
[1.92 .. 1.96] / Standardized coefficients
(95% Conf. Intervals)
Standardized coefficients
0.5
0.4
0.3
0.2
0.1
0
-0.1
-0.2
-0.3
-0.4
-0.5
-0.6
0
0.071
0.14
0.28
0.41
0.54
0.67
Added Ni(II)
XLSTAT
Free

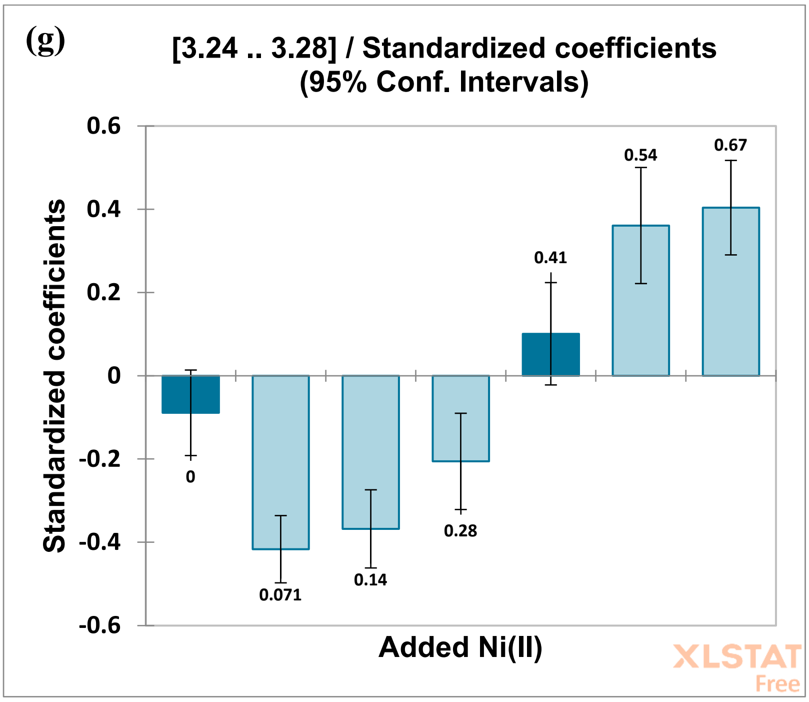
(g)
[3.24 .. 3.28] / Standardized coefficients
(95% Conf. Intervals)
Standardized coefficients
0.6
0.4
0.2
0
-0.2
-0.4
-0.6
0
0.071
0.14
0.28
0.41
0.54
0.67
Added Ni(II)
XLSTAT
Free

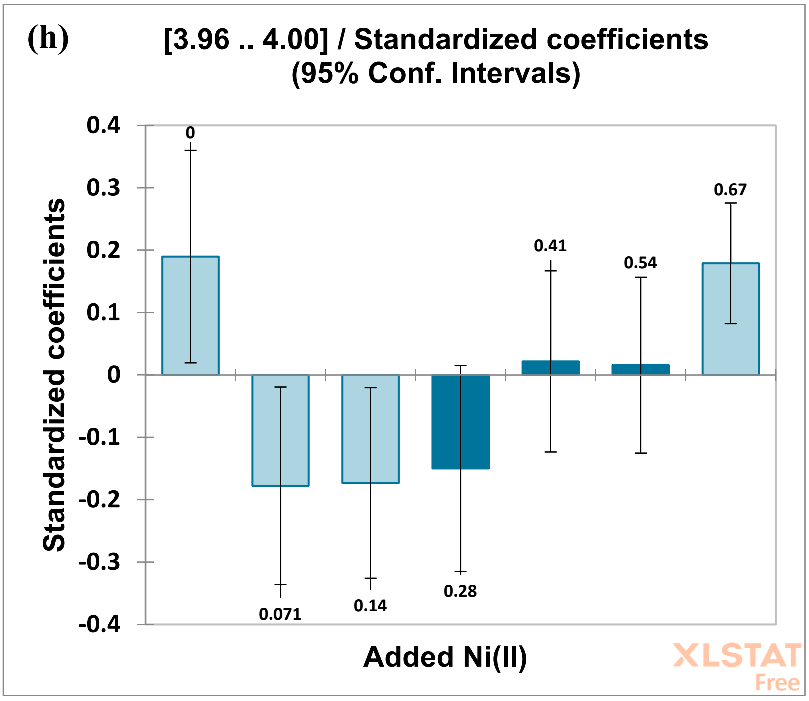

Figure S8.2 (a), (b), (c) and (d), Plots of mean±95% CI CSN intensity values for metabolites in Ni(II)-responsive classes 1, 2, 3 and 4 respectively *versus* added Ni(II) concentrations for WMSS datasets which were constant sum normalised, glog-transformed and Pareto-scaled (classes 1, 2, 3 and 4 contained 6, 16, 11 and 7 biomolecule signal buckets respectively). The 95% CI values included sources of variation arising from the 'between-participants' and the 'between-added Ni(II) concentrations' x 'between-participants' interaction effect (i.e., according to model 1), and therefore these are much wider than those without these variance contributors considered and removed. (e), (f), (g) and (h), Added Ni(II) ion-dependent changes in standardised coefficients arising from the performance of an ANOVA model 1 analysis to individual metabolite buckets ([1.32..1.36], [1.92..1.96], [3.24..3.28] and [3.96..4.00] ppm) corresponding to the lactate-C$\underline{H}_3$, acetate-C$\underline{H}_3$, taurine-C$\underline{H}_2$NH_2 and phosphocreatine-C$\underline{H}_2$ resonances respectively). From the Pareto-scaling applied, the overall mean value of all entries in all of the above plots is zero.

Individual ANOVA model 1 standardised coefficient *versus* added Ni(II) level plots for four of the biomolecules contributing towards cluster pattern classes, with two each of them contributing towards both added Ni(II) responder classes 1 and 4, are shown in Figures S8.2(e)-(h). Figures 8.25(e) and (f) show Ni(II) concentration lag-phases prior to marked decreases in plots for the intensities of the individual lactate- and acetate-CH_3 resonances respectively, a characteristic of class 1 respondent buckets. Furthermore, Figures S8.2(g) and (h) clearly show prior reductions in the normalised intensities of signals assigned to the class 4 taurine-C$\underline{H}_2$NH_3^+ and phosphocreatine-C$\underline{H}_2$ protons, which occur within the 0.00-0.28 mmol/L added Ni(II) concentration range; this serves as a requirement for this class of Ni(II)-complexing biomolecules.

Since the CSN and PQN normalization methods applied introduce spectral complications at high added Ni(II) levels, in which the Ni(II) complexation-induced loss of originally quite intense complexant ligand resonances, particularly those of acetate and further carboxylate anions, gives rise to enhanced normalized intensities of other less competing biomolecule resonances, a further ANOVA-based heatmap was generated, which featured added Ni(II) levels up to a threshold limit of 0.28

mmol/L only. From this, the order of resonance responsiveness (significance) was tyrosine ≈ formate ≈ taurine ≈ ethanolamine ≈ dimethylamine > 4-hydroxyphenylacetate ≈ lactate ≈ ethanol ≈ succinate ≈ *n*-butyrate ≈ creatine ≈ histidine > lactate ≈ acetoacetate ≈ acetone ≈ tentative carbohydrate anomeric proton signal (δ = 4.52 ppm (*d*)) assignment > 3-AIB (data not shown).

A further heatmap for a model developed with only added Ni(II) concentrations incorporated as the 'predictor' variables (individual participant values were averaged), and with AHC clustering applied to both ^{1}H NMR buckets and added Ni(II) levels, was then developed. Notably, this technique clustered together the 0 and 71, the 140 and 280, the 410 alone, and the 540 and 670 μmol/L added Ni(II) concentration groups (data not shown). Hence, these clusterings proceeded sequentially, with similarities being found between the above pairs of Ni(II) levels added, although the group of samples treated with 410 μmol/L Ni(II) appeared to be distinctive from all other concentration groups studied.

(3) Application of paired sample t-tests, which avoid the complication of interferences from the 'between-participant' component of variance from the model, to sequentially evaluate the effects of increasing added Ni(II) ion concentrations on the ^{1}H NMR profiles of WMSS samples.

From this analysis, Ni(II)-complexing metabolite order rankings (Bonferroni-corrected *p* value for statistical significance in brackets) were: taurine-$C\underline{H}_2SO_3^-$ (1.32 x 10^{-5}) > taurine-$C\underline{H}_2NH_2$/histidine-β-$C\underline{H}_2$ (8.85 x 10^{-5}) > ethanolamine-$C\underline{H}_2NH_2$ (1.81 x 10^{-4}) > creatine-$C\underline{H}_2$/histidine-*α*-C$\underline{H}$ (2.81 x 10^{-4}) > propionate-$C\underline{H}_3$ (7.84 x 10^{-4}) = dimethylamine (DMA)-$N(C\underline{H}_3)_2$ (7.84 x 10^{-4}) at an added Ni(II) level of 0.071 mmol/L; lactate-C$\underline{H}$ (3.73 x 10^{-6}) > lactate-C$\underline{H}$ (4.15 x 10^{-5}) > lysine-*α*-C$\underline{H}$/1- and 3-methylhistidine-$N(C\underline{H}_3)$ (4.15 x 10^{-5}) = taurine-$CH_2SO_3^-$ (4.15 x 10^{-5}) > ethanolamine-$C\underline{H}_2NH_2$ (5.95 x 10^{-5}) > glutamate-γ-CH_2 (7.02 x 10^{-5}) at 0.14 mmol/L added Ni(II); glutamate-γ-$C\underline{H}_2$ (8.56 x 10^{-9}) > lactate-C$\underline{H}$ (8.56 x 10^{-9}) > lactate-C$\underline{H}$ (8.56 x 10^{-9}) > lactate-$C\underline{H}_3$ (3.82 x 10^{-7}) > succinate-CH_2s (4.07 x 10^{-7}) > taurine-$CH_2SO_3^-$ (1.15 x 10^{-6}) at 0.28 mmol./L added Ni(II); lactate-C$\underline{H}$ (3.37 x 10^{-12}) = lactate-C$\underline{H}$ (3.37 x 10^{-12}) > succinate-$C\underline{H}_2$s (8.30 x 10^{-11}) > lactate-$C\underline{H}_3$ (1.27 x 10^{-9}) > taurine-$CH_2SO_3^-$ (3.30 x 10^{-9}) > acetate-$C\underline{H}_3$ (9.14 x 10^{-8}) at an added Ni(II) level of 0.41 mmol./L; taurine-$C\underline{H}_2SO_3^-$ (5.85 x 10^{-13}) > lactate-$C\underline{H}_3$ (7.61 x 10^{-12}) > acetate-$C\underline{H}_3$ (1.83 x 10^{-11}) > lactate-C$\underline{H}$ (3.31 x 10^{-11}) > DMA-$N(C\underline{H}_3)_2$ (5.62 x 10^{-11}) > glutamate-γ-$C\underline{H}_2$ (5.94 x 10^{-11}) at an added Ni(II) level of 0.54 mmol./L; and taurine-$C\underline{H}_2SO_3^-$ (5.19 x 10^{-16}) > acetate-$C\underline{H}_3$ (5.59 x 10^{-15}) > lactate-$C\underline{H}_3$ (1.12 x 10^{-15}) > lactate-C$\underline{H}$ (1.81 x 10^{-14}) > formate-$\underline{H}$ (1.30 x 10^{-11}) > lactate-C$\underline{H}$ (1.00 x 10^{-10}) at an added Ni(II) level of 0.67 mmol/L. Resonances appearing twice within the same sequence are those which are split between two 0.04 ppm buckets, e.g., that of lactate-C$\underline{H}$.

(4) Comparative evaluations of ^{1}H NMR resonance intensities of the untreated control WMSS group with those obtained in that involving the lowest added Ni(II) level (0.071 mmol/L) were made using the TSP-normalised dataset only in order to determine any statistically significant metabolite bucket variables at a more physiologically-realistic biofluid Ni(II) concentration, and on a purely quantitative basis (again paired sample t-tests were employed for this purpose).

Since at this concentration of Ni(II) there was not a statistically significant increase observed in the $\Delta v_{1/2}$ value of the TSP internal standard ^{1}H NMR resonance (more specifically, little or no complexation of this metal ion by it), it was possible to obtain a TSP-normalised dataset for this group. and its mean resonance intensities were compared with that of a correspondingly normalised and transformed untreated control group (glog-transformation and Pareto-scaling was also applied prior to analysis).

From this study, ranked ^{1}H NMR spectral modifications induced by the addition of only 0.071 mmol/L Ni(II) are listed in Table S2.

Table S2. Bonferroni-corrected paired t-test significance levels for the top 20 ^{1}H NMR buckets and their resonance assignments arising from comparisons of the TSP-normalised 0.071 mmol/L Ni(II) WMSS dataset with that of the untreated control samples only. Abbreviations: DMA, dimethylamine; MA, methylamine; Cr, creatine; PCr, phosphocreatine; Cn, creatinine; DHA, dihydroxyacetone. *This spectral modification may involve a change in chemical shift value rather than a resonance broadening effect (Figure S5.3).

Rank Code	^{1}H NMR Bucket (ppm)	Assignments	Bonferroni-corrected *p* value
1	[2.72 .. 2.76]	DMA-N(C$\underline{H}_3$)$_2$	1.30 x 10^{-4}
2	[3.96 .. 4.00]	Creatine-C$\underline{H}_2$/Histidine-α-C$\underline{H}$	1.55 x 10^{-4}
3	[3.24 .. 3.28]	Taurine-CH_2NH_2/Histidine-β-CH_2/TMAO-N$(CH_3)_3$	1.64 x 10-^{4}
4	[3.12 .. 3.16]	Dimethylsulphone-CH_3s/1/2 His-/Phe-β-CH_2	7.57 x 10-^{4}
5	[3.16 .. 3.20]	Ethanolamine-C$\underline{H}_2$NH$_2$/Choline-N(C$\underline{H}_3$)$_3^+$	7.57 x 10^{-4}
6	[4.08 .. 4.12]	Lactate-C$\underline{H}$/Proline-α-C$\underline{H}$	1.17 x 10^{-3}
7	[4.00 .. 4.04]	*Cn-CH_2/PCr-C$\underline{H}_2$	1.24 x 10^{-3}
8	[2.28 .. 2.32]	Acetoacetate-CH_3	1.54 x 10^{-3}
9	[7.88 .. 7.92]	Histidine/3-Methylhistidine-imidazole ring-C2H	2.39 x 10^{-3}
10	[4.40 .. 4.44]	Broad macromolecule signal	2.86 x 10^{-3}
11	[4.12 .. 4.16]	Lactate-CH	3.54 x 10^{-3}
12	[7.20 .. 7.24]	Tyrosine aromatic ring-C3H/C5H	3.54 x 10^{-3}
13	[4.44 .. 4.48]	DHA-CH_3s/broad macromolecule signal	3.54 x 10^{-3}
14	[2.60 .. 2.64]	MA-N(CH_3)	3.54 x 10^{-3}
15	[2.32 .. 2.36]	Glutamate-γ-CH_2	5.89 x 10^{-3}
16	[2.36 .. 2.40]	Pyruvate-CH_3	6.67 x 10^{-3}
17	[3.20 .. 3.24]	Choline-N$(CH_3)_3$/TMAO-N$(CH_3)_3$	6.67 x 10^{-3}
18	[3.28 .. 3.32]	Phenylalanine-/Tryptophan-β-CH_2	6.67 x 10^{-3}
19	[2.08 .. 2.12]	Methionine-SCH_3/Dimethylsulphide-S$(CH_3)_2$	6.67 x 10^{-3}
20	[2.24 .. 2.28]	5-Aminovalerate-α-CH_2/Acetone-CH_3	6.67 x 10^{-3}

Section S9: Methods and results arising from the application of covariate- and interaction effect-balancing two-way ANOVA models (direct univariate ANOVA-based analysis)

This strategy performed covariate analysis, including n = 12 covariate ('between-participant') modifications (with n = 3 replicate analyses for each sample), and served to detect major predictive patterns regarding the two major factors considered, along with their first-order interaction effect when found to be statistically significant. Therefore, three experimental designs of increasing complexity were evaluated: Model 1 featured a single factor ANOVA design with the only source of variation considered being the 'between-added Ni(II) concentrations' fixed effect, along with that for fundamental error; model 2 was as model 1, but also including the critical 'between-participants' covariate; and model 3 was as model 2, but also including the 'between-added Ni(II) concentrations x 'between-participants' first-order interaction effect. The latter two models were equivalent to generalized linear model analysis systems without and with an associated interaction effect, respectively. The mathematical models for model 1, 2 and 3 designs are shown in Equations 1, 2 and 3 respectively, where $y_{ij(k)}$ are the observational values determined for each bucket evaluated, µ represents the mean value for each bucket in the absence of any source of variation, C_i the effect of added Ni(II) levels (fixed) effect, P_j the 'between-participants' (random) effect, and e_{ijk} fundamental error respectively. In Equation 3, CP_{ij} is the 'between-added Ni(II) levels' x 'between-participants'

interaction effect. Without applying covariate-adjusting designs such as in model 2, and ultimately model 3, the fundamental error term becomes markedly inflated via its combination with both P_j and CP_{ij} in model 1, and with CP_{ij} alone in model 2, and hence these approaches are expected to enhance the statistical significance of the 'between-added Ni(II) level' effect strictly on the basis of their variance contribution status.

$$y_{ij} = \mu + C_i + e_{ij} \qquad (1)$$
$$y_{ijk} = \mu + C_i + P_j + e_{ijk} \qquad (2)$$
$$y_{ijk} = \mu + C_i + P_j + CP_{ij} + e_{ijk} \qquad (3)$$

Figure S7.1 (Section S7) shows violin plots of the intensity of the top key added Ni(II)-influenced resonances, both individual observations and their median values, arising from application of the single-factor one-way ANOVA model only (model 1), in order to primarily evaluate the univariate statistical significance of the 'between-added Ni(II) concentrations' fixed effect across the series of n = 12 participant WMSS donors. Explanations for these results are also provided in Section S7.

On consideration of the Bonferroni-corrected *p* values shown in Table S3, it can be observed that the significance of the most important principal factor investigated ('between-added Ni(II) concentrations') clearly decreased following removal of the potentially confounding 'between-participants' covariate effect, with *p* values for this C_i effect markedly decreasing for as many as 42 out of a total of 56 bucket biomarkers listed for analysis by model 1; statistically significant buckets for this model increased from only 18 in model 1 to the 42 observed for model 2, and this again demonstrates the importance of removing interfering covariates for such analysis, which yields an improved precision and power of the model applied. Moreover, further removal of any interfering effects arising from the first-order CP_{ij} interaction effect (model 3) revealed that, in addition to the number of significant univariate bucket variables increasing to 9 more than that attained with model 2 (now 51), the majority of all significant buckets explored again had diminished *p* values; for example, those for the lactate-CH_3, acetate-CH_3 and formate-H resonances decreased from $<10^{-65}$ to $<10^{-86}$, $<10^{-72}$ to $<10^{-77}$, and $<10^{-13}$ to $<10^{-65}$ respectively, the latter being a highly substantial reduction. Additionally, that for ethanolamine's -CH_2OH signal decreased from $<10^{-13}$ to as little as $<10^{-65}$. For all ^{1}H NMR buckets where it was statistically significant, this interaction effect largely reflected the non-additive biomolecular coordination responses of different participants to differing levels of added Ni(II), i.e. relationships between metabolite responses and added fixed concentrations of this metal ion were not uniform amongst participants, and hence differed markedly between them, responses undoubtedly mediated by their differential salivary constituent content levels, most notably those of key Ni(II) complexants (as might be expected). Therefore, the classification of response patterns to added Ni(II) described above is rendered very complex, since it significantly differs between the WMSS sample donors recruited to the study. Only a single metabolite (methylamine) was found to be significant for model 2, but not for model 3 (Table S3).

After removing the interfering P_j and CP_{ij} effects in model 3, the most important added Ni(II)-induced ^{1}H NMR profile changes observed predominantly consisted of buckets featuring the carboxylato- and/or alcohol donor ligands, specifically lactate > acetate > formate > ethanolamine > choline/glycerol > glycolate > propane-1,2-diol/β-fucose > phenylacetate > propionate, in that order, and with at least some exceptions, these data are generally consistent with the results shown in Table 1, although it should be noted that this metabolomics approach yielded a much more extensive range of added Ni(II)-

responsive salivary metabolites than the direct visual inspection method, as expected. For model 3, only 33 out of the 85 fixed ^{1}H NMR buckets had insignificant 'between-added Ni(II) concentration' effects.

Many of the modifications observed featured added concentration-dependent Ni(II)-induced resonance broadenings and associated intensity reductions, many of which appeared to be predominantly localised to their 0.04 ppm (24 Hz) frequency bucket widths throughout all spectral titrations conducted up to a level of 0.670 mmol/L. As noted above for class 1-responding metabolites, at the lower added Ni(II) level titration points up to 280 µmol/L, concentration-based lag phases were notable, including acetate and formate resonance buckets, prior to them diminishing in intensity at the higher Ni(II) levels added.

Figure S9.1 shows corresponding plots of the F variance ratio statistic for each 0.04 ppm bucket assessed for the major C*i* source of variation for models 1, 2 and 3. The first of these plots confirms the significance of the most distinctive salivary biomolecule complexants for this metal ion for each of these models. However, corresponding plots for the P_j and CP_{ij} sources of variation in model 3 (Figures S9.1(d) and (e)) demonstrated that nearly all buckets involved had a statistically significant 'between-participants' random effect, the highest of these being those for ethanol (δ = 3.64-3.68 ppm) > propionate (δ = 1.04-1.08 ppm) > lactate (δ = 1.32-1.36 ppm) > ethanol again (δ = 1.16-1.20 ppm), > propionate again (δ = 2.16-2.20 ppm) > ethanolamine (δ = 3.14 ppm), each with F ratio values above 200. This provided much evidence for differential 'between-participant' extents of WMSS complexant reactivities towards added Ni(II). Likewise, those for the CP_{ij} interaction effect were found to be formate (δ = 8.44-8.48 ppm) > propionate (δ = 1.04-1.08 ppm) > lactate (δ = 1.32-1.36 ppm) > ethanol (δ = 3.66-3.70 ppm) > ethanolamine (δ = 3.12-3.16 ppm) > tyrosine (δ = 3.04-3.08 ppm) > propionate again (2.16-2.20 ppm). Therefore, these CP_{ij}-significant metabolites serve as rather unusual biomarkers for the heterogeneity (rather than extents) of participant responses, i.e., as featured metabolites present in their WMSS samples, towards added Ni(II) ion.

However, this form of analysis spanned the entire added Ni(II) concentration range of 0.071-0.67 mmol/L, and therefore the statistical significance of each bucket tested applies to all metabolite responses, and is concentration-weighted in this context on the degree of statistical significance of added Ni(II)-salivary metabolite responses at all added Ni(II) increments, and not those differences found at low added Ni(II) levels only, as indeed it was for the analyses described in Section 3.6.3.

(a)

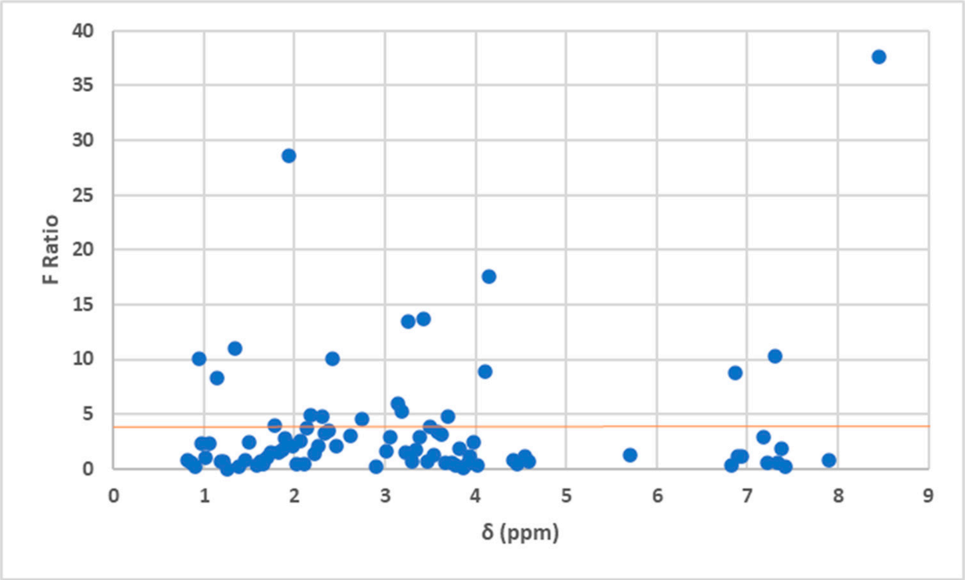
F Ratio
δ (ppm)
0
5
10
15
20
25
30
35
40
0 1 2 3 4 5 6 7 8 9

(b)

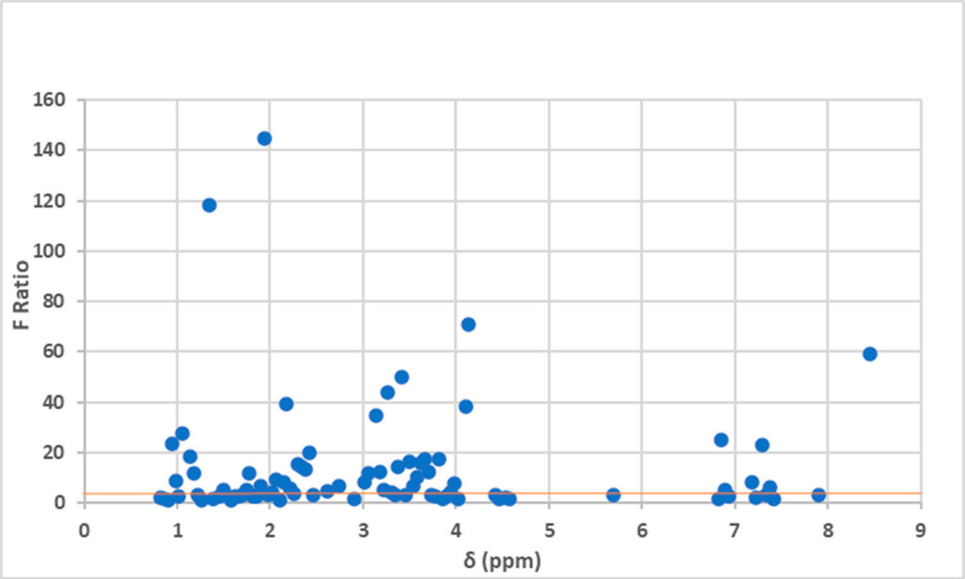
F Ratio
δ (ppm)
0
20
40
60
80
100
120
140
160
0 1 2 3 4 5 6 7 8 9

(c)

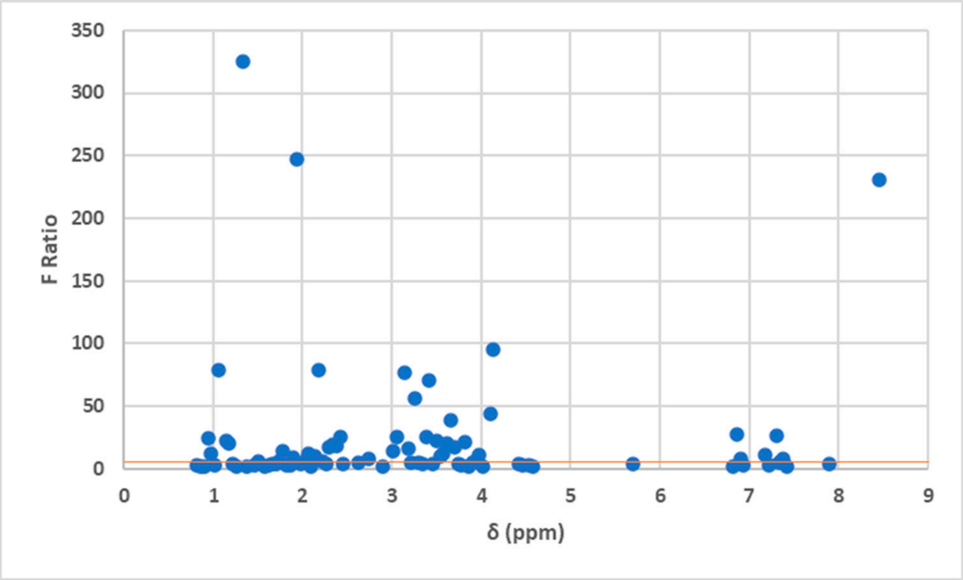
F Ratio
δ (ppm)
350
300
250
200
150
100
50
0
0
1
2
3
4
5
6
7
8
9

(d)

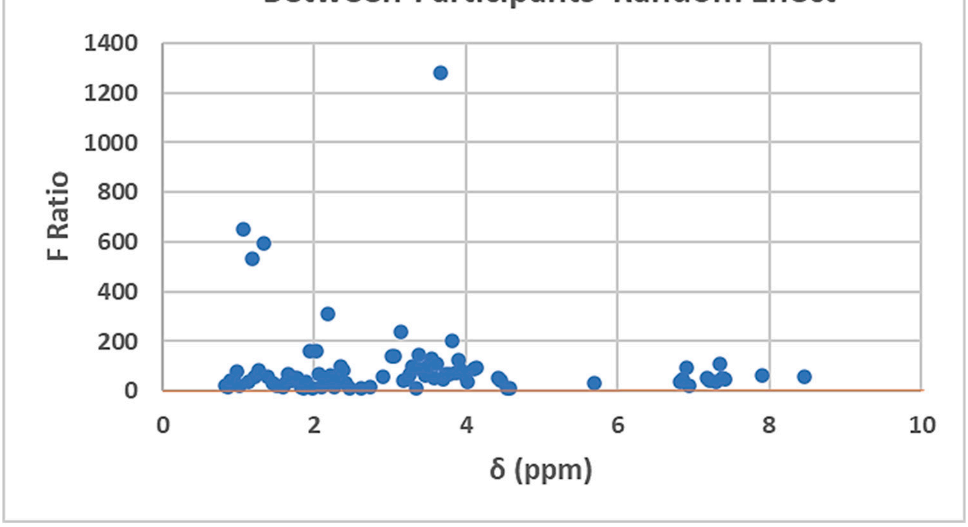
'Between-Participants' Random Effect
F Ratio
δ (ppm)
1400
1200
1000
800
600
400
200
0
0
2
4
6
8
10

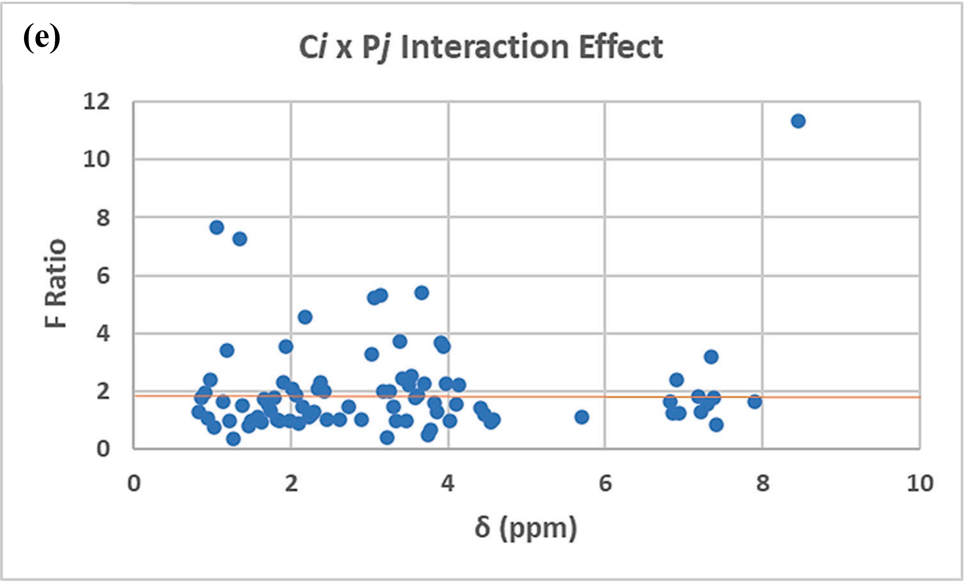

Figure S9.1 (a), (b) and (c), plots of F ratio statistic values determined in ANOVA models 1, 2 and 3, respectively, applied to all fixed ^{1}H NMR buckets for evaluating differences 'between-added Ni(II) concentrations' (fixed effect). The horizontal orange lines indicate the critical Bonferroni-corrected F ratio parameter required for statistical significance with a total of 85 fixed bucket variables, specifically 4.11, 4.13 and 4.20, for testing of the main 'between-added Ni(II) concentrations' factor for models 1, 2 and 3 respectively. (d) and (e), plots of F ratio statistic values determined in the ANOVA model 3 system applied to all fixed ^{1}H NMR buckets for evaluating differences 'between-participants' (random effect) and the first-order 'between-Ni(II) concentrations' x 'between-participants' interaction effect respectively. The horizontal orange lines again indicate the critical Bonferroni-corrected F ratio statistic required for statistical significance with a total of 85 fixed bucket variables. For all plots, the chemical shift value refers to the 0.02 ppm mid-point of each 0.04 ppm bucket.

Table S3. Statistical significance of ^{1}H NMR-assigned metabolite variables from the application of increasingly advanced univariate ANOVA models to the Ni(II) speciation dataset comprising n = 12 participant sample donors, 7 added Ni(II) concentrations (including the zero control specimen) per sample collected, and n = 3 replicate spectra acquired. The second, third and fourth columns show statistical significance (*p* value) results arising from ANOVA-based analyses conducted according to either models 1, 2 and 3 respectively, which considered the C_i (fixed) effect only, the C_i and P_j (random) effects together, and all possible sources of variation (i.e., C_i, P_j and CP_{ij} effects) respectively (equations 1-3). Probability (*p*) values given are Bonferroni-corrected. Also provided are spectral assignments for the stignificant 0.04 ppm-fixed ^{1}H NMR frequency buckets listed (the limited number of resonances which were partially 'split' between two 0.04 ppm bucket regions are indicated as those having two frequency unit assignments). The classification of each potential Ni(II) complexant or interactor (i.e., classes 1-4, as specified in Section S8 above) is also listed.

^{1}H NMR Bucket (ppm)	Bonferroni-Corrected One-Way ANOVA Design *p* Value (Model 1)	Bonferroni-Corrected Two-Way ANOVA Design *p* Value (Model 2)	Bonferroni-Corrected Two-Way ANOVA with Interaction Design *p* Value (Model 3)	Assignment (Ni(II) Rank Order (Model 3))	Ni(II) Complexant/ Responder Class (1-4)
[1.32 .. 1.36]	2.24×10^{-9}	6.91×10^{-66}	1.17×10^{-87}	Lactate-C<u>H</u>$_3$ (1)	1
[1.92 .. 1.96]	7.73×10^{-24}	8.84×10^{-73}	1.75×10^{-78}	Acetate-C<u>H</u>$_3$ (2)	1

[8.44 .. 8.48]	2.25×10^{-28}	4.55×10^{-42}	2.88×10^{-76}	Formate-$\underline{H}$ (3)	1
[3.80 .. 3.84]	ns	1.81×10^{-14}	2.43×10^{-66}	Ethanolamine-C$\underline{H}_2$OH (4)	2
[3.52 .. 3.56]	ns	2.38×10^{-04}	9.52×10^{-53}	Choline-CH$_2$OH/Glycerol-C$\underline{H}_2$OH/Glycine-C$\underline{H}_2$ (5)	2
[3.88 .. 3.92]	ns	ns	2.18×10^{-52}	Glycolate-C$\underline{H}_2$ (6)	n/a
[4.12 .. 4.16]	4.22×10^{-15}	7.96×10^{-48}	3.51×10^{-49}	Lactate-C$\underline{H}$ (7)	1
[3.60 .. 3.64]	ns	5.10×10^{-13}	8.92×10^{-49}	Propane-1,3-diol-C$\underline{H}_2$OH/β-Fucose-C2$\underline{H}$ (8)	2
[3.48 .. 3.52]	ns	2.73×10^{-13}	2.34×10^{-46}	Phenylacetate-α-C$\underline{H}_2$ (9)	2
[2.16 .. 2.20]	6.09×10^{-3}	8.42×10^{-31}	5.53×10^{-44}	Propionate-C$\underline{H}_2$ (10)	n/a
[1.04 .. 1.08]	ns	1.67×10^{-22}	6.11×10^{-44}	Propionate-C$\underline{H}_3$ (11)	n/a
[3.12 .. 3.16]	5.42×10^{-4}	1.15×10^{-27}	2.53×10^{-43}	Dimethylsulphone-C$\underline{H}_3$s/1/2 Histidine-β-C$\underline{H}_2$/Phenylalanine-β-C$\underline{H}_2$/Ethanolamine-C$\underline{H}_2$$NH_3^+$ (12)	2
[3.40 .. 3.44]	1.28×10^{-11}	3.15×10^{-37}	6.50×10^{-41}	Taurine-C$\underline{H}_2$$SO_3^-$ (13)	2
[3.24 .. 3.28]	2.12×10^{-11}	2.01×10^{-33}	3.98×10^{-35}	Taurine-C$\underline{H}_2$$NH_3^+$ (14)	4
[3.96 .. 4.00]	ns	4.88×10^{-05}	2.54×10^{-33}	Phosphocreatine-C$\underline{H}_2$ (15)	4
[3.84 .. 3.88]	ns	ns	2.82×10^{-33}	α-Fucose-C3$\underline{H}$ (16)	n/a
[3.76 .. 3.80]	ns	ns	7.36×10^{-32}	Guanidoacetate-C$\underline{H}_2$/Alanine-α-C$\underline{H}$/Glycerol-C$\underline{H}$OH (17)	n/a
[4.00 .. 4.04]	ns	ns	1.86×10^{-31}	Creatinine-C$\underline{H}_2$/Tryptophan-α-C$\underline{H}$ (18)	n/a
[3.68 .. 3.72]	ns	1.18×10^{-9}	2.34×10^{-30}	1-Methylhistidine-N1(C$\underline{H}_3$)/3-Methylhistidine-N3(C$\underline{H}_3$)/Lysine-α-C$\underline{H}$ (19)	2
[3.56 .. 3.60]	ns	1.61×10^{-07}	5.02×10^{-30}	Glycine-C$\underline{H}_2$ (20)	n/a
[3.72 .. 3.76]	ns	ns	6.69×10^{-30}	Leucine-α-C$\underline{H}$/Gluconate-C4$\underline{H}$/C5$\underline{H}$ (21)	n/a
[3.44 .. 3.48]	ns	ns	8.40×10^{-30}	β-Fucose-C2$\underline{H}$/Taurine-C$\underline{H}_2$$SO_3^-$ (22)	3
[3.64 .. 3.68]	ns	2.24×10^{-14}	2.09×10^{-29}	Ethanol-C$\underline{H}_2$ OH (23)	n/a
[4.08 .. 4.12]	6.22×10^{-7}	3.88×10^{-30}	8.58×10^{-29}	Proline-α-C$\underline{H}$/Lactate-C$\underline{H}$ (24)	1
[6.84 .. 6.88]	7.68×10^{-7}	1.45×10^{-20}	7.28×10^{-20}	4-hydroxyphenylacetate aromatic ring-C3$\underline{H}$/C5$\underline{H}$ protons (25)	2
[7.28 .. 7.32]	2.59×10^{-8}	8.84×10^{-19}	1.68×10^{-19}	Phenylalanine aromatic ring-C2$\underline{H}$/C6$\underline{H}$ protons (26)	2
[2.40 .. 2.44]	3.55×10^{-8}	1.40×10^{-16}	4.94×10^{-19}	Succinate-C$\underline{H}_2$s (27)	1
[3.04 .. 3.08]	ns	5.99×10^{-9}	1.35×10^{-18}	Tyrosine-β-C$\underline{H}_2$ (28)	3
[3.36 .. 3.40]	ns	2.32×10^{-11}	1.81×10^{-18}	Methanol-C$\underline{H}_3$ (29)	2
[0.92 .. 0.96]	ns	1.57×10^{-19}	5.89×10^{-18}	Leucine-C$\underline{H}_3$/Isoleucine-C$\underline{H}_3$ (30)	1
[1.12 .. 1.16]	2.34×10^{-6}	2.78×10^{-15}	1.74×10^{-16}	*Iso*-Butyrate-C$\underline{H}_3$s/3-AIB-C$\underline{H}_3$ (31)	2
[2.32 .. 2.36]	ns	2.08×10^{-13}	5.62×10^{-16}	Glutamate-γ-C$\underline{H}_2$/Malate-1/2C$\underline{H}_2$ (tentative assignment) (32)	n/a
[1.16 .. 1.20]	ns	3.22×10^{-9}	7.19×10^{-15}	Ethanol-C$\underline{H}_3$ (33)	n/a
[2.36 .. 2.40]	ns	1.70×10^{-10}	1.33×10^{-13}	Pyruvate-C$\underline{H}_3$ (34)	n/a
[2.28 .. 2.32]	ns	1.44×10^{-12}	1.43×10^{-12}	Acetoacetate-COC$\underline{H}_3$ (35)	n/a

[3.16 .. 3.20]	2.83 x 10^{-3}	2.14 x 10^{-9}	1.49 x 10^{-11}	Ethanolamine-C$\underline{H}_2$NH$_2$/9-Methylurate-C$\underline{H}_3$/Choline-N(C$\underline{H}_3$)$_3$ (36)	4
[1.76 .. 1.80]	ns	3.99 x 10^{-9}	1.44 x 10^{-10}	Leu-β-C$\underline{H}$ (37)	3
[0.96 .. 1.00]	ns	3.34 x 10^{-6}	8.92 x 10^{-9}	Leu-C$\underline{H}_3$/Val-C$\underline{H}_3$ (38)	n/a
[2.04 .. 2.08]	ns	9.77 x 10^{-7}	2.71 x 10^{-8}	GlycA-NHCOC$\underline{H}_3$/Free N-Acetylsugar-NHCOC$\underline{H}_3$ (39)	4
[7.16 .. 7.20]	ns	ns	4.04 x 10^{-7}	4-Hydroxyphenylacetate aromatic ring-C3$\underline{H}$/C5$\underline{H}$ (40)	n/a
[7.32 .. 7.36]	ns	ns	1.09 x 10^{-7}	Phenylalanine aromatic ring-C2$\underline{H}$/C6$\underline{H}$ (41)	n/a
[2.12 .. 2.16]	ns	1.01 x 10^{-5}	2.62 x 10^{-6}	Methionine-SC$\underline{H}_3$ (42)	n/a
[1.88 .. 1.92]	ns	5.90 x 10^{-4}	7.95 x 10^{-6}	Proline-γ-C$\underline{H}_2$ (43)	n/a
[2.72 .. 2.76]	1.47 x 10^{-2}	4.43 x 10^{-4}	1.19 x 10^{-4}	Dimethylamine-N(C$\underline{H}_3$)$_2$ (44)	2
[7.36 .. 7.40]	ns	1.57 x 10^{-3}	1.70 x 10^{-4}	Phenylalanine aromatic ring-C4$\underline{H}$ (45)	n/a
[6.88 .. 6.92]	ns	1.04 x 10^{-2}	2.20 x 10^{-4}	Tyrosine aromatic ring-C2$\underline{H}$/C6$\underline{H}$ (46)	4
[3.92 .. 3.96]	ns	ns	48 4.41 x 10^{-4}	Creatine-C$\underline{H}_2$ (47)	n/a
[2.20 .. 2.24]	ns	3.97 x 10^{-3}	4.15 x 10^{-3}	Acetone-C$\underline{H}_3$s (48)	n/a
[1.72 .. 1.76]	ns	2.26 x 10^{-2}	1.17 x 10^{-2}	Lysine-δ-C$\underline{H}_2$ (49)	3
[2.00 .. 2.04]	ns	ns	1.26 x 10^{-2}	GlycA-NHCOC$\underline{H}_3$ (50)	2
[1.48 .. 1.52]	ns	ns	2.21 x 10^{-2}	Alanine-C$\underline{H}_3$ (51)	3
[3.20 .. 3.24]	ns	8.12 x 10^{-3}	ns	Choline-N(C$\underline{H}_3$)$_3^+$/Taurine-C$\underline{H}_2$NH$_2$ (52)	4
[2.60 .. 2.64]	ns	3.87 x 10^{-2}	ns	Methylamine-N(C$\underline{H}_3$) (53)	n/a

Section S10: Application of a MV ASCA model to analysis of the added Ni(II) ion- and participant-dependent metabolomics dataset

An ASCA model was applied to the metabolomics dataset, and for this purpose with the major factors 'between-added Ni(II) levels' and 'between-participants' were considered, along with their first-order interaction variance effect. Scree plots for the main factors proved that for the first (added Ni(II) level)-dependent main effects, no more than two or three PCs were necessary to satisfy the model, with the first of these accounting for 79.4% of the total variance. For the second 'between-participants' effect, however, overall four PCs were required, the first of which accounted for 30.5% of the total model variance. These results were largely consistent with those shown in the interactive PCA diagram shown in Figure 4. There was a clear, almost linear response of PC1 score value to increasing added Ni(II) concentration (data not shown). Figure S10.1 shows the results of permutation testings for the statistical significance of each of these effects. Results acquired demonstrated that a model with only the two main factors, but not also including the first-order interaction effect (CP_{ij}), was sufficient to account for the MV ASCA dataset (p = <0.005, <0.005 and 0.76 respectively). Hence the 'between-participants' effect was responsible for a similar contribution towards the total model variation to that of the added Ni(II) level. However, it should also be noted that conducting this ASCA model with n = 12 participants rather than n = 7 markedly improved its significance level and hence its 'between-added Ni(II) concentrations' discriminatory potential; indeed, the p value attained with the lower participant number experimental protocol was only 0.035. The most important salivary biomolecules found for the added Ni(II) ion concentration factor were formate (cut-off leverage (Lev) 0.303/squared prediction errors (SPE) 1.275)

and both lactate-$C\underline{H}_3$ and -$C\underline{H}$ signals (Lev 0.075 and 0.055 respectively/SPE 0.270 and 0.175 respectively), whereas the only one found for the 'between-participants' covariate was urea (exchangeable -$CON\underline{H}_2$ protons, broad δ = 5.68-5.72 ppm (*s*) resonance) with associated Lev 0.048 /SPE 29.227 parameters. Those for the CP_{ij} interaction effect were unassigned signals located at δ = 0.80-0.84 ppm (Lev 0.152/SPE 6.261), N-acetylneuraminate-C3$\underline{H}$ at δ = 1.84-1.88 ppm (Lev 0.105/SPE 6.548), *n*-butyrate-$C\underline{H}_3$, δ = 0.84-0.88 ppm (Lev 0.077/SPE 0.865), methylamine-N($C\underline{H}_3$) at δ = 2.60-2.64 ppm (Lev 0.073/SPE 6.265), 5-aminovalerate-α-$C\underline{H}_2$/acetone-$COC\underline{H}_3$ at δ = 2.44-2.48 ppm (Lev 0.070/SPE 4.267), and methanol-$C\underline{H}_3$ at δ = 3.36-3.40 ppm (Lev 0.064/SPE 1.249). Variables with relatively high leverage and low SPE values are considered to be well modelled.

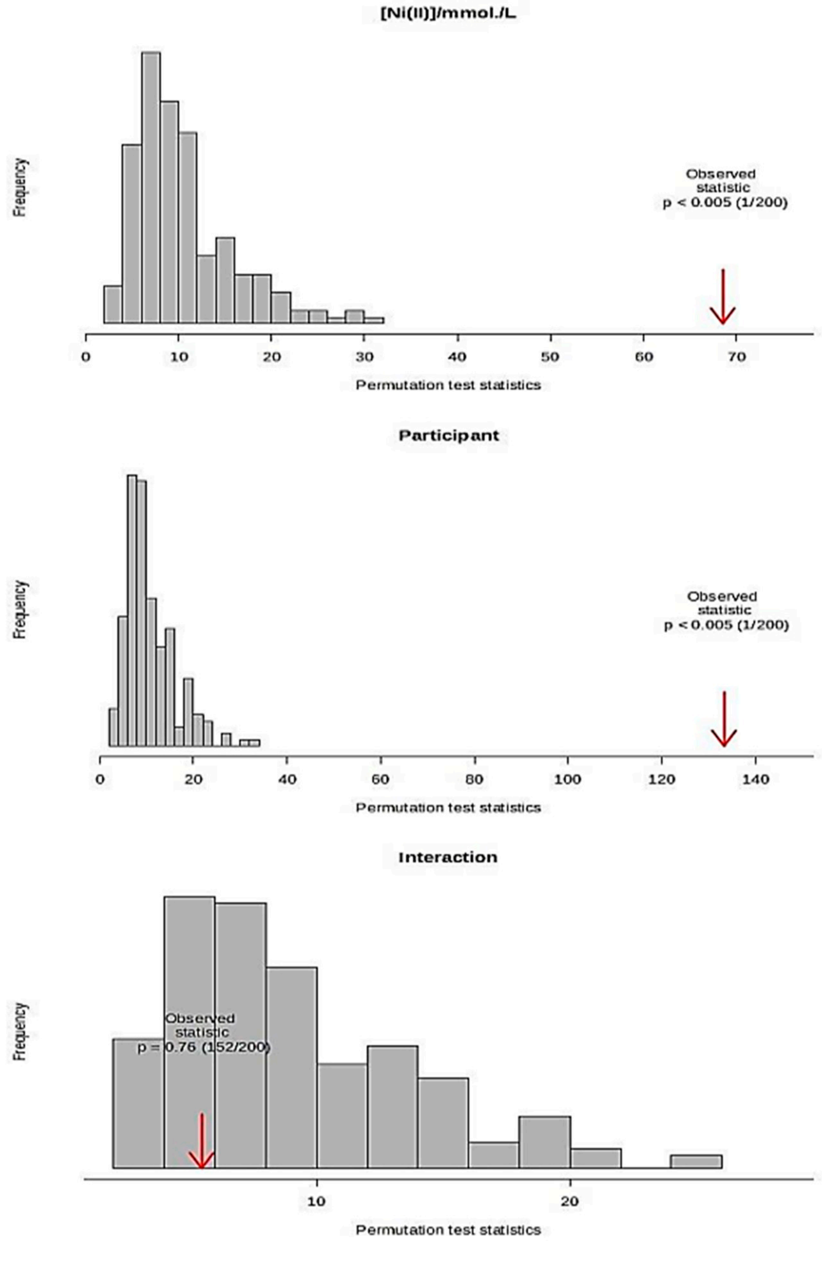

Figure S10.1. Histogram plots showing the results of permutation testing of the model variance contributions for the added Ni(II) concentration (top), 'between-participants' (middle), and 'between-added Ni(II) concentrations' x 'between-participants' first-order interaction effect (bottom) for the

ASCA model applied to our ^{1}H NMR spectral titration dataset. A total of 200 permutations were performed to verify model validity.

Section S11: Ni(II) ion speciation experiments conducted with the pooled WMSS QC sample

Application of the one-way ANOVA approach produced a rank order on important Ni(II) ion-interacting metabolites. This order, which corresponds to Bonferroni-corrected *p* values, of the top 10 significant WMSS metabolite proton buckets, was formate-H (p = 1.91 x 10^{-18}) > ethanol-C$\underline{H}_3$ (2.60 x 10^{-6}) > succinate-C$\underline{H}_2$ (5.58 x 10^{-6}) > acetate-C$\underline{H}_3$ (4.34 x 10^{-5}) > ethanol-C$\underline{H}_2$ (1.53 x 10^{-4}) > propionate-C$\underline{H}_3$ (2.71 x 10^{-4}) > ethanolamine-C$\underline{H}_2$$NH_2$ (3.91 x 10^{-4}) > lactate-C$\underline{H}_3$ (1.34 x 10^{-4}) > leucine-C$\underline{H}_3$/isoleucine-C$\underline{H}_3$s (5.72 x 10^{-3}) > 3-AIB-C$\underline{H}_3$/*iso*-propanol-C$\underline{H}_3$ (5.72 x 10^{-3}). Hence, all major Ni(II) complexants identified were again largely restricted to those containing carboxylato- and/or alcohol group oxygen-donor ligands, with the exception of ethanolamine, which may also involve complexation with its amine function N-donor atom. Additional potential ligands lying beyond the above top 10 included resonance buckets arising from glutamate/malate, pyruvate and taurine (both ^{1}H NMR signals for the latter). Therefore, with a few minor exceptions, the top 10 Ni(II) complexants found for this pooled WMSS sample were fully consistent with those obtained when such experiments were conducted on n = 12 individual WMSS specimens.

However, since there was only one uniform pooled sample solution available for these experiments, comparisons between the untreated control and lowest added Ni(II) level (0.071 mmol/L) were restricted to *post-hoc* ANOVA analysis of these two classifications. This analysis revealed that in addition to formate (Bonferroni-corrected *p* value 2.84 x 10^{-5}), there were also significant changes in the resonance intensities of buckets ascribable to glycolate, α-fucose, acetoacetate and phenylalanine, with marginal reductions also found for acetate, but these results should be interpreted with caution in view of the limited sample size available for this particular assessment.

Results arising from a PLS-DA model applied to the pooled WMSS dataset are shown in Table S4. This analysis found that models with all 7 different added Ni(II) levels were adequately fitted, with cross-validated Q^2 values of >0.92 for all models, and 4-8 components considered (R^2Y = >0.98). Model VIP values for the top 20 Ni(II)-responsive biomarkers are also provided (values >1.00 are considered to be strongly significant). Again, with the exception of a few WMSS metabolites such as the amino acids histidine, tyrosine and phenylalanine, the majority of these markers featured O-donor atom carboxylato-ligands, with formate > lactate > succinate > acetate indicated as being the most important. Cross-validation of this model with the Q^2 statistic showed that this value was ≥0.92 for models with four or more components (corresponding R^2Y indices were ≥0.98), and this confirmed the excellent predictive capacity of it for our pooled WMSS dataset.

Table S4. Application of the PLS-DA technique to the analysis of the pooled WMSS sample treated with increasing added Ni(II) concentrations. Variable importance parameter (VIP) values for a model considering differences between 7 levels of added Ni(II) levels (including the zero untreated control sample) are listed; 3 separate metal ion titrations for the pooled sample were performed. Biomolecular assignments for ^{1}H NMR bucket resonances are provided. The limited number of resonances which were partially 'split' between two 0.04 ppm bucket regions are indicated as those having two frequency unit assignments. Abbreviations: 3-AIB, 3-aminoisobutyrate-CH_3.

^{1}H NMR Bucket	PLS-DA VIP Value (Component 1)	^{1}H NMR Assignment
[8.44 .. 8.48]	3.77	Formate-$\underline{H}$
[1.32 .. 1.36]	2.39	Lactate-$C\underline{H}_3$
[2.40 .. 2.44]	2.35	Succinate-$C\underline{H}_2$
[1.92 .. 1.96]	1.84	Acetate-$C\underline{H}_3$
[7.20 .. 7.24]	1.79	Tyrosine-aromatic Ring-C3$\underline{H}$/C5$\underline{H}$
[7.40 .. 7.44]	1.60	Phenylalanine-aromatic ring-C3$\underline{H}$/C5$\underline{H}$
[1.16 .. 1.20]	1.50	Ethanol-$C\underline{H}_3$
[6.88 .. 6.92]	1.45	Tyrosine-aromatic ring-C2$\underline{H}$/C6$\underline{H}$
[1.12 .. 1.16]	1.40	*iso*-Butyrate-$C\underline{H}_3$s/3-AIB-CH_3
[3.12 .. 3.16]	1.40	Dimethylsulphone-$C\underline{H}_3$s/1/2 Histidine-/Phenylalanine-β-$C\underline{H}_2$/Ethanolamine-$C\underline{H}_2NH_2$
[3.40 .. 3.44]	1.37	Taurine-$C\underline{H}_2SO_3^-$
[2.44 .. 2.48]	1.33	Glutamine-γ-$C\underline{H}_2$
[4.52 .. 4.56]	1.32	β-Glucose-C1$\underline{H}$ (trace/tentative assignment)
[3.36 .. 3.40]	1.30	Methanol-$C\underline{H}_3$
[3.48 .. 3.52]	1.20	Phenylacetate-$C\underline{H}_2$
[2.28 .. 2.32]	1.18	Acetoacetate-$COC\underline{H}_3$
[2.88 .. 2.92]	1.17	TMA-$N(C\underline{H}_3)_3$
[2.36 .. 2.40]	1.16	Pyruvate-$C\underline{H}_3$
[4.56 .. 4.60]	1.14	Carbohydrate anomeric-C1$\underline{H}$ proton(s) trace/tentative assignment)
[4.12 .. 4.16]	1.13	Lactate-$C\underline{H}$

Section S12: Stepwise stability constants for Ni(II)-biomolecule complexes and their literature sources

Table S5. Stepwise stability constants for the 1:1, 1:2 and, if applicable, 1:3 complexes of Ni(II) with a variety of salivary biomolecular ligands/chelators (all values were extracted from the scientific literature and referenced accordingly). Also shown is the estimated mean salivary ligand/chelator concentration, along with the ANOVA- and visually-rated ^{1}H NMR-based complexation order (ANOVA-based orders were determined from decreasing *p* values for the significance of the Bonferroni-corrected two-way ANOVA model conducted, which included consideration of the first-order interaction effect (model 3 in Table S3)). Abbreviations: na, not applicable; nd, not determined. *Indicates that this metabolite was found to serve as one of the most important complexants for added Ni(II) in the current study, but only at this metal ion's lowest added concentrations (0-0.14 mmol/L).

Ligand/Chelator	β_1 Stability Constant	β_2 Stability Constant	β_3 Stability Constant	Mean WMSS Concentration (mmol/L)	Model 3 ANOVA-Based Complexation Order (^{1}H NMR Visual Computational Order)
**Citrate**	$10^{5.49}$ [S3]	$10^{2.1}$ [S3]	Unavailable	<0.05 mmol/L [28]	nd (nd)
**Lactate**	39 [S1]	853 [S1]	Unavailable	13.19 mmol/L [S2]	1 (1)
**Glycolate**	51 [S5,S6]	508 [S5,S6]	Unavailable	21.6±13.0 μmol/L [37]	6 (na)
**Succinate**	$10^{4.47}$ [37]	$10^{9.58}$ [37]	Unavailable	1.36 mmol/L [S2]	>20 (1)
**β-Hydroxypropionate**	7.5 [S7]	19 [S7]	Unavailable	Unknown	na (na)
**Propionate**	7.5 [S5,S6]	16 [S5,S6]	Unavailable	21.46 mmol/L [S8]	10/11 (3)
***n*-Butyrate**	6.5 [S11,S12]	6.5 [S11,S12	Unavailable	0.41 mmol/L [S2]	nd (>6)
**Acetate**	6.5 [S5,S6]	9.9 [S5,S6]	Unavailable	108.18 mmol/L [S2]	2 (3)
**Formate**	3.8 [S5,S6]	7.2 [S5,S6]	Unavailable	11.94 mmol/L [S2]	3 (2)
**3-D-Hydroxybutyrate**	Unavailable	Unavailable	Unavailable	16.44±19.52 μmol/L [S14]	>20 (4)
**Pyruvate**	$10^{0.76}$ [S7]	$10^{0.83}$ [S7]	$10^{1.67}$ [S7]	1.67 mmol/L [17]	>20 (2)
**5-Aminovalerate**	Unavailable	Unavailable	Unavailable	470.38±342.79 μmol/L [37]	>20 (4)
**3-Aminoisobutyrate**	Unavailable	Unavailable	Unavailable	Unavailable	>20 (4)
**Adenine**	$10^{6.66}$ [S8]	$10^{12.22}$ [S8]	Unavailable	1.19±1.25 μmol/L [37]	>20 (4)
**Aspartate**	$10^{7.14}$ [S8]	$10^{12.43}$ [S8]	Unavailable	33.30± 19.39 μmol/L [37]	nd (nd)
**Glutamate**	$10^{5.34}$ [S8]	$10^{10.36}$ [S8]	$10^{13.43}$ [S8]	13.6±2.4 μmol/L [S17]	>20 (>6)
**Asparagine**	$10^{8.02}$ [S8]	$10^{18.29}$ [S8]	$10^{25.55}$ [S8]	1.76±2.11 μmol/L [S15]	nd (nd)
**Leucine**	$10^{5.53}$ [S8]	$10^{11.78}$ [S8]	Unavailable	53.50± 32.95 μmol/L [37]	>20 (>6)
**Glycine**	$10^{5.56}$ [S9]<br>$10^{9.62}$ [68]	$10^{9.55}$ [S9]<br>$10^{13.32}$ [68]	$10^{11.68}$ [S11]	177.80±143.20 μmol/L [37]	20 (3)
**Alanine**	$10^{9.69}$ [S10]	$10^{11.88}$ [S10]	Unavailable	145.27±108.41 μmol/L [37]	>20 (4)
**Histidine***	$10^{8.68}$ [S4]	$10^{15.51}$ [S4]	$10^{17.81}$ [S11]	18.67±10.20 μmol/L [37]	1*(1)
**Tryptophan**	$10^{5.45}$ [S8]	$10^{9.64}$ [S8]	$10^{13,62}$ [S8]	4.80±3.41 μmol/L [37]	18 (nd)
**Phenylalanine**	$10^{6.52}$ [S8]	$10^{10.10}$ [S8]	$10^{16.58}$ [S8]	43.70±23.30 μmol/L [S16]	>20 (>6)
**Cysteine**	$10^{9.77}$ [S10]	$10^{17.67}$ [S10]	Unavailable	1.2 μmol/L [S17]	nd (nd)
**Ornithine**	$10^{10.58}$ [S10]	$10^{19.43}$ [S10]	$10^{21.39}$ [S14]	65.34±15.64 μmol/L [37]	nd (nd)
**Lysine**	$10^{10.44}$ [S10]	$10^{19.66}$ [S10]	Unavailable	37.76 +/- 18.10 uM [37]	>20 (nd)
**Methionine**	$10^{9.12}$ [S10]	$10^{11.10}$ [S10]	Unavailable	8.81±5.94 μmol/L [37]	nd (nd)
**Threonine**	$10^{9.06}$ [S10]	$10^{11.03}$ [S10]	Unavailable	26.75±28.12 μmol/L [37]	nd (nd)
**Proline**	$10^{10.49}$ [S10]	$10^{12.03}$ [S10]	Unavailable	158.48±122.10 μmol/L [37]	nd (nd)
**Valine**	$10^{9.57}$ [S10]	$10^{11.70}$ [S10]	Unavailable	47.97±33.60 μmol/L [36]	>20 (>6)
**Choline**	Unavailable, but ionic liquid formed on	Unavailable	Unavailable	32.73±7.60 μmol/L [37]	5 (5/6)

	interaction [S11]				
**GABA**	Complex formed [S12]	Complex formed [S12]	Unavailable	⩽100 μmol/L [11]	>20 (5/6)
**MA**	Complex formed [S13]	Unavailable	Unavailable	101 μmol/L [11]	>20 (5/6)
**DMA**	Unavailable	Unavailable	Unavailable	65 μmol/L [11]	15 (5/6)
**TMA**	Unavailable	Unavailable	Unavailable	98 μmol/L [11]	>20 (5/6)
**SCN^-**	$10^{1.8}$ [S14] (in methanol solution)	$10^{3.0}$ [S14] (in methanol solution)	Unavailable	661 (range 293-1,029) μmol/L [S18]	na (na)
**Ethanol**	$10^{1.7}$ [S19]	101.8 [S19]	Unavailable	68.67±43.27 μmol/L [S19]	>20 (1)

Section S13: Previously reported NMR-based studies of metal ion speciation in human biofluids

Our group has previously employed high-resolution NMR analysis to explore the precise molecular nature of iron(II)/iron(III) ions in blood serum and plasma collected from patients with iron overload (idiopathic haemachromatosis) and healthy controls [S20]; titanium(IV) [S21], aluminium(III) [S22] and vanadium(III)/vanadium(IV)/vanadium(V) [S23], cobalt(II) [S24] and chromium(III) [S25] in osteoarthritic synovial fluid; and Ca^{2+} and Mg^{2+} ions in a range of biofluids, which was explored in Ref. [30]. However, with the exception of metal alloy oral implant-relevant cobalt(II) ions [19], and Ca^{2+} and Mg^{2+} ions documented in Ref. [18], to date this technique has not been utilised to speciate any other metal ions in human saliva.

In the above ^1H NMR study focussed on the speciation of added Co(II) in human saliva [19], this added metal ion was found to be largely complexed to or chelated by lactate > formate > histidinate > succinate, and these results are not dissimilar to those obtained in the current study based on Ni(II). However, further ^1H NMR-based investigations provided evidence that both Ca^{2+} and Mg^{2+} ions were predominantly chelated by citrate in this biofluid, but only when its levels were sufficiently high, such as directly following tooth-brushing episodes, or the dietary consumption of citrate-rich foods and/or soft drinks [18].

Section S14: Risk factors for dietary Ni(II) intakes in humans

Reference concentration values for nickel in blood serum are only <0.3 μg/L, which is equivalent to <5 nmol/L, a value being much lower than that of human saliva [21]. However, estimates for daily nickel intake vary markedly in view of differential geographical availabilities for it, this value being as high as 4 mg/day in a number of Scandinavian diets [S26]. In the USA, a 'normal' diet yields an average daily intake of 0.50 mg, and 10-40% of nickel consumed is absorbed [S26-S28], although vitamins and dietary supplements, together with the drinking of 'regular' water, can also serve as nickel sources [S27].

In 2020, the European Food Safety Authority (EFSA) Panel on Contaminants in the Food Chain (CONTAM) evaluated more than 47,000 analytical reports on nickel occurrence in order to determine both acute and chronic dietary exposure of humans to this metal ion [S29]. For this purpose, an enhanced post-implantation loss in rats was employed as the critical risk characterization effect of chronic oral exposure, and a benchmark dose (lower confidence limit) ($BMDL_{10}$) value of 1.3 mg Ni/kg body weight (bw) per day was chosen as the threshold point for computing a tolerable daily intake of 13 μg/kg bw. However, a 4.3 μg Ni/kg bw value for the lowest-observed-adverse-effect-level served as

a reference point. The 95th percentile lower-bound/upper-bound (LB/UB) chronic dietary exposure level was found to be lower than the total daily intake (TDI) in adolescents and all other adult age groups; however, they exceeded that value in toddlers and some infants. Therefore, this finding was considered to provide a health concern in these young age sectors. Additionally, the 95th percentile margin of exposure (MOE) value for the mean UB acute dietary exposure was found to provide a potential low health concern for nickel-sensitized subjects.

Supplementary Information References

S1. Weil, M. Redetermination of nickel(II) formate dihydrate. *IUCrData* **2018**, *3*, x180428 https://doi.org/10.1107/S2414314618004285

S2. Zhou, Z.-H.; Ye, J.-J.; Zhong, H.; Ng, S.W. Diaquadi(l-lactato)nickel(II). *Acta Cryst.* **2002**, E58, m326-m327.

S3. Hedwig, G.R.; Liddle, J.R.; Reeves, R.D. Complex formation of nickel (II) ions with citric acid in aqueous solution: a potentiometric and spectroscopic study. *Austral. J. Chem.* **1980**, *33*(8), 1685-1693.

S4. Kaczmarek, P.; Jeżowska-Bojczuk, M.; Bal, W.; Kasprzak, K.S. Determination of the stability constants and oxidation susceptibility of nickel(II) complexes with 2′-deoxyguanosine 5′-triphosphate and l-histidine. *J. Inorg. Biochem.* **2005**, *99*(3), 737-746. ISSN 0162-0134. https://doi.org/10.1016/j.jinorgbio.2004.12.006.

S5. Filipovic, I.; Matusinovic, T.; Mayer, B., Piljac, I.; Bach-Dragutinovic, B.; Bujak, A. On the stability of formato, acetato, propionato, butyrato, glycolato and chloroacetato complexes of cobalt, nickel, copper, zinc, cadmium and lead. *Croat. Chemica. Acta* **1970**, *42*, 541-545.

S6. Grabarić, B.; Filipović, I. Spectrophotometric determination of stability constants of formato, acetato, propionato, butyrato, glycolato and chloroacetato complexes of cobalt, nickel and copper. *Croatica Chemica Acta.* **1970**, *42*(3), 479-492.

S7. Medancic, R.; Krnhak, I.; Mayer, B.; Filipovic, I. The Investigation of the stability of 2-oxopropanoate complexes of cobalt(II), nickel(Il), copper(II), zinc(II), cadmium(II), and lead(II) in aqueous solutions. *Croatica Chemica Acta.* **1980**, *53*(3), 419-424.

S8. Türkel, N. Stability constants of mixed ligand complexes of nickel(II) with adenine and some amino acids. *Bioinorg. Chem. Applications* vol. **2015**, Article ID 374782, 9 pages. https://doi.org/10.1155/2015/374782

S9. Eltaboni, F.; Elmagbari, F.; Hammouda, A.; El-Ferjani, R.; Ben Amer, Y.; Bennour, H.; Alsaqir, A. Determination of thermodynamic stability of Ni (II) glycine complexes in aqueous solution: Potentiometric and spectroscopic studies. *Internat. J. New Chem.* **2023**, *10*(4), 223-235.

S10. Ammar, R.A.; Al-Mutiri, E.M.; Abdalla, M.A. The determination of the stability constants of mixed ligand complexes of adenine and amino acids with Ni(II) by potentiometric titration method. Fluid Phase Equilibria **2011**, 301(1), 51-55. ISSN 0378-3812, https://doi.org/10.1016/j.fluid.2010.11.014.

S11. Wang, H.; Jia, Y.; Wang, X.; Yao, Y.; Jing, Y. Physical–chemical properties of nickel analogs ionic liquid based on choline chloride. *J. Therm. Anal. Calorim.* **2014**, *115*, 1779–1785. https://doi.org/10.1007/s10973-013-3398-3

S12. Gupta, A.; Loew, G.H.; Lawless, J. Interaction of metal ions and amino acids: possible mechanisms for the adsorption of amino acids on homoionic smectite clays. *Inorg. Chem.* **1983**, *22*(1), 111-120.

S13. Drago, R.S.; Meek, D.W.; Longhi, R.; Joesten, M.D. Spectrochemical studies of the primary alkylamine complexes of nickel (II) and an evaluation of the donor properties of amines. *Inorg. Chem.* **1963**, *2*(5), 1056-1060.

S14. Khan, M.; Bouet, G.; Tanveer, R.; Ahmed, R. Stability constants of thiocyanato complexes of cobalt(II), nickel(II) and copper(II) in methanol. *J. Inorg. Biochem.* **1999**, *75*(2), 79-83. ISSN 0162-0134, https://doi.org/10.1016/S0162-0134(99)00033-1.

S15. Tsuruoka, M.; Hara, J.; Hirayama, A.; Sugimoto, M.; Soga, T.; Shankle, W.R.; Tomita, M. Capillary electrophoresis-mass spectrometry-based metabolome analysis of serum and saliva from neurodegenerative dementia patients. *Electrophoresis* **2013**, *34*(19), 2865-2872. doi: 10.1002/elps.201300019. Epub 2013 Sep 6. PMID: 23857558.

S16. Nakamura, Y.; Kodama, H.; Satoh, T.; Adachi, K.; Watanabe, S.; Yokote, Y.; Sakagami, H. Diurnal changes in salivary amino acid concentrations. *In Vivo* **2010**, *24*(6), 837-842. PMID: 21164041.

S17. Zappacosta, B.; Manni, A.; Persichilli, S.; Scribano, D.; Minucci, A.; Lazzaro, D.; De Sole, P.; Giardina, B. HPLC analysis of some sulphur compounds in saliva: comparison between healthy subjects and periodontopathic patients. *Clin. Chim. Acta* **2003**, *338*(1–2), 57-60. ISSN 0009-8981.

S18. Paul, B.D.; Smith, M.L. Cyanide and thiocyanate in human saliva by gas chromatography-mass spectrometry. *J. Anal. Toxicol.* **2006**, *30*(8), 511-515.
S19. Khan, M.A.; Cronier, D.; Bouet, G.; Vierling, F. Spectrophotometric investigation of nickel(II) chloro complexes in alcoholic solutions. *Transition Met. Chem.* **1995**, *20*, 369-371.
S20. Grootveld, M.; Bell, J.K.; Halliwell, B.; Aruoma, O.I.; Bomford, A.; Sadler, P.J. Non-transferrin-bound iron in plasma from patients with idiopathic haemochromatosis. Characterisation by high performance liquid chromatography and nuclear magnetic resonance. *J. Biol. Chem.* **1989**, *246*(8), 4417-4422.
S21. Silwood, C.J.L.; Grootveld, M. Chemical nature of implant-derived titanium(IV) ions in synovial fluid. *Biochem. Biophys. Res. Commun.* **2005**, *330*, 784-790.
S22. Silwood, C.J.L.; Grootveld, M. Evaluation of the speciation status of aluminium(III) ions in isolated osteoarthritic knee-joint synovial fluid. *Biochim. Biophys. Acta* **2005**, *1727*, 327-339.
S23. Silwood, C.J.L.; Grootveld, M. ^{1}H and ^{51}V NMR investigations of the molecular nature of implant-derived vanadium ions in osteoarthritic knee-joint synovial fluid. *Clinica Chimica Acta* **2007**, *380*, 89-99.
S24. Silwood, C.J.L.; Chikanza, I.; Tanner, K.E.; Shelton, J.; Bowsher, J.; Grootveld, M. Investigation of the molecular nature of low-molecular-mass cobalt(II) ions in isolated osteoarthritis synovial fluid. *Free Rad. Res.* **2004**, *38*(6), 561-571.
S25. Silwood, C.J.L.; Grootveld, M. Examination of the molecular nature of low-molecular-mass chromium(III) ions in isolated osteoarthritic synovial fluid. *J. Inorg. Biochem.* **2005**, *99*, 1390-1400.
S26. Solomons, N.W.; Viteri, F.; Shuler, T.R.; Nielsen, F.H. Bioavailability of nickel in man: effects of foods and chemically-defined dietary constituents on the absorption of inorganic nickel. *J. Nutr.* **1982**, *112*(1), 39-50.
S27. Darsow, U.; Fedorov, M.; Schwegler, U.; Twardella, D.; Schaller, K.H.; Habernegg, R.; Fromme, H.; Ring, J.; Behrendt, H. Influence of dietary factors, age and nickel contact dermatitis on nickel excretion. Contact Derm. **2012**, *67*(6), 351-358.
S28. Patriarca, M.; Lyon, T.D., Fell, G.S. Nickel metabolism in humans investigated with an oral stable isotope. *Am. J. Clin. Nutr.* **1997**, 66(3), 616-621.
S29. EFSA Panel on Contaminants in the Food Chain (CONTAM); Schrenk, D.; Bignami, M.; Bodin, L.; Chipman, J.K.; Del Mazo, J.; Grasl-Kraupp, B.; Hogstrand, C.; Hoogenboom, L.R.; Leblanc, J.C.; Nebbia, C.S.; *et al.* Update of the risk assessment of nickel in food and drinking water. *EFSA J.* **2020**, 18(11), e06268. doi: 10.2903/j.efsa.2020.6268. PMID: 33193868; PMCID: PMC7643711.
